# Short-term clinical and immunologic effects of poly-gamma-glutamic acid (γ-PGA) in women with cervical intraepithelial neoplasia 1 (CIN 1): A multicenter, randomized, double blind, phase II trial

**DOI:** 10.1371/journal.pone.0217745

**Published:** 2019-06-20

**Authors:** Hyun-Woong Cho, Young-Chul Park, Moon-Hee Sung, Jong Sup Park, Tae Jin Kim, Seok Ju Seong, Chi Heum Cho, Jae Kwan Lee

**Affiliations:** 1 Department of Obstetrics and Gynecology, Guro Hospital, College of Medicine, Korea University, Seoul, Korea; 2 Bioleaders Corporation, Daejeon, Korea; 3 Department of Bio and Fermentation Convergence Technology, Kookmin University, Seoul, Korea; 4 Department of Obstetrics and Gynecology, Seoul St. Mary's Hospital, The Catholic University of Korea College of Medicine, Seoul, Korea; 5 Department of Obstetrics and Gynecology, Konkuk University School of Medicine, Seoul, Korea; 6 Department of Obstetrics and Gynecology, CHA Gangnam Medical Center, CHA University, Seoul, Korea; 7 Department of Obstetrics and Gynecology, Keimyung University Dongsan Medical Center, Daegu, Korea; Seoul National University Bundang Hospital, REPUBLIC OF KOREA

## Abstract

**Objective:**

The aim of this study was to investigate the short-term efficacy and safety of Poly-gamma-glutamic acid (γ-PGA) and the immunologic changes in patients with CIN 1.

**Methods:**

Participants were randomly assigned to one of two groups and orally treated with placebo or 1,500 mg of γ-PGA for 4 weeks. The primary endpoint of the study was histologic regression rate of CIN 1 at 12 weeks between γ-PGA and control groups. The secondary endpoints were HPV clearance and change in immune responses.

**Result:**

From April 2013 to December 2015, 195 patients participated in the study. In the intention-to-treat analysis, 42 (42.4%) of the women who received γ-PGA experienced histologic remission versus 26 (27.1%) in the control group, with a statistically significant difference (p = 0.018). In the γ-PGA group, HPV clearance was found in 37 (43.5%) of 85 patients infected with high-risk HPV, showing a significant difference compared to the control group, in which 20 (26.7%) of 75 patients exhibited HPV clearance (p = 0.026). However, there was no significant difference between the two groups in the change of NK cell activity, major histocompatibility complex (MHC) class II CD8 count, and CD56 count.

**Conclusion:**

γ-PGA showed a short-term therapeutic effect on CIN 1 and high-risk HPV infection. It is a non-invasive, promising oral medication for women with these conditions.

**Trial registration:**

Clinical Trials NCT01826045.

## Introduction

Cervical intraepithelial neoplasia (CIN) can be a precursor to invasive cervical cancer and is the most common significant gynecologic disease in women of reproductive age [1]. In general, conservative management is recommended for patients with CIN 1 because they are considered to have a transient HPV infection, and the condition spontaneously regresses within 2 years in more than 60% of cases [2–4]. However, 16–30% of patients had persistent low-grade lesion and 4–10% progressed to high-grade lesion. If the disease is persistent or progressive, invasive procedures may be required [2, 5–7]. In addition, most patients want more active treatment because repeated follow-up tests are stressful and can negatively affect the patient's daily life [8]. Therefore, development of a new agent to treat CIN 1 is necessary.

γ-PGA is a non-toxic, water-soluble, and biodegradable substance known as an immune modulator. In vivo tumor regression activity of high-molecular weight γ-PGA from Bcaillus subtilis (chungkookjang) was observed with high levels of natural killer (NK) cell-mediated cytotoxicity and interferon (IFN)-gamma secretion in mice [9, 10]. γ-PGA has been reported to activate macrophages and dendritic cells (DC) in the small intestine mucosa, ultimately leading to antitumor and antiviral effects by toll-like-receptor 4 (TLR4)-induced NK cells and cytotoxic T cell activation [9, 11, 12]. We previously reported that γ-PGA may be helpful for cytological regression and reduction of viral load in patients with high-risk HPV-positive vaginal intraepithelial neoplasia [13]. Phase 1 clinical trials have shown that oral administration of γ-PGA is safe for humans and promotes cell-mediated immunity by increasing NK cell activity [14]. In the present study, the short-term clinical efficacy and immune response of oral administration of high-molecular-weight γ-PGA (MW 2,000 kDa) in women with cervical intraepithelial neoplasia 1 (CIN1) were investigated in a multi-center, randomized, double blind, placebo control, parallel designed, phase 2b study.

## Materials and methods

The study was carried out according to the principles expressed in the Declaration of Helsinki and Korean good clinical practice (KGCP). The study received institutional review board (IRB) of Korea university guro hospital (2013GR0058) and was registered as clinicaltrials.gov study NCT01826045. Each subject gave written consent for their participation. The Protocol for this Trial and supporting CONSORT checklist are available as supporting information (S1 Text, S2 Text and S1 Table).

### Participants

From April 2013 to December 2015, women were recruited from six medical centers in Korea. Women were eligible if they met the following inclusion criteria: aged between 20 and 49 years and histologically confirmed with CIN 1 by colposcopic punch biopsy. Women with other malignancies or those who were immunosuppressed or pregnant were excluded from the study. All subjects were required to use effective contraception during the study.

### Sample size

Based on published literature, we assumed that 31% of CIN 1 cases would resolve spontaneously, and a clinically significant difference was estimated at 20% [2, 15–17]. A sample size of 80 in each arm was planned to provide at least 80% power to detect a 20% difference in response rates using a one-sided chi-square test. Considering a potential dropout rate of 20%, the total sample size required for the study was 200 women, with 100 in each group. Based on a study by Jung et al., the number of subjects was calculated using the two-stage optimal design method [18]. In the first step, 43 women were allocated to each group. If the between-group difference in the number of patients who experienced disease regression by the end of the first stage was <2, there was considered to be no effect. Otherwise, an additional 37 patients were recruited into each group. In that case, it was judged that there was no effect when there was a <11-person difference between groups in the number of patients with regressed disease.

### Study design

Subjects were enrolled and randomized to either placebo or γ-PGA group. Randomization was performed using a computer-generated assignment scheme designed and performed in a masked manner by the data coordinating center. Participants were assigned to a group by block randomization with an appropriate block size. After randomization, subjects received oral administration of 100 mL of a syrup containing 1000 mg γ-PGA or placebo once daily for 4 weeks. The dose was based on Phase 1 clinical trial results: immunohistochemical (IHC) staining showed that the γ-PGA group had a higher level of NK cell activity than the placebo group, and the low dose group (1.5 g/day) showed a higher level than the high dose group (3.0 g/day) at 3 and 5 days after administration [14]. At the baseline visit, all eligible subjects underwent a medical history and physical examination. Participants were monitored for adverse drug reactions and tolerability, and blood samples were collected to check for immune responses every 4 weeks after treatment initiation. Twelve weeks after study entry, all subjects underwent colposcopic biopsy, HPV testing, and immune activity testing. At the colposcopy visit, biopsies were taken from suspected areas according to the protocol of this study based on American Society for Colposcopy and Cervical Pathology recommendations by expert gynecologic oncologist [19, 20]. If no abnormalities were seen, the examiner was directed to take two random biopsies. Certified pathologists at each institution independently reviewed entry and exit cervical diagnostic punch biopsy results using published criteria. HPV tests and immunological tests were performed in a central laboratory because results may differ across laboratories depending on the testing method or procedure. Compliance after enrollment was measured as the percentage of dispensed pills taken between visits. Subjects who took fewer than 80% of pills after randomization were considered noncompliant and were removed from the study, but they were included in the intention-to-treat analysis at study completion. Adverse drug effects were evaluated every 4 weeks based on preferred terminology.

### Assessment of outcomes

The primary outcome was disease regression at three months after diagnosis. Secondary outcomes were high-risk HPV status, viral load of HPV, immune response, and safety data. HPV testing was performed at the 12-week visit using the Digene Hybrid Capture (HC) 2 High-Risk HPV DNA Test and Anyplex^TM^ II HPV28 detection at the Department of Pathology, Cheil General Hospital. All specimens were tested with HC2 and Anyplex II HPV detection kits according to the manufacturer’s protocol.

Immune status was evaluated at each visit (every 4 weeks) using an NK cell activity assay and measurement of the MHC class II CD8 and CD56 populations. Laboratory protocol for assay of the cytotoxic activity of NK cells was adapted from a previous phase I study [14]. Peripheral blood mononuclear cells (PBMCs) (4 x10^5^) from participants were cultured in the presence or absence of γ-PGA (0.1mg/mL) for 8 hours and then stimulated with K562 tumor cells (2 x10^5^) for an additional 7 hours in the presence of monensin or brefeldin A, which detect surface CD107a and intracellular interferon-γ (IFN-γ), respectively. Cells were stained with CD56 (BeckmanCoulter, CA, USA), CD3, IFN-γ, CD107a (BD Biosciences, CA, USA), CD14, and CD19 (Biolegend, CA, USA) for flow cytometry, and CD56dim, CD3-, CD14-, CD19- cells were gated as previously described [21].

To measure phenotypic changes in peripheral blood mononuclear cells (PBMNCs), the cells were separated by a density gradient method using a boiling solution of Ficoll-Hypaque. Peripheral blood samples were superimposed on Histopaque (specific gravity 1.077 g/mL sterile-filtered, Sigma) solution using SEPMATE tubes (StemCell, 15450) and were centrifuged at 1200 × g for 10 minutes. The upper layer was removed and washed with PBS + 2% to separate mononuclear cells. PBMNCs were treated with FACS buffer (0.1% bovine serum albumin, 0.01% sodium chloride) containing fluorescent-labeled antibodies (CD3-Percp-eFluor 710, CD8-FITC, CD56-PE, CD14-APC-eFluor 780, and CD19-APC-eFluor 780, CD107a-AlexaFluor 647) and left on ice for 30 minutes to confirm the change in peripheral blood mononuclear cell immunity phenotype using a BD truCountTM method (BD Bioscience, San Jose, CA, USA). Fluorescent-stained cells were washed twice with FACS buffer and fixed with phosphate buffer containing 2% paraformaldehyde. Cell fluorescence was measured and analyzed using a Gallios flow cytometer (Beckman Coulter, Brea, CA, USA).

### Statistical analysis

The primary efficacy variable was based on histologic analysis of biopsy-derived cervical tissue for the presence or absence of CIN 1. Histologic resolution was defined as an outcome of “normal.” In general, continuous variables were summarized by descriptive statistics, including number, mean, median, standard deviation, minimum, and maximum. Categorical variables were summarized by number and percentage. All percentages were calculated using non-missing data as the denominator. All statistical tests were 2-sided with a 0.05 level of significance. The demographic and baseline diagnostic data between placebo and γ-PGA groups were calculated. The regression rate, which is the primary endpoint, was analyzed with a one-sided test at a significance level of 5%. Type-specific HPV infection, viral load of HPV, NK cell activity, CD8 count, and CD56 count were measured at 12 weeks of follow-up and compared with the baseline values. A generalized estimating equation or generalized linear mixed model was used to identify differences and time and intergroup interactions. Analyses of general characteristics and histopathologic response by treatment group were completed using intention-to-treat (ITT) methodology and by imputing histologic biopsy results for women with missing 12-week measurements. Imputed values were found using worst-rank score (i.e., women without results were still associated with their baseline result [CIN 1]).Patients with missing data were excluded from statistical analysis of HPV clearance and immunologic response. All statistical analyses were performed using PC SAS 6.12 (SAS Institute, Cary, NC).

## Results

**Fig 1:**
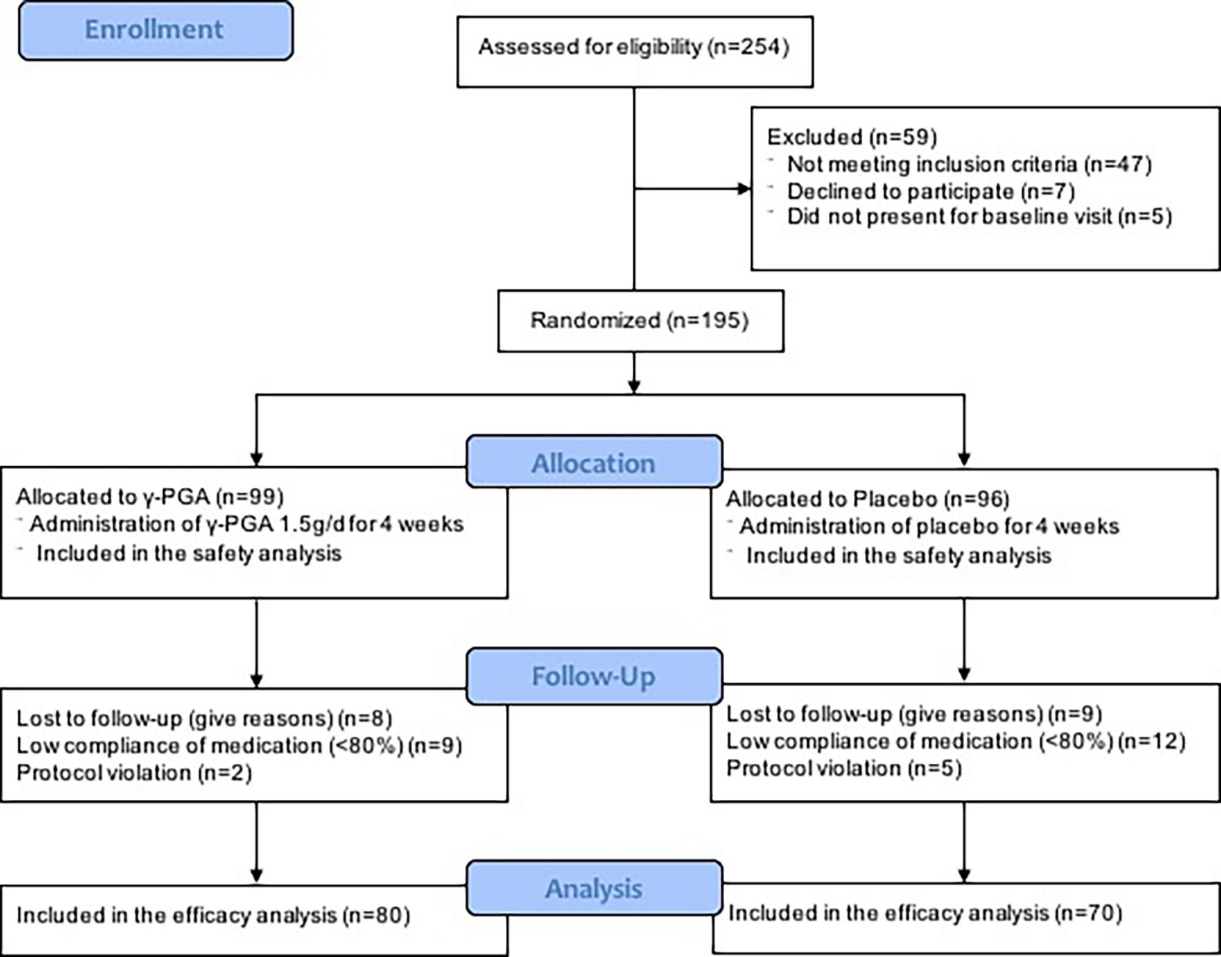
Flow diagram of the study.

From April 2013 to December 2015, 195 subjects meeting inclusion criteria were enrolled from six medical centers in Korea (Fig 1). Forty-seven women did not meet the inclusion criteria, seven refused to participate, and five did not appear at the baseline visit. A total of 195 baseline surveys were conducted. During the study, 45 women were excluded: 17 for loss to follow-up and 28 for protocol violations. However, as analysis was based on the ITT principle, all excluded subjects were included in the final analysis.

The general characteristics of the two groups are described in Table 1. Median age was 32 years in both groups. In addition, there were no differences between the two groups in other general characteristics, including smoking, a factor associated with CIN.

**Table 1:** General characteristics of study participants.

		PGA group (n = 99)	Placebo group (n = 96)	p-value
		Mean±SD*/N (%)	Mean±SD /N (%)	
Age		32.76 ± 8.03	32.17±7.61	0.620
BMI (kg/m^2^)		21.4±1.32	20.5±1.47	0.383
Menarche age		13.3 ±1.49	13.34±1.57	0.880
Marital status	Yes	48 (48.5)	41 (42.7)	0.479
	No	51 (51.5)	55 (57.3)	
Parity	0	55 (55.6)	65 (67.7)	0.081
	>1	44 (44.4)	31 (32.3)	
Contraception	Yes	59 (59.6)	52 (54.2)	0.410
	No	40 (40.4)	44 (45.8)	
Alcohol	Yes	69 (69.7)	71 (74.0)	0.576
	No	30 (30.3)	25 (26.0)	
Smoking	Smoker	17 (17.2)	10 (10.4)	0.223
	Ex-smoker	3 (3.0)	6 (6.3)	
	Non-smoker	79 (79.8)	80 (86.3)	
HPV status	HPV 16/18	21 (21.2)	16 (16.7)	0.190
	Other hrHPV^†^	77 (77.8)	75 (78.1)	
	Negative	1 (1.0)	5 (5.2)	

*SD: standard deviation

^†^Other hrHPV: 26, 31, 33, 35, 39, 45, 51, 52, 53, 56, 58, 59, 66, 68, 69, 73, 82

The primary endpoint of this study was histopathologic response in women with CIN 1 (Table 2). In the ITT population, there was a statistically significant difference of histologic remission between the γ-PGA and placebo groups (42.4% vs. 27.1%, p = 0.018). However, there was no significant difference between the two groups with regard to progression to HSIL (18.8% vs 16.2%, p = 0.442).

**Table 2:** Histopathologic responses by treatment group.

	PGA (n = 96)		Placebo group (n = 99)		p-value
	n	%	n	%	
Remission*	42	42.4	26	27.1	0.018
Progression to HSIL^†^	18	18.8	16	16.2	0.442

*Remission is defined as complete histologic remission

^†^HSIL is defined as CIN2, CIN3

The type-specific clearance and viral load of HPV are described in Table 3. Under per protocol analysis, the resolution rate of any high-risk HPV infection was significantly higher in the γ-PGA group compared with the placebo group (43.5% vs. 26.7%, p = 0.026). On the other hand, clearance of HPV16 and HPV18 infections were higher in the γ-PGA group, but the difference was not statistically significant. There was no difference in the HPV infection viral load between the γ-PGA and placebo groups at baseline and at the 12-week visit when classified as negative, low, and high. There was no difference in the reduction of viral load between the two groups when the reduction was defined as high to low/negative or low to negative.

**Table 3:** Type-specific clearance of HPV infection (Anyplex II HPV 28) and its viral load (RLU/CO) value measured by hybrid capture assay between the PGA and placebo groups.

HPV clearance		PGA group (n = 86)		Placebo group (n = 92)		p-value
		n	%	n	%	
HPV16 (N = 9)		7	43.8	2	18.2	0.228
HPV18 (N = 4)		3	75.0	1	33.3	0.486
Any hrHPV* (N = 57)		37	43.5	20	26.7	0.026
Viral load		PGA group (n = 86)		Placebo group (n = 92)		p-value
		n	%	n	%	
Baseline	Negative	12	14.0	8	8.7	0.255
	Low (<100 RLU)	0	0	2	2.1	
	High (>100 RLU)	74	86.0	82	89.1	
12 weeks	Negative	24	27.9	18	19.6	0.190
	Low (<100 RLU)	4	4.7	4	4.3	
	High (>100 RLU)	58	67.4	70	76.1	
Reduction^†^		16	18.6	12	13.0	0.402

*high risk HPV (hrHPV) genotype: 16, 18, 26, 31, 33, 35, 39, 45, 51, 52, 53, 56, 58, 59, 66, 68, 69, 73, 82

^†^Reduction is defined as a change from high to low or positive to negative

NK cell activity and MHC class II CD8 and CD56 counts were performed at baseline and at 4 weeks, 8 weeks, and 12 weeks to check immune responses. In the γ-PGA group, NK cell activity increased at 4 and 8 weeks, but there was no statistically significant difference between the two groups (Table 4). There was also no significant difference between the two groups in the change from baseline to 12 weeks in CD8 (p = 0.053) and CD56 (p = 0.143) count.

**Table 4:** Immunologic response between the PGA and placebo groups.

		Placebo (n = 86) Mean (SD)	PGA (n = 92) Mean (SD)	p-value
Baseline	NK cell activity	7.17 (8.71)	7.31 (8.28)	0.909
	PBMNCs CD8	17.46 (7.31)	18.03 (8.7)	0.642
	PBMNCs CD56	16.71 (11.65)	14.45 (9.83)	0.165
4 weeks	NK cell activity	8.81 (9.3)	9.13 (8.95)	0.687
	PBMNCs CD8	17.39 (8.09)	17.56 (7.73)	0.714
	PBMNCs CD56	14.35 (10.12)	13.31 (8.18)	0.427
8 weeks	NK cell activity	8.65 (8.95)	9.48 (10.05)	0.557
	PBMNCs CD8	19.06 (8.48)	16.93 (7.54)	0.112
	PBMNCs CD56	14.89 (9.67)	14.53 (8.87)	0.774
12 weeks	NK cell activity	9.23 (9.28)	8.93 (8.8)	0.833
	PBMNCs CD8	18.97 (7.2)	17.58 (7.99)	0.241
	PBMNCs CD56	13.92 (8.17)	14.03 (9.83)	0.868
Difference (12 weeks-baseline)	NK cell activity	2.06 (8.58)	1.49 (6.47)	0.621
	PBMNCs CD8	1.51 (6.77)	-0.71 (8.26)	0.053
	PBMNCs CD56	2.8 (11.1)	0.45 (10.01)	0.143

According to the safety evaluation, 76 (38.9%) subjects had 157 adverse events: 34 patients (35.4%) in the placebo group and 42 (42.4%) in the γ-PGA group. The most common adverse effects in the placebo group were headache, cough, and dyspepsia, whereas those in the γ-PGA group were premenstrual pain, headache, and cough. There was no significant difference in the incidence of adverse effects between the two groups. Adverse events related to the drug were very infrequent in both the placebo group (4 subjects, 5.13%) and the γ-PGA group (4 subjects, 4.67%), and no severe adverse events were reported.

## Discussion

Our data suggest that γ-PGA is efficacious in promoting histologic regression and clearance of high-risk HPV in patients with CIN 1 in the short term. To our knowledge, γ-PGA is the only oral medication proven to be efficacious for regression of CIN I and clearance of high-risk HPV in a randomized controlled trial (RCT). γγ-PGA is extracted from the traditional Korean food Chungkukjang and it exhibits excellent tolerability and safety.

In this study, the histologic regression rate was significantly higher in the γ-PGA group than in the placebo group (42.4% versus 27.1%). The spontaneous regression rate of the placebo group was consistent with previous studies that reported 28% to 31% at 3 months after diagnosis [15, 16]. In this study, γ-PGA showed statistically significant improvement in histologic regression rate. Although 3 months is a relatively short period of time for follow-up, previous studies showed that most patients with disease that improved early remain in that condition for a long period of time. In a cohort study of 1,001 patients with CIN 1, 330 patients regressed to normal at 6 months. Of those with negative pathology at 6 months, 200 (80%) remained negative at 12 months. At 12 months, 42 (16%) demonstrated low grade pathology and 9 (4%) progressed to high grade lesions [2]. In another cohort, the rate of spontaneous histologic regression in CIN 1 patients at 15 weeks was 28% [16]. In a study of 20,319 patients with CIN 1, the 5-year risks of CIN 2 following negative follow-up Pap test and co-test were 5.4% and 1.1%, respectively [22]. In a recent study of therapeutic vaccine in patients with CIN 3, Pap and HPV test improved in 6 of 9 patients at 12 weeks, and histologic results improved to normal in all patients at 20 weeks and 36 weeks [23].

Unfortunately, PGA did not reduce progression of CIN 1 in this study. However, CIN1 remission is also meaningful. In several studies investigating treatment preferences, most patients desire more active treatment rather than only follow-up visits; repeated examinations and follow-up visits were associated with anxiety and discomfort, which negatively affected their daily lives [24–31]. According to Health Insurance Review and Assessment service National Patient Sample (HIRA NPS) data of Korea, 13% of patients diagnosed with CIN1 underwent invasive procedures such as conization or ablative treatments [32]. In an unpublished survey of Korea, the length of follow-up period for CIN1 was 1–3 months for 32% of patients, 4–6 months for 46% of patients, 7–12 months for 17% of patients, and more than 1 year for 4% of patients [32]. PGA therapy may decrease the number of invasive procedures or reduce the follow-up duration (<6 months) in patients with CIN 1. This will reduce medical costs, adverse effects, and inconvenience caused by unnecessary treatment or follow-up tests. Although the difference in CIN1 remission is only about 15%, PGA treatment could be beneficial because of the high and increasing incidence of CIN 1. Furthermore, PGA seemed to have therapeutic effects on HPV infection and potentially has other more significant beneficial effects.

In this study, γ-PGA was associated with a significantly higher clearance rate of high-risk HPV infection compared with placebo (difference of 17%). However, there was no significant difference in the type-specific HPV clearance rate except for a borderline significant difference in HPV 53 (γ-PGA vs. placebo: 55.6% vs. 13.3%, p = 0.061). Similar results were reported in a previous study, suggesting that the HPV therapeutic vaccine cleared HPV infections and CIN lesions. Four of nine patients vaccinated with GX-188E cleared their HPV infection in 12 weeks, and 6 patients cleared infections in 20 weeks [23]. Although there was no statistical significance, these data showed a tendency of increased viral clearance rate for HPV 16 (43.2% in γ-PGA versus 18.2% in placebo) and 18 (75.0% in γ-PGA versus 33.3% in placebo). The HPV viral load was also analyzed using Hybrid Capture II measurements, but no significant differences were observed between the two groups.

NK cell activity and CD8 and CD56 cell counts were measured at 4 and 8 weeks after treatment to evaluate the mechanism of γ-PGA action. Kim et al. reported that NK cells and IFN-gamma play important roles in the anticancer activity of γ-PGA [9]. Unlike in a previous study, there were no significant differences in NK cell activity, CD8 count, and CD56 count between the γ-PGA and placebo groups. The reason the current study is inconsistent with previous studies may be that the duration of γ-PGA administration was not long enough for NK cell activity to increase significantly. The effect of γ-PGA on NK cell activity at the 12-week visit may have been weak because the duration of γ-PGA administration was 4 weeks shorter than in the previous study. In this study, NK cell activity showed a tendency to increase at the 8-week visit, but decreased at the 12-week visit. This should be fully considered in subsequent studies with respect to changes in the dose and duration of γ-PGA administration.

Many studies investigating treatments for CIN and HPV infection have been reported. Several therapeutic vaccines targeting high risk HPV have been tested in clinical trials and recent phase IIb trials have shown regression of high-grade CIN after vaccine treatment [33]. Studies on topical agents such as fluorouracil (5-FU), imiquimod, and trichloroacetic acid have been reported. 5-FU (relative risk of regression = 1.62, 95% confidence interval 1.10–2.56) and Imiquimod (histologic regression: 73% of the experimental group versus 39% of the placebo group, p = 0.009) [34, 35]. Cyclooxygenase (COX)-2 inhibitors, retinoid, folate, beta-carotene, indole-3-carbinol, and immunotherapy agents including ZYC101a, MVA E2, and HspE7 have been studied as medical therapy for CIN [36]. Although many agents have been studied, most therapeutic agents are not for oral administration and relatively few studies have been conducted on CIN 1. To date, imiquimod, intravaginal gel 851B, therapeutic vaccine, γ-PGA, beta carotene, and folic acid have been studied for CIN 1.

γ-PGA extracted from chungkukjang (*Bacillus subtilis*) is a biopolymer used in a wide range of applications, including cosmetics, bone care, and nanoparticles for drug delivery. Recent studies have shown that it can be used as an immune-stimulating agent, especially the high molecular weight form [37]. High molecular weight γ-PGA induced type I interferon activity and showed antiviral activity against influenza virus in mice [38]. Intranasal administration of γ-PGA was followed by production of antiviral cytokines including interferon-beta and interleukin-12, as well as enhanced activation of NK cells and antigen-specific cytotoxic T lymphocytes [11]. In addition to antiviral activities, γ-PGA is a potential antitumor medication. In mice bearing MHC class I-deficient tumors, oral administration of γ-PGA induced NK cell–mediated antitumor activity, which is mediated by TLR4 [9, 12]. Based on these studies, a clinical trial was conducted to evaluate the effect of γ-PGA on human immunity, and a statistically significant increase in NK cell cytotoxicity was observed in the high-dose γ-PGA group [14]. In a retrospective study on γ-PGA therapy for vaginal intraepithelial neoplasia (VAIN), γ-PGA showed potential efficacy for cytologic regression and HPV viral load reduction [13].

The current study also has strengths. This is the first RCT to demonstrate efficacy of γ-PGA as an oral medication in women with CIN 1. Second, γ-PGA has been shown to be effective in high-risk HPV infections. Third, several immune factors were analyzed including NK cell activity to elucidate the drug’s mechanism of action.

This study has some limitations. First, because only women with CIN 1 participated in the study, the efficacy of γ-PGA could not be determined in high-grade CIN. Second, follow-up duration was not long enough to show the long-term effects of the drug. Finally, the sample size was not sufficient to represent the general population of patients with CIN 1. In addition, this study might be not adequately powered under the assumptions that were given, because the remission rate of the placebo group was slightly less than 30 percent (27%), and the difference between the two groups was slightly lower than 20% (15%). However, 45.8% (42 patients) of the experimental group and 30.2% (26 patients) of the control group were regressed to normal of 178 patients (92 patients in the PGA group and 86 patients in the placebo group), except those who did not undergo biopsy at the 12-week visit. The difference in the regression rate between the two groups was statistically significant at 15.5% (p = 0.025). Therefore, the assumptions of the sample size computation of this study may be considered partially realized.

## Conclusion

γ-PGA showed a short-term therapeutic effect in CIN 1 and high-risk HPV infection. It is a promising new oral medication for women with CIN 1 and women with high-risk HPV infection. However, no significant effect was observed on specific HPV types, such as 16 and 18, and the immunologic response did not change with γ-PGA treatment. Therefore, further studies of the mechanism of γ-PGA activity are needed.

## Supporting information

S1 Text Study protocol. (DOCX)

S2 Text Study protocol Korean. (PDF)

S1 Table CONSORT 2010 checklist. (DOC)
