# Supplementary material for: Short-term clinical and immunologic effects of poly-gamma-glutamic acid (γ-PGA) in women with cervical intraepithelial neoplasia 1 (CIN 1): A multicenter, randomized, double blind, phase II trial
Source: PLoS One. 2019 Jun 20;14(6):e0217745. doi: 10.1371/journal.pone.0217745 (PMC6586279; doi:10.1371/journal.pone.0217745)
Supplement: S1 Text — (DOCX) [file pone.0217745.s001.docx]

**CLINICAL STUDY PROTOCOL**

**A multi-center, randomized, double blind, placebo control, parallel design, phase 2b trial to evaluate the efficacy and safety of PGA (Poly-gamma-glutamic Acid) for the fertile women with Cervical Intraepithelial Neoplasia (CIN I)**

**Principal Investigator**:

| Jong-Sup Park, MD, PhD | The Catholic University of Korea Seoul St. Mary's Hospital |
| --- | --- |
| Jae-Kwan Lee, MD, PhD | Korea University Guro Hospital |
| Tae-Jin Kim, MD, PhD | Cheil General Hospital and Women’s Healthcare Center |
| Chi-Heum Cho, MD, PhD | Keimyung University Dongsan Medical Center |
| Seok-Ju Seong, MD, PhD | CHA Gangnam Medical Center, CHA University |
| Yong-Soo Park, MD, PhD | Gangseo MizMedi Hospital |

**Date of Issue**: August 05.2015

**Document Number**: UMT2012-BL-PGA-01

**Version Number**: 4.8

The information in this document is the property of BioLeaders Corp. and is confidential. Neither the document nor the information contained therein may be reproduced or disclosed outside BioLeaders Corp. without the written consent of the company.

**ABBREVIATIONS AND DEFINITIONS**

CD: Cluster differentiation CD8: Killer T cell

CD56: NK cell

MHC: class II Major Histo-compatibility Complex class II INF-γ: Interferon-γ

NK cell: Natural killer cell

TNF-α: Tumor necrosis factor-α

ECG: Electrocardiogram AE: Adverse event

ADR: Adverse Drug Reaction SAE: Serious adverse event

ITT: Intent-to-treat PP: Per-Protocol

ASCUS: Atypical squamous cells of undetermined significance LGSIL: Low grade squamous intraepitherial lesion

HGSIL: High grade squamous intraepitherial lesion CIN: Cervical intraepitherial neoplasia

HPV: Human papillomavirus

ASCCP Consensus Guideline: American Society for Colposcopy and Cervical Pathology

**Protocol Outline**

| Study Title | A multi-center, randomized, double blind, placebo control, parallel design, phase 2b trial to evaluate the efficacy and safety of PGA (Poly-gamma-glutamic Acid) for the fertile women with Cervical Intraepithelial Neoplasia(CIN I) |
| --- | --- |
| Study Phase | Ⅱb |
| Study design | Randomized, Double-blind, Placebo-controlled, Parallel-group |
| Investigators/ Site Name | Jong Sup Park, MD, PhD / The Catholic University of Korea Seoul St. Mary's Hospital  Jae-Kwan Lee, MD, PhD / Korea University Guro Hospital  Tae Jin Kim, MD, PhD/ Cheil General Hospital and Women’s Healthcare Center  Chi-Heum Cho, MD, PhD/ Keimyung University Dongsan Medical Center  Seok Ju Seong, MD, PhD/ CHA Gangnam Medical Center, CHA University  Yong Soo Park, MD, PhD/ Gangseo MizMedi Hospital |
| Sponsor | BioLeaders Corporation |
| Purpose | This study is to compare the regression rate of Cervical Intraepithelial Neoplasia (CIN1) between the treatment group and the control group. |
| Investigational Product | - Experimental: Poly-gamma-glutamic acid 1,500 mg  - Comparator: Placebo  - Dosage schedule: 1,500 mg in 100mL po q.d. before bedtime for 4 weeks (28 days) |
| Indication | Women aged 20-49 years with HPV positive CIN 1 |
| Inclusion Criteria | 1. Fertile female aged between 20 and 49  2. Patients who are diagnosed as Cervical intraepithelial neoplasia 1 (CIN1)  3. Patients who are infected with HPV (Human Papilloma Virus) positive (+)  4. White Blood Cell Count(WBC) over 4 10^3^/ul, Hemoglobin above over 9.0g/dL Platelet over 150 10^3^/uL and ANC (Absolute Neutrophil Count) over 1,500 10^6^/L  5. AST (Aspartate Aminotransferase) no less than 4 times higher than normal ALT (Alanine Aminotransferase) no less than 4 times higher than normal  6. Normal for EKG (Electrocardiography) and no active disease detected trough chest X-ray  7. Be informed of the nature of the study and will give written informed consent |
| Exclusion Criteria | 1. Malignant tumor in any organ other than cervical intraepithelial neoplasia  2. Active liver disease, immune disorder and severe renal failure  3. Leukemia, collagenosis, sclerosis, autoimmune disease, clinically significant allergic disease (mild allergic symptom not required medicine excluded)  4. Diagnosed diabetes  5. Taking any of followings affecting immunological reaction within 7 days (Glucocorticoid, vitamins, health food and oriental medicine etc)  6. Pregnancy and breastfeeding  7. Registered in other clinical trials  8. Patients whom the investigator considers inappropriate to participate in the study |
| Sample Size   \| **군 구성** \| **용량** \| **임상시험 대상자 수** \| **예상 치유율** \| **치유예상**  **임상시험 대상자 수** \| **투약기간/관찰기간** \| \| --- \| --- \| --- \| --- \| --- \| --- \| \| 위약군 \| - \| 80명 \| 31% \| 24.8명 \| 4주간 1일 1회 투약 및  8주간 추가 관찰 \| \| 시험군 \| 1,500mg  /day \| 80명 \| 51% \| 40.8명 \| \| 총 \|  \| 160명 \|  \|  \|  \| | Total 200 patients, 100 patients per group.  Based on published literature, we assumed that 31% of the cases of CIN 1 would resolve spontaneously, and a clinically significant difference was estimated at 20%. 80 patients per group.   \| Group \| Dose \| Patient \| Expected response Rate \| Expected response patient \| Duration of treatment/Observation \| \| --- \| --- \| --- \| --- \| --- \| --- \| \| Placebo \| - \| 80 \| 31% \| 24.8 \| administered once daily for 4 weeks /  observation for 8 weeks \| \| PGA \| 1,500mg/day \| 80 \| 51% \| 40.8 \| \| Total \| - \| 160 \|  \|  \|  \|   Cervical intraepithelial neoplasia (CIN) can act as a precursor to invasive cervical cancer and is the most common significant gynecologic disease in women of reproductive age. study, we investigated clinical efficacy and immune response of oral administration of γ-PGA from women with cervical intraepithelial neoplasia 1 (CIN 1) in phase 2b study  H_0_ : p_1_≦p2 vs. H_1_ : p_1_>p_2_  p_1_ : Response rate of treatment, p_2_: Response rate of Control  Expected response rate: PGA Group: 81%, Control Group: 51%  significance level : 0.05, statistical power : 80%, one-sided test  The Sample size is total 160 patients, 80 patients per group when the Single stage method.  If 20% drop out is considered, the sample is total 200 patients, 100 patients per group  The sample size is according to the Two stage optimal design (Jung(2008)) Method is shown in the following table.  [Jung(2008) Two-stage]   \| Stage \| Patient \| Difference between two groups (Treatment patient-Control patient) \| 제 significance level, statistical power \| \| --- \| --- \| --- \| --- \| \| 1  2 \| 43  80(43+37) \| +2  +11 \| 0.461, 0.800 \| |
| Study Duration | Approximately 48 months from IRB approval date |
| Study methodology | Patients were enrolled and randomized to either the placebo or the PGA group. Randomization was performed using a computer-generated assignment scheme designed and performed in a masked manner by the data coordinating center. The participants were assigned to a group by block randomization with an appropriate block size. After randomization, patients received oral administration of 100 mL of a syrup containing 1500 mg PGA or placebo once daily for 4 weeks. At the baseline visit, all eligible patients underwent a medical history and physical examination. Participants were monitored for drug adverse reactions and tolerability, and blood was collected to check for immune responses every 4 weeks after treatment initiation. Twelve weeks after study entry, all patients underwent colposcopic biopsy, HPV testing, and immune activity testing.  Patients visit 5times. -4W~ (Screening, visits1), 0 W (Baseline, visits 2), 4W (Observation, visits 3), 8W (Observation, visits 4), 12W (Closing, visits 5), the diagnosis of Cervical intraepithelial neoplasia associated with efficacy, evaluation of immune function, and safety tests. |
| Prohibited Medication | 1. Steroids: Betametasone, Dexametasone, Prednisolone, Hydrocortisone, etc., except when except when applied locally to skin or eyes.  2. Immunomodulators: growth hormone, EPO (Erythropoietine), albumin preparations, transfusion of whole blood and leukocyte components, etc  3. Others: Supplements and vitamin preparations other than clinical trial drugs. Chinese medicine |
| Criteria for removal and drop-out from study | 1. An acute response (allergic, hypersensitive reaction) to Investigational Product  2. If the investigator determines that continuing participation is not appropriate due to the occurrence of a "serious adverse reaction".  3. In the event of a breach of the selection / exclusion criteria or a violation of a significant clinical study protocol.  4. Patients will be able to withdraw from the trial at their own request.  5. In case of no observation due to absence of clinical test patients.  6. If the other tester determines that the investigator should be stopped. |
| Study evaluations | 1. Primary efficacy variable (Visit1, Visit5)  Comparison of Response rate PGA group and Placebo group at 12th week  Histologic resolution was defined as an outcome of “normal.”  Cervical intraepithelial neoplasia change step is as follows.  - Regression: CIN I → Normal  - Persistence: CIN I → CIN I  - Progression: CIN I → CIN II, CIN III, SCC(Squamous cell carcinoma)  Colposcopic biopsy (Visit1, Visit5)  2. Secondary efficacy variable (Visit1, Visit5)  - Cervical intraepithelial neoplasia evaluations  1) Reid Colposcopic Index  2) Pap Smear Test  3) HPV DNA Chip Test  4) HPV HC Ⅱ assay  - Immunologic response evaluations (Visit2, Visit3, Visit4, Visit5)  1) NK cell activity  2) MHC class II CD8, CD56 population  3. Safety evaluation (Visit2, Visit3, Visit4, Visit5)  Adverse events, signs of vitality, laboratory tests |
| Statistical analysis | 1. General principles  Analyses will be descriptive in nature. Descriptive analyses will include the number of observations, mean, standard deviation, median, range, and interquartile range for continuous variables and the number and percent for categorical variables.  2. Populations  - ITT(Intent-to-Treatment): Defined as all assigned patients. However, patients who did not take the investigational product at least once in the ITT group should be excluded from the analysis group.  - PP(Per Protocol): PP analyses exclude all protocol major violation, including anyone who did not adhere to treatment, switched groups, or missed measurements.  - The final evaluation will be based on the results of the ITT group analysis.  3. Safety analysis  After agreeing to participate in the study, the study will be defined as the whole box of randomized, at least one investigational product.  4. Primary efficacy variable analyses  In the baseline, 3 months after the administration of CIN I, the patient was judged to be cured to normal, and the response rate between the PGA group and the control group was compared.  The evaluation of the healing rate between the groups is based on the method proposed by Jung (2008). When the results were confirmed by selecting 43 patients in each group, as in the previous step, the difference in the number of patients. In the case of less than 2 persons (PGA group-Control group), it was judged that there is no effect of PGA, and the present study is terminated. If there are more than 2 difference in level 1, 37 patients per group are recruited. In case of more than +11 patients who were judged as Histologic resolution by 80 patients per group, Histologic resolution of CIN. It is judged that there is an effect of PGA. If there is less than +11, it is concluded that the effect of PGA is not ultimately.  5. Secondary efficacy variable analyses  Secondary efficacy variable 1: Reid Colposcopic Index  0-2 Score: Likely to be CIN I  3-4 Score: Overlapping lesion - likely to be CIN I or CIN II  5-8 Score: Likely to be CIN II  The comparison between the baseline scores and the difference of the scores after 12 weeks will be confirmed through the t-test or the Wilcoxon rank sum test. In addition, considering the multinomial distribution of the grading system (0-2, 3-4, 5-8 points), the cumulative or ordinal logistic model of the changes in the grading system after 12 weeks compared to baseline.  Secondary efficacy variable 2: Pap Smear Test  The reading standard is based **on the 2001 Bethesda System for Reporting Cervical Cytologic Diagnoses**. The results of the study were divided into 6 stages: Negative, ASC-US, ASC-H, LSIL, HSIL, and Invasive cancer (Cervical carcinoma). Considering the multinomial distribution, And the cumulative or ordinal logistic model is used to analyze changes in the classification stage 3 months after the baseline.  Secondary efficacy variable 3: HPV DNA Chip Test  The HPV DNA Chip Test is a test to determine the type of virus infection. It is classified as positive or negative according to the test result. GEE (Generalized Estimating Equation) or Generalized Linear Mixed Model is considered to determine time-dependent changes, group-to-group differences, and time-to-group interactions for baseline versus measured clearance.  Secondary efficacy variable 4: HPV HC Ⅱ assay  GEE (Generalized Estimating Equation) or Generalized Linear Mixed Model is considered to determine time-dependent changes, group-to-group differences, and time-to-group interactions for baseline versus measured clearance.  Secondary efficacy variable 5: NK cell activity  To compare the results of NK cell activity measured at 4 weeks, 8 weeks, and 12 weeks versus baseline (0 week), the mean difference between the baseline and 12-week NK cell levels was calculated as t - Black or Wilcoxon rank sum test.  Secondary efficacy variable 6: MHC class II CD8, CD56 population  GEE (Generalized Estimating Equation) or Generalized Linear Mixed Model is considered to determine time-dependent changes, group-to-group differences, and time-to-group interactions for baseline versus measured clearance.  6. Safety analyses  All safety data, including all adverse events collected from patients, clinical laboratory test results, 12-lead ECG, and vital signs (blood pressure and pulse rate) are performed.  1) Adverse events  Summarize the adverse events observed following administration of the investigational product. The number of cases and the number of patients who experienced an adverse reaction, abnormal drug response, SAE, death, adverse events n that caused discontinuation of clinical trial, and / or "other significant adverse events (OAE) do. The number of patients who received each of the abnormal responses was also summarized by body-organ system (SOC), recommended language, and maximum severity for each treatment group. In addition to the summary statistics, the incidence or incidence of adverse events in the group are analyzed using Chi-squared test, Fisher's Exact test, or Poisson test.  2) Vital signs and laboratory tests  The results were analyzed by Chi-squared test, Fisher's Exact test, or Poisson test for each group of 12-lead ECG abnormalities at each time point. A summary statistic is presented and, if necessary, GLM or GLMM methods are used to perform group comparisons. |

### [Study Flow Chart]

| Visit Type | Screening | Baseline &  Treatment | Observation | Observation | Closing |
| --- | --- | --- | --- | --- | --- |
| Visit No. | 1 | 2 | 3 | 4 | 5 |
| Visit Week | ≦-4W | 0W | 4W±5D | 8W±5D | 12W±5D |
| Informed consent | V |  |  |  |  |
| Basic information | V |  |  |  |  |
| Inclusion/exclusion criteria | V |  |  |  |  |
| Medical history ^1^ | V |  |  |  |  |
| Physical/Chest x-ray examination | V |  |  |  |  |
| Vital signs | V | V | V | V | V |
| Clinical laboratory^2^ | V |  | V | V | V |
| Pregnancy test^3^, HbA1c⁴ | V |  |  |  |  |
| ECG | V |  |  |  | V |
| Reid Colposcopic Index⁶ | V |  |  |  | V |
| Colposcopy with Biopsy⁵ | V |  |  |  | V |
| Pap Smear Test⁶ | V |  |  |  | V |
| HPV DNA Chip Test⁶ | V |  |  |  | V |
| HPV HC Ⅱ⁶ | V |  |  |  | V |
| NK Cell Activity⁶ |  | V | V | V | V |
| PBMCs ⁶ |  | V | V | V | V |
| Randomization |  | V |  |  |  |
| IP administration |  | V |  |  |  |
| Compliance |  |  | V |  |  |
| Adverse Events |  |  | V | V | V |
| Concomitant medication |  |  | V | V | V |

1. Medical history

1) History and Surgery: Based on the screening visit, related illnesses are within 2 years and other diseases are within 6 months.

2) Previous and concomitant medication: The medication that was administered within 4 weeks of the screening visit or currently administered.

2. Clinical laboratory

1) Hematological examination: Hct, Hb, WBC(diff. count), RBC, Platelet, ANC

2) Blood coagulation test: aPTT, PT

3) General chemical test: Glucose, AST, ALT, Alkaline phosphatase, Total Protein, Albumin Total Bilirubin, γ-GTP, Cholesterol (Total, LDL, HDL), Triglyceride, BUN, Creatinine, Uric acid, Sodium, Calcium, Potassium

4) Urinalysis: PH, Nitrite, Ketone, Specific gravity, Glucose, Protein, Urobilinogen, RBC

3. Pregnancy test: Urine hCG

4. Diabetes Screening: HbA1c

5. Primary efficacy variable: Colposcopic Biopsy

The test result within 4 weeks before acquisition of the consent is recognized as screening test.

(However, when using the results of other institutions, the results of biopsy should be secured so that the results can be confirmed at least.)

6. Secondary efficacy variable:

Cervical intraepithelial neoplasia evaluations

1) Reid Colposcopic Index

2) Pap Smear Test

3) HPV DNA Chip Test

4) HPV HC Ⅱ assay

Immunologic response evaluations

1) NK cell activity

2) MHC class II CD8, CD56 population

# Table of Contents

▣ Abbreviations and Definitions .............................................................................................................................................2

▣ Protocol Outline............................................................................................................................................3

1. Study Title and Phase...............................................................................................................................11

2. Study Site Name.................................................................................................................12

3. Principal Investigator, Sub investigator Name.........................................................13

4. Study Pharmacist Name........................................................................................................14

5. Sponsor Name......................................................................................................................................................15

6. Study Objectives and Background...............................................................................................................................16

7. Investigational Product Information.................................................................................................19

8. Indications..................................................................................................................................................................21

9. Inclusion/ Exclusion criteria, Sample size....................................................................................22

10. Study Duration...............................................................................................................................................25

11. Study Methodology............................................................................................................................................................26

12. Observation items, Clinical Examination Items and Observational Examination Methods..............................................................................................................................................................................28

13. Predictive Side Effects and Precautions for Use......................................................................................................38

14. Clinical Trial Suspension/ Dropout Criteria and Analysis Exclusion Criteria.........................................39

15. Evaluation Criteria, Evaluation Method, and Analysis Method....................................................................40

16. Evaluation Criteria of Safety Including Side Effects, Evaluation Method and Report Method......44

17. Clinical Trial Patient Consent Form................................................................................................................................47

18. Clinical Trial Patient Compensation Role......................................................................................................................48

19. Clinical Trial and Treatment Standard............................................................................................49

20. Measures to Protect the Safety of Patients.............................................................................................................50

21. What Else is Needed to Conduct Clinical Trials Safely and Scientifically.........................51

22. References.............................................................................................................................................................53

23. Attachment List............................................................................................................................................................55

Attachment 1. Sub-Investigators

Attachment 2. Study Pharmacist

Attachment 3. Monitor List

Attachment 4. Clinical Trial Patient Compensation Rule

**[1] Study Title and Phase**

A multi-center, randomized, double blind, placebo control, parallel design, phase 2b trial to evaluate the efficacy and safety of PGA (Poly-gamma-glutamic Acid) for the fertile women with Cervical Intraepithelial Neoplasia (CIN I)

**[2] Study Site Name**

### 2.1 Study Site Name

| Site Name | Address | Tel. |
| --- | --- | --- |
| The Catholic University Of Korea Seoul St. Mary's Hospital | 222 Banpo-dae ro, Seocho-gu, Seoul, Republic of Korea | 1588-1511 |
| Korea University Guro Hospital | 148 Gurodong ro, Guro-gu, Seoul, Republic of Korea | 02-2626-0114 |
| Cheil General Hospital and Women’s Healthcare Center | Seoae ro 1 Gil, Jung-gu, Seoul, Republic of Korea | 02-2000-7070 |
| Keimyung University Dongsan Medical Center | 56, Dalseong ro, Jung-gu, Daegu, Republic of Korea | 053-250-7114 |
| CHA Gangnam Medical Center, CHA University | 569 Nonhyon ro, Gangnam, Seoul, Republic of Korea | 02-3468-3000 |
| Gangseo MizMedi Hospital | 295 Gangseo ro, Gangseo-gu, Seoul, Republic of Korea | 1588-2701 |

**2.2 Participating organizations**

| Participating organizations | Address | Tel. |
| --- | --- | --- |
| Korea Research Institute of Bioscience & Biotechnology | 125 Gwahak-ro, Yuseong-gu, Daejeon, Republic of Korea | 042-860-4157 |

**[3] Principal Investigator, Sub investigator Name**

### 3.1 Coordinating Investigator

| Name | Site Name | Position | Tel. |
| --- | --- | --- | --- |
| Jae-Kwan Lee | Korea University Guro Hospital | Professor | 02-2626-3142 |

**3.2 Principal Investigator**

| Name | Site Name | Position | Tel. |
| --- | --- | --- | --- |
| Jong-Sup Park | The Catholic University Of Korea Seoul St. Mary's Hospital | Professor | 02-2258-2722 |
| Jae-Kwan Lee | Korea University Guro Hospital | Professor | 02-2626-3142 |
| Tae-Jin Kim | Cheil General Hospital and Women’s Healthcare Center | Professor | 02-2000-4713 |
| Chi-Heum Cho | Keimyung University Dongsan Medical Center | Professor | 053-250-7518 |
| Seok-Ju Seong | CHA Gangnam Medical Center, CHA University | Professor | 02-3468-3023 |
| Yong-Soo Park | Gangseo MizMedi Hospital | Chief of Dept. | 02-2007-1316 |

**3.3 Subinvestigator**

Attachment 1. Subinvestigator

## [4] Study Pharmacist Name

Attachment 2. Study Pharmacist

**[5] Sponsor Name**

### 5.1 Sponsor

| Name | Site Name | Position | Tel. |
| --- | --- | --- | --- |
| BioLeaders Corp | Moon-Hee Sung | Techno 8 ro 13, Yuseong-gu, Daejeon, Republic of Korea | 042-934-7671 |

**5.2 Monitor**

Attachment 3. Monitor

**[6] Study Objectives and Background**

### Study Objectives

This study is to compare the regression rate of Cervical Intraepithelial Neoplasia (CIN1) between the PGA group and the control group.

#### 6.1.1 Cervical Intraepithelial Neoplasia (CIN I)

Cervical Intraepithelial Neoplasia (CIN I) is a precancerous stage disease of cervical cancer. It is caused by HPV (human papillomavirus) infection, which is the cause of oncogenesis. According to histologic diagnosis, 1, 2, 3 I, II, III) to develop into cervical cancer. [1][2]

The number of patients with CIN I is estimated to reach 30 million people worldwide every year, and about 200,000 patients are born in Korea every year. In Korea, the number of patients with cervical cancer has decreased due to the early detection of the disease due to the cancer screening project due to the early detection of national cancer prevention policy. However, the number of CIN I, which is a precancerous stage, According to the announcement, the growth rate was 34% between 2007 and 2011.

A high-risk HPV-positive cervical cancer vaccine vaccine for the prevention of HPV infection was released, the most important factor in the development of cervical cancer. According to a survey by the Korean Obstetrics and Gynecology Society, the domestic vaccination rate in 2009 ~ 2010 was 3.3%. The vaccination rate has not been significantly increased due to the high price, the fact that vaccination against all types of HPV infection can’t be prevented, and the need for three in-hospital inoculations over 6 months.

It is also pointed out that the first sexual experience age is coming down at a steep pace. This is because the risks of cervical cancer can be greatly increased at the age of 20-30. Women with a history of cervical conization with CIN I are known to have significantly higher complications such as premature labor, preterm labor, premature rupture of membranes, and neonatal low birth weight compared to women who do not have cervical conization. [3] Even in the early stage of CIN I, it is necessary to check the progress of the disease through 4 ~ 6 months of follow-up visits for up to 3 years. Therefore, there is a need for psychological pain and medical burden for long-term cancer progression State.

Therefore, non-surgical methods should be considered as a priority for younger women who have been diagnosed with Cervical Intraepithelial Neoplasia (CIN I), and early-stage patients also require rapid healing through non-surgical methods. In order to prevent cervical cancer progression in women of childbearing age, it is an effective way to increase fertility rate and help safe birth, and in order to increase the cure rate of disease and to accelerate the healing period, BioLeaders Corp. γ-PGA immunotherapy product may contribute Expect.

### Background

Poly-gamma-glutamic acid (γ-PGA), a major component of the drug for clinical trials, is a sticky mucilage substance present in Chungkukjang, a traditional soybean fermented food that has been consumed for over 1,000 years. Poly (Gamma) glutamic acid is an anionic polymer substance in which glutamic acid is linked by a gamma peptide bond, and is a biopolymer material having biological stability produced by bacillus which is a GRAS (generally recognized as safe microorganism). In 2010, Ajinomoto Co., Japan presented to the US Food and Drug Administration (FDA) and designated GRASS as the FOOD item containing debittering agent of salt substitute product and KCl. Polygamma glutamic acid, which is similar to chungkukjang in Korea, is also present in Natto, a Japanese soybean fermented food. In Japan, various researches have been carried out for a long time and have already been used for food additives and food for specific health. This is the patient of much research.

BioLeaders has been associated with polygamma glutamic acid from the Korea Food and Drug Administration (MFDS) in 2003 as food raw materials (food 65421-326), 2006 natural food additives (No. 60), 2009 health functional foods, As the first in Korea.

BioLeaders has developed technologies for producing and purifying poly (γ-glutamic acid), which is superior to physicochemical and biological properties of Bacillus sp. Derived from natto in Japan, through domestic pure technology through research for about 10 years. This proved the function of various polygamma glutamic acid which was not known before. Among the newly identified functionalities, the immunostimulatory activity of high molecular weight poly (gamma glutamic acid) was confirmed by the animal efficacy tests as a therapeutic agent for diseases requiring immunotherapy.

There have been many reports that overcame cancer through Cheonggukjang. In fact, many people in Korea and Japan are taking Chungkukjang (Japanese Natto) as a part of civil immunotherapy. In fact, many successful cases of cancer survival in cancer patients such as colorectal cancer, kidney cancer, stomach cancer, and breast cancer have been reported. Therefore, many researches have been conducted on which components of Chungkookjang have anti-cancer effects. Genistein isoflavone family is known to have anticancer activity, which is known as chungkukjang. However, it is difficult to see enough genistein to be effective for anticancer effect. Actually, polygamma glutamic acid (γ-PGA) (Activity to induce the secretion of TNF-α and IFN-β, which are immuno-inducing cytokines, in macrophage treatment).

In collaboration with Korea Research Institute of Bioscience and Biotechnology, BioLeaders has demonstrated for the first time that high molecular weight polygamatoglutamic acid has an anti-cancer immunity enhancement function (J. Immunol. 2007, Cancer Immunol. Immunother. 2009)[4], And analyzed the products sold on the market. As a result, it was confirmed that about 150 mg of polygamma glutamic acid was contained in one pack of 50 g. Since polygamma glutamic acid can’t be expected to have the same efficacy due to different physico-chemical and biological characteristics (average molecular weight, viscosity, immune activity, etc.) depending on the kind of Bacillus subtilis fermenting the boiled soybeans, In order to develop novel pharmaceuticals containing polygamma glutamic acid as a main component, a highly purified purified poly (gamma glutamic acid) was obtained through the establishment of a production strain, a fermentation and purification process, and a non-clinical and clinical trial .

In this study, we performed randomized, double-blind, placebo-controlled clinical trials to evaluate the efficacy of immunosuppressive treatment of pure purified poly-gamma-glutamic acid in women with Cervical Intraepithelial Neoplasia (CIN I).

### 6.3 Outline of immunological enhancement function among biologic properties

The target disease, Cervical Intraepithelial Neoplasia (CIN I), has a characteristic that a certain percentage of the affected persons are naturally cured by the immune system in the body. Although the immune mechanisms involved in natural healing have not been clearly elucidated, it is known that innate immunity and viral antigen-specific cellular immunity are implicated in viral infection, It is known that the disease progresses due to immunity avoidance mechanism.

The mechanism of immunotherapy of polygamma glutamic acid, a major component of the drug to be developed so far, is largely classified into three categories: 1) activation of innate immunity, which is effective in preventing and treating early infection of virus, and 2) Inducing cell-mediated immune activation such as natural killer cells and cytotoxic T cells, and 3) mechanisms that inhibit the mechanism of avoiding the immune system related to inhibiting or curing disease progression.


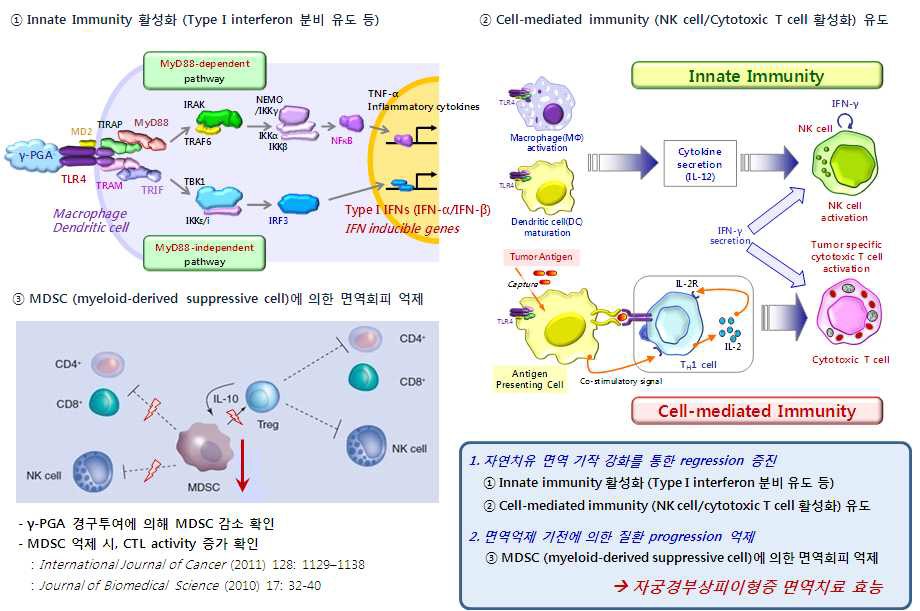


The immunostimulatory effect of oral administration of poly (gamma glutamic acid) is induced through the activation of macrophage cells and dendritic cells (DC), which are immune cells present in the small intestine mucosa, and ultimately, (J. Immunol. 2007), and the mechanism of anti-cancer immunity that activates cell-mediated immunity such as natural killer cells and cytotoxic T-cells is called macrophage cell ) And toll-like receptor 4 (TLR4), a receptor on the surface of dendritic cells (DC) (Cancer Immunol. Immunother. 2009).

### 6.4 Pre Study Results

1) Double-blind, randomized, double-blind, placebo-controlled clinical trials to assess the effect of 8 weeks of poly-gamma-glutamate on human immunity enhancement, The Catholic The Catholic University, Korea Seoul St Mary’s Hospital

- A total of 99 patients were enrolled in this study. The study included 33 patients with low-dose gamma glutamic acid (500 mg), 33 patients with high-dose gamma glutamic acid (1000 mg), and 33 patients with placebo. To compare the difference in the mean cytotoxicity of natural killer cells between the three groups, the mean changes in the cytotoxicity of natural killer cells at baseline and at each time point (4 weeks, 8 weeks) Using the Kruscal-Wallis test based on the normalization of the p values <0.05 was considered statistically significant.

- The mean changes in the cytotoxicity of natural killer cells at each time point (4 weeks, 8 weeks) compared to baseline were 0.5 ± 3.5% (increase rate: (P <0.05). There was a statistically significant difference between the two groups (4.5%, 4.3%, and 39.1%, respectively) 0.0174). Multiple comparisons showed that p-value = 0.0013 in the low-dose and high-dose groups, high-dose and placebo groups. As a result of multiple comparisons, there was a statistically significant difference in the change of cytotoxicity between the low dose group and the high dose group, the high dose group and the placebo group, and the cytotoxicity increase rate in the high dose group was higher than that in the low dose group and the placebo group.

- Phenotypic changes of peripheral blood mononuclear cells (MHC class II) by secondary efficacy: The CD8, CD4, CD8, CD14, and CD56 groups were compared between the three groups. , And the CD56 item was statistically significant (p-value = 0.0157, p-value = 0.0002) at the 4th and 8th weeks of the baseline, respectively. The results of multiple comparisons showed that the two items (CD8, CD56) showed different amounts of change in the low dose, high dose, high dose and placebo groups. In the safety evaluation, no patients were clinically withdrawn due to adverse events, and no serious adverse events were observed. There was no significant difference between the three groups in blood pressure and pulse, and no abnormality was found on laboratory examination

2) Randomized, placebo-controlled, single-dose/repeat-dose, phase-I clinical trials to assess safety, tolerability and pharmacokinetic characteristics after oral administration of poly-gamma glutamic acid in healthy male clinical trial subjects.

- When poly-gamma glutamic acid was orally administered to healthy male volunteers, it showed safety and tolerability up to 3.0gram/day.

## [7] Investigational Product Information

### 7.1 Investigational Product Information

#### Test: PGA

• Product Code : BLS-PGA

• Properties and Formulation : Brown glass bottle with clear viscous syrup

• Storage Method : Refrigerated storage (2 ℃ ~ 8 ℃), airtight container

• Expiration date : 24 months from date of manufacture

#### 7.1.2 Control: Placebo

• Properties and Formulation : Brown glass bottle with clear viscous syrup

• Storage Method : Refrigerated storage (2 ℃ ~ 8 ℃), airtight container

• Expiration date : 24 months from date of manufacture

### 7.2 Method of administration of Investigational Product

Administered once daily

### 7.3 Packaging and Labeling

#### 7.3.1 Packaging of Investigational Product

It is a bottle containing liquid formulation and packed in 100mL unit which can be taken once.

To avoid distinction between the test drug and the reference drug, the drug should be packed in a brown glass bottle of the same outline in the same volume to maintain double-blindness.

The payment package at the time of dispensing the clinical trial drug will include an extra five days for four weeks.

#### 7.3.2 Labeling of Investigational Product

1) Marked as “Investigational Product”

2) Investigational Product Code

3) Expiration date

4) Storage Method

5) Name and address of the person who has been approved for the Study plan

6) Marked as “Can’t be used for purposes other than study”

### 7.4 Management and Storage of Investigational Product

#### 7.4.1 Archive / Distribution / Management of Investigational Product

The Principal Investigator is responsible for ensuring that the Investigational Product received from the applicant is correctly received by the person in charge and kept in a safe place and that the Investigational Product is not damaged before the grant. The Investigational Product used in this study shall be controlled not to be used for purposes other than clinical studies. The management pharmacist must record the actual temperature using a graduated thermometer or thermocouple during storage, and record the measured temperature at least on the working day in the journal. If there is a temperature drift that is outside the acceptable range, you must contact your monitor. The temperature log should be kept in the laboratory laboratory file after the clinical trial is terminated.

The sponsor should check the quantity and storage status of the Investigational Product during the clinical trial and make sure that the clinical trial is conducted properly.

Each individual clinical trial subject package shall be delivered as provided by the sponsor without additional repackaging or labeling at the testing laboratory.

#### 7.4.2 Return of Investigational Product

The sponsor shall withdraw and discard the unused Investigational Product if the clinical trial is discontinued or terminated or if the clinical trial manager fails to conduct the test according to the plan. At this time, the Investigational Product Manager must return the unused Investigational Product to the sponsor after the consultation with the test manager and preserve the certificate. Returned Investigational Products are either disposed of or disposed of in accordance with the requirements of the applicant's company.

## [8] Indications

### Cervical Intraepithelial Neoplasia (CIN I)

According to the American Society of Colposcopy and Cervical Pathology (ASCCP) Consensus Guidelines in 2006, CIN Ⅰ diagnosed in women undergoes routine observation without treatment.[5]

## [9] Inclusion/ Exclusion Criteria, Sample Size

### Inclusion criteria

You may participate in this study only if all of the following criteria are met:

1. Fertile female aged between 20 and 49

2. Subjects who are diagnosed as Cervical intraepithelial neoplasia 1 (CIN1)

3. Subjects who are infected with HPV (Human Papilloma Virus) positive (+)

4. White Blood Cell Count(WBC) over 4 10^3^/ul, Hemoglobin above over 9.0g/dL Platelet over 150 10^3^/uL and ANC (Absolute Neutrophil Count) over 1,500 10^6^/L

5. AST (Aspartate Aminotransferase) no less than 4 times higher than normal ALT (Alanine Aminotransferase) no less than 4 times higher than normal

6. Normal for EKG (Electrocardiography) and no active disease detected trough chest X-ray

7. Be informed of the nature of the study and will give written informed consent

### Exclusion criteria

If any of the following criteria are met, you will not be able to participate in this study:

1. Malignant tumor in any organ other than cervical intraepithelial neoplasia

2. Active liver disease, immune disorder and severe renal failure

3. Leukemia, collagenosis, sclerosis, autoimmune disease, clinically significant allergic disease (mild allergic symptom not required medicine excluded)

4. Diagnosed diabetes

5. Taking any of followings affecting immunological reaction within 7 days (Glucocorticoid, vitamins, health food and oriental medicine etc)

6. Pregnancy and breastfeeding

7. Registered in other clinical trials

8. Patients whom the investigator considers inappropriate to participate in the study

### 9.3 Sample size

#### 9.3.1 Sample size

##### - Total 200 patients (PGA Group 100 patients, Placebo Group 100patients)

Based on published literature, we assumed that 31% of the cases of CIN 1 would resolve spontaneously, and a clinically significant difference was estimated at 20%. 80 patients per group.

| Group | Dose | Patient | Expected response Rate | Expected response patient | Duration of treatment/Observation |
| --- | --- | --- | --- | --- | --- |
| Placebo | - | 80 | 31% | 24.8 | administered once daily for 4 weeks /  observation for 8 weeks |
| PGA | 1,500mg/day | 80 | 51% | 40.8 |  |
| Total | - | 160 |  |  |  |

Cervical intraepithelial neoplasia (CIN I) can act as a precursor to invasive cervical cancer and is the most common significant gynecologic disease in women of reproductive age. study, we investigated clinical efficacy and immune response of oral administration of γ-PGA from women with cervical intraepithelial neoplasia 1 (CIN 1) in phase 2b study

H_0_ : p_1_≦p2 vs. H_1_ : p_1_>p_2_

p_1_ : Response rate of treatment, p_2_: Response rate of Control

Expected response rate: PGA Group: 81%, Control Group: 51%

significance level : 0.05, statistical power : 80%, one-sided test

The Sample size is total 160 patients, 80 patients per group when the Single stage method.

If 20% drop out is considered, the sample is total 200 patients, 100 patients per group

The sample size is according to the Two stage optimal design (Jung(2008)) Method is shown in the following table.

[Jung(2008) Two-stage]

| Stage | Patient | Difference between two groups (Treatment patient-Control patient) | 제 significance level, statistical power |
| --- | --- | --- | --- |
| 1  2 | 43  80(43+37) | +2  +11 | 0.461, 0.800 |

###### 9.3.2 Basis of calculation

The prevalence of Cervical intraepithelial neoplasia (CIN I) in the Korean population is very limited, and there is little clinical study on the efficacy of the treatment [6]. In this study, To investigate the effect of glutamic acid, we refer to the results of research abroad.

Most of the studies on natural cure rates for Cervical intraepithelial neoplasia (CIN I) were done for more than 6 months. The natural cure rates at 3 months as in the present study were compared with those of CIN I and II (Barnett et al. As a result of the study, about 31% in the placebo group and about 33% in the photodynamic therapy (PDT).[8] A 15-week study of Trimble (2005) with CIN II and III showed a natural cure rate of 28%. [9]

In a study by Bansal et al. (2008), who conducted a cohort study, the study included 1001 CIN I patients who were followed up for at least 12 months to determine the natural cure rate at the 6-month follow-up, approximately 49% of the patients were reported to return to normal. [10]

In a phase 2 clinical trial (Hefler et al., 2010) to confirm the effect of topical infusion of progesterone in a similar clinical trial phase, the natural cure rate of the control group without special treatment was 38.8% of 96 patients. [11]

Based on the results of the above studies, it can be summarized that most of the natural cure rates are within 3 months and the additional effect after 3 months is about 7.8% ~ 18.0%. The natural cure rate at 3 months was set at 31.0%.

This study was established as an exploratory study with phase 2 clinical studies and it was concluded that at least 20% of the effects of high natural healing rates should be clarified to enable large scale clinical studies. Was not worth it.

## [10] Study Duration

Approximately 48 months from IRB approval date

**[11] Study Methodology**

The purpose of this study was to investigate the effect of oral administration of poly-gamma glutamic acid (γ-PGA) on the Cervical Intraepithelial Neoplasia (CIN I) Double-blind, placebo-controlled study designed to identify immunomodulatory effects by assessing changes in immunological parameters.

In this study, a total of 80 patients (100 patients in the case of dropout) were participated in the study. Participation in the study was conducted in two stages as proposed by Jung (2008). In the first stage, 43 subjects were recruited in each group, and the main effect variable, healing, was checked. The difference in the number of subjects in the clinical trial for healing was less than 2 (the number of subjects who were treated for healing of poly-gamma glutamic acid - The number of subjects in clinical trials), it is judged that there is no effect of poly-gamma glutamic acid on cervical epithelial dysplasia and the study is terminated.

If there is more than one difference in step 1, we will consider a two-step study design that will enter step 2 and add an additional 37 patients per group. If the difference in the number of subjects in the clinical trial between group 1 and group 2 of 80 patients in each group is greater than 11 patients, the effect of γ-PGA will be confirmed. Otherwise, γ-PGA. It will be judged that it is not effective in healing the injured Cervical Intraepithelial Neoplasia (CIN I).

### 11.1 administration dosage

###### 11.1.1 Basis for administration dosage

The results of immune histochemical staining for 5 days after 5 consecutive days of clinical phase 1 study showed that the natural killer cell activity was generally higher in the dose group than in the placebo group, (1.5 g / day) showed higher natural killer cell activity than the high dose group (3.0 g / day).

There was no case of serious adverse reaction / abnormal drug response in the preceding test, and administration was decided at a dose of 1.5 g / day, which indicates a functionally high natural killer cell activity as an immunotherapeutic agent in a dose of 1.5 g / day and 3.0 g / day Respectively.

###### 11.1.2 administration dosage

PGA Group: Poly-gamma-glutamic Acid 1,500mg / 100mL

Control Group: Placebo / 100mL.

### 11.2 Method of administration dosage

100 mL of poly-gamma-glutamic acid or placebo is taken orally once a day.

Because food interferes with the absorption of this drug, take it before bedtime for adequate absorption. If you forgot to take it on the prescribed day, take it only on the same day and do not take more than once on the same day.

### 11.3 Duration of administration dosage

###### 11.3.1 Basis for duration of administration dosage

Based on the results of the Phase I and Phase II clinical studies, the clinical efficacy of polygamma glutamic acid was determined to be 4 weeks and the same period as the non - rodent repeated dose toxicity data.

###### 11.3.2 Duration of administration dosage

4 weeks

### Concomitant medication

###### Combination drugs

In the case of other diseases or adverse reactions, information such as drug name, purpose of administration, dosage, duration of administration, etc. should be recorded in the case record for all drugs used in combination.

###### Prohibited drugs

The drugs to be prohibited during the clinical trial period are as follows. When the use of medication for concomitant medication is required, the clinical trial subject should stop the clinical trial

1) Steroids: Betametasone, Dexametasone, Prednisolone, Hydrocortisone, etc., except hen except when applied locally to skin or eyes.

2) Immunomodulators: growth hormone, EPO (Erythropoietine), albumin preparations, transfusion of whole blood and leukocyte components, etc

3) Others: Supplements and vitamin preparations other than clinical trial drugs. Chinese medicine

## [12] Observation Items · Clinical Examination Items and Observational Examination Methods

### 12.1. Observation and clinical examination items

Observation and examination items and schedule are as follows.

| Visit Type | Screening | Baseline &  Treatment | Observation | Observation | Closing |
| --- | --- | --- | --- | --- | --- |
| Visit No. | 1 | 2 | 3 | 4 | 5 |
| Visit Week | ≦-4W | 0W | 4W±5D | 8W±5D | 12W±5D |
| Informed consent | V |  |  |  |  |
| Basic information | V |  |  |  |  |
| Inclusion/exclusion criteria | V |  |  |  |  |
| Medical history ^1^ | V |  |  |  |  |
| Physical/Chest x-ray examination | V |  |  |  |  |
| Vital signs | V | V | V | V | V |
| Clinical laboratory^2^ | V |  | V | V | V |
| Pregnancy test^3^, HbA1c⁴ | V |  |  |  |  |
| ECG | V |  |  |  | V |
| Reid Colposcopic Index⁶ | V |  |  |  | V |
| Colposcopy with Biopsy⁵ | V |  |  |  | V |
| Pap Smear Test⁶ | V |  |  |  | V |
| HPV DNA Chip Test⁶ | V |  |  |  | V |
| HPV HC Ⅱ⁶ | V |  |  |  | V |
| NK Cell Activity⁶ |  | V | V | V | V |
| PBMCs ⁶ |  | V | V | V | V |
| Randomization |  | V |  |  |  |
| IP administration |  | V |  |  |  |
| Compliance |  |  | V |  |  |
| Adverse Events |  |  | V | V | V |
| Concomitant medication |  |  | V | V | V |

1. Medical history

1) History and Surgery: Based on the screening visit, related illnesses are within 2 years and other diseases are within 6 months.

2) Previous and concomitant medication: The medication that was administered within 4 weeks of the screening visit or currently administered.

2. Clinical laboratory

1) Hematological examination: Hct, Hb, WBC(diff. count), RBC, Platelet, ANC

2) Blood coagulation test: aPTT, PT

3) General chemical test: Glucose, AST, ALT, Alkaline phosphatase, Total Protein, Albumin Total Bilirubin, γ-GTP, Cholesterol (Total, LDL, HDL), Triglyceride, BUN, Creatinine, Uric acid, Sodium, Calcium, Potassium

4) Urinalysis: PH, Nitrite, Ketone, Specific gravity, Glucose, Protein, Urobilinogen, RBC

3. Pregnancy test: Urine hCG

4. Diabetes Screening: HbA1c

5. Primary efficacy variable: Colposcopic Biopsy

The test result within 4 weeks before acquisition of the consent is recognized as screening test.

(However, when using the results of other institutions, the results of biopsy should be secured so that the results can be confirmed at least.)

6. Secondary efficacy variable:

Cervical intraepithelial neoplasia evaluations

1) Reid Colposcopic Index

2) Pap Smear Test

3) HPV DNA Chip Test

4) HPV HC Ⅱ assay

Immunologic response evaluations

1) NK cell activity

2) MHC class II CD8, CD56 population

### 12.2. Observation test method

#### 12.2.1 Acquired a consent form for patients

Prior to conducting this trial, the investigator will explain to the subject the following information: After confirming that the patient is well understood, the consent of the applicant to participate in the clinical trial according to his / her own free will is handwritten by the signer and received as a signed document. Also enter the date on which the consent was obtained in the case record.

1) Purpose and method of the clinical trial.

2) Expected adverse reactions.

3) The fact that the patient is not disadvantaged without agreeing to take the clinical trial.

4) Even if the clinical trial patient agrees to take the test, he / she can withdraw it at any time

5) Other matters necessary for the protection of human rights of clinical trial patients and understanding of investigational products

#### 12.2.2 Demographic, medical history, medication survey and physical examination

Before entering the clinical trial, examine the demographic and medical history of the patients to be examined, check the charts, and check the following items and record them in the case record.

1) Demographic Survey: Clinical trial patients Initial, gender, age

2) Investigation of medical history: Presence of present illness, presence of past drug acute reaction, existence of immune system disease, presence of special constitution, etc.

3) Drug investigations: Drugs taken within the past 4 weeks (including those currently being taken)
4) Previous history: Other disease history (within 2 years ~ currently treated)

5) Physical examination: Examination of current physical condition.

#### 12.2.3 Vital signs

Measure your blood pressure and pulse rate according to the progress schedule in Section 12.1. Blood pressure should be measured after resting for 5 minutes in a steady state. In a sitting position, remove all your strength from your body, relax your breathing, and measure with a standard sphygmomanometer.

#### 12.2.4 Clinical test

12.1 Perform the inspection according to the progress schedule.

Sampling and recording: Blood collection should be performed aseptically according to the schedule of the examination. Blood samples should be inspected at the clinic laboratory, which is certified by the quality control system, according to the commercial hematology and blood chemistry method. Record the results of the test on the case record and record the researcher's comments on the abnormal values

Inspection items:

1) Hematological examination: Hct, Hb, WBC(diff. count), RBC, Platelet, ANC

2) Blood coagulation test: aPTT, PT

3) General chemical test: Glucose, AST, ALT, Alkaline phosphatase, Total Protein, Albumin Total Bilirubin, γ-GTP, Cholesterol (Total, LDL, HDL), Triglyceride, BUN, Creatinine, Uric acid, Sodium, Calcium, Potassium

4) Urinalysis: PH, Nitrite, Ketone, Specific gravity, Glucose, Protein, Urobilinogen, RBC

5) Pregnancy test: Urine hCG

6) Diabetes Screening: HbA1c

#### 12.2.5 ECG

Perform a 12-lead ECG according to the timeline of 12.1. In addition to basic recording, record the items such as ventricular rate (beats / min), PR interval (msec), QRS (msec) and QT / QTc (msec) in the CRF.

#### Chest X-ray examination

##### Chest X-ray is taken to check if there is any significant abnormality in the findings such as chest lung disease or lesion.

**12.2.7 Cervical intraepithelial neoplasia (CIN 1)** **diagnostic test** [12-15]

Carry out the inspection according to the progress schedule of 12.1.

1) Colposcopic Biopsy

The results of vaginal histologic biopsy were classified as Normal, CIN I, CIN II, CIN III, and SCC according to the World Health Organization (WHO) CIN system. The chronic inflammatory findings are classified as normal. CIN I was selected as the result of a biopsy of the vaginal epithelium.

< Standardized method for Colposcopy >

1. During the colposcopy, the patient is placed in the lithotomy position and the anomalous part of the perineum is identified. The cervix is inserted and the cervix is observed through the colposcope when the cervix is exposed.

2. The vaginal secretions that interfere with the visual field are carefully removed and first applied normal saline solution to observe the overall appearance, leukoplakia and vascular status at 5 to 10 magnification. At this time, the green filter filters the red color of the blood vessels and makes them appear black so that the blood vessels can be observed better.

3. After examination with saline solution, the change of epithelium by acetic acid solution will be observed. Apply cervical portion with a 3 to 5% acetic acid solution using a wetted cotton swab or cotton swab. When the first acetic acid solution is applied, it is observed after about 30 seconds to 1 minute. When the acetic acid solution is applied once more, the reaction of the acetic acid solution started by the first application is increased.

4. After application of the acetic acid solution, the junction of squamous cells is observed and the presence of erosion, aceto-white areas and blood vessels are observed. Generally, acetic acid white epithelium can be clearly observed at a magnification of 5 to 16 times.

5. After the application of acetic acid solution is completed, 25 ~ 50% of Lugol iodine solution should be applied and all areas should be observed again. Normal squamous epithelium contains glycogen, which appears to be dark brown iodine positive by iodine solution, and lesions do not respond to iodine.

6. The biopsy specimen is a biopsy of two of the most severe abnormalities. However, if the lesion is small and the biopsy is difficult in two cases, only one lesion is found. And biopsy forceps are used to collect the tissue. Biopsy of more sites is not allowed because it may be the purpose of removing lesions.

7.. Bleeding from the biopsy site usually stops on its own, but you can use a concentrated Monsel solution to stop bleeding immediately or prevent delayed bleeding.

8. Reid's index and photographs are taken and proved by records.

The vaginal specimens were taken from the cervix and the cervical vertebrae, and the lesions were included. The acetic acid solution and the iodine solution were used. It is necessary to be able to give a Reid's index score objectively by looking at this photograph, and if the lesion or cervical portion does not come in one picture, it is divided into two pieces.

<Abnormal colposcopy opinion >

| White epithelium: It differs from leukoplakia in that it shows white lesion with definite boundary after acetic acid drip. It is a phenomenon caused by increased density of nucleus due to proliferation of epithelial cells. It is a common finding in the tumor (CIN) | 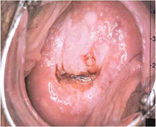 |
| --- | --- |
| Punctation: A localized epithelium of capillaries with a clear borderline, which is common in cervical intraepithelial neoplasia (CIN). | 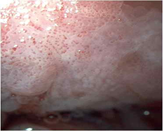 |
| Mosaic (mosaic): A mosaic pattern is formed by forming a basket-shaped vascular network between the surface epithelium. The mild dysplasia is irregular as the lesion progresses. It is mainly associated with CIN I and above lesions | 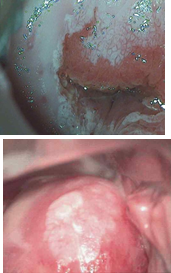 |
| Leukoplakia: A white lesion that is caused by hyperkeratosis of the topical epithelium and is flat, irregardless of acetic acid spot. Clinically, there is no significant difference in the presence of squamous epithelium. However, if the lesion is localized, the precancerous lesion should be suspected. | 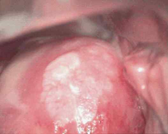 |
| Atypical vessels: The shape, diameter, circumference, and distance between the vessels of the peripheral capillary vessels are irregularly different from those of normal squamous epithelium. In most cases, there is a risk of invasive cancer. | 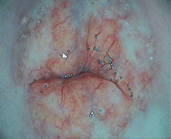 |

<Production method of Tissue specimen for microscopic observation>

1. Tissue obtained from a biopsy of the vaginal epithelium is fixed with formaldehyde (HCHO).

2. The fixed tissue is embedded with paraffin.

3. The paraffin block is sectioned into thin tissue sections in micrometers for microscopic observation.

4. Tissue sections are stained with Hematoxylin-Eosin and observed under a microscope.

< CIN, cervical intraepithelial neoplasia diagnosis>

| CIN I | mild dysplasia: The lower third of the epithelium is replaced by immature undifferentiated cells |
| --- | --- |
| CIN II | moderate dysplasia: Replaced by immature undifferentiated cells up to mid-third of epithelial layer |
| CIN III | severe dyaplasia: Up to 1/3 of the epithelial layer was contained but mature cells were still present on the surface |
|  | Carcinoma in situ: An undifferentiated undifferentiated abnormal cell replaces an epithelial cell layer |


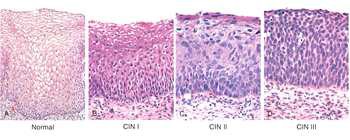


- - - 1. Reid Colposcopic Index

In order to predict the severity of CIN lesion, various colposcopy findings were classified and scored according to their characteristics to create objective, accurate, and reproducible indices. The grading system was classified as margin of the lesion, color of the acetowhitening, type of vascular pattern, and iodine staining reaction [16].

| **Reid Colposcopic Index** | | | | | | |
| --- | --- | --- | --- | --- | --- | --- |
| Colposcopic Sign | Zero Point (0) | Check | One Point (1) | Check | Two Points (2) | Check |
| **Margin** | Exophytic condylomas;areas showing a micropapillary contour.  Lesions with distinct edges. Feeatheard,  scalloped |  | Lesions with a regular(circular) shape, showing smooth, straight edges. |  | Rolled, peeling edges. Any internal demarcation  between areas of differing colposcopic appearance. |  |
| **Color** | Shiny, snow-white color.  Areas of faint (semi-transparent)  whitening. |  | Intermediate shade (shiny, but gray-white). |  | Dull reflectance with oyster-white color. |  |
| **Vessels** | Fine-caliber vessels, poorly formed patterns. |  | No surface vessels. |  | Definite, coarse punctation or mosaic. |  |
| **Iodine** | Any lesion staining  mahogany brown; mustard-yellow staining by a minor lesion (by first three criteria). |  | Partial iodine staining (mottled pattern). |  | Mustard-yellow staining of a significant lesion (an acetowhite area scoring 3 or more points by the first three criteria). |  |

The score is scored from 0 to 8, and grading according to the score is judged as follows.

0-2: Likely to be CIN I

3-4: Overlapping lesion - likely to be CIN I or CIN II

5-8: Likely to be CIN II

2) Pap smear (Liqid Based cytology)

Cervical cytology is done by placing the long central part of the brush (broom) in the intrauterine landscape and rotating it 2 ~ 3 times in the same direction, then putting the brush into the container containing the preservative liquid and closing the lid.

The test results are read as Negative, ASC-US, ASC-H, LSIL, HSIL, Invasive cancer (Cervical carcinoma).

3) HPV DNA Chip test

Cervical cytology is performed using a specimen collected from uterine and ectopic cytobrush. In order to confirm the correlation between HPV infection type and virus type, visit 1, visit 5. HPV types are classified as positive and negative. HPV types 16, 18, 31, 33, 35, 39, 45, 51 and 52 are recorded when they are positive.

4) HPV Hybrid Capture Ⅱ assay (viral load)

HPV viral load was measured by Hybrid capture II system. The solution in the collection kit was centrifuged, the confluent solution was separated, mixed with the denaturation reagent, and incubated at 65 ° C for 45 min. Then, 13 kinds of high- and incubated for 60 min at 60 ° C to capture the RNA / DNA hybrid monoclonal antibody in a test tube attached to the surface of the antibody. Then, a secondary immunoblot and a chemiluminescent Lumiphos530 and incubated for 15 minutes. The light emitted by decomposition of substrate by alkaline phosphatase is measured using a luminometer. The light units of the sample were positive control group containing 10 pg / mL of DNA of HPV-16, -18 and relative light unit RLU (relative light unit), It is judged to be HPV negative. This is done to investigate the correlation between HPV virus and tumor in the epithelium.

The biopsy is performed in the pathology room where the quality control of each institution is performed and it is carried out according to the standardized method of tissue examination. Pap smear test and HPV test should be performed in the laboratories of designated clinical trial institutes considering that there may be differences in results depending on the analysis method or procedures between hospitals.

The sample transportation management of each institution will be collected and transported by the person in charge of BioLeaders Co., Ltd. to the research facility according to the guidelines for the management of the sample for clinical examination.

#### 12.2.8 Reviewing the inclusion / exclusion criteria

The histopathologic results of Biopsy were checked for CIN I, HPV DNA , and histopathologic examination. The results of the background screening, history, medical examination, physical examination, ECG, vital signs, as a result of the test, we evaluated whether the pharyngo virus was positive for the inclusion and exclusion criteria.

#### 12.2.9 Evaluation of immunological index

Serologic and cellular immune responses of serologic and cellular immune systems in HPV infection are important for loss of cervical dysplasia, a disease associated with infected conditions and infection. By inducing a cell-mediated immune response through a person already infected with HPV, the progression of HPV infection and related lesions can be modulated or inhibited.

Perform blood collection for the immunoassay evaluation according to the progress schedule of Section 12.1.

<Labeling>

Each plasma tube is attached using a pre-provided label. The necessary information on the label should be described using oil-based inks, and the required record item on the labeling shall be the number of the patient to be examined, the initials, and the date of blood collection.

<Time of examination>

All plasma samples collected in this study should be tested in batch.

<Inspection method>

Pretreatment and quantification of the samples should be performed according to a pre-validated method, and the reliability of the method should be guaranteed by the analytical institute and the sponsor of the clinical trial.

<Inspection institution and inspectors>

The analysis is carried out at the relevant departments (Diagnostic Tests Department) of the Clinical Trial Execution Institution that has received the certificate from the Korean Association of Clinical Pathology Quality Management or at a research establishment with this level of test at a level comparable to that of the test institute. (SOP) of the relevant organization. It shall be documented so that the inspection of the personal data of the inspector and the raw data of the test results is possible.

1. Measurement of NK cell activity

Recent changes in NK activity measurement using CD107a marker ss an ex vivo test, mononuclear cells are first isolated from peripheral blood drawn from patients. To determine the activity of NK (natural killer) cells, K562 cells are used as target cells of natural killer cells. The target cells are inoculated in a FACS tube at a concentration of 4 × 10 5 cells / mL at 100 μL, and peripheral blood mononuclear cells are added to the FACS tube at a ratio of 10: 1 to the target cells. Natural killer cell activity is calculated by measuring the protein labeling on the surface of natural killer cells when K562 cells, the target cell, are killed.

1. Measurement of MHC class II CD8, CD56 population

Peripheral blood mononuclear cells (PBMNCs) are separated by density gradient method using a boiling solution of ficoll-hypaque after peripheral blood of the subject is collected. Blood samples were superimposed on a Histopaque (specific gravity 1.077 g / ml sterile-filtered, Sigma) solution using a SEPMATE tube (stemcell, 15450) and centrifuged at 1200 × g for 10 min. The upper layer was separated and washed with PBS + 2% To remove mononuclear leukocytes.

(PBMNCs) of the subjects were treated with FACS buffer (0.1% Bovine serum albumin, 0.01% sodium chloride) to confirm the change of peripheral blood mononuclear cell immunity phenotype through a flow cytometer (BD-true counter) (CD3-Percp-eFluor 710, CD8-FITC, CD56-PE, CD14-APC-eFluor 780, and CD19-APC-eFluor 780, CD107a-AlexaFluor 647) is added and left on ice for 30 minutes.

Fluorescent stained cells were washed twice with FACS buffer, fixed with 2% parafomaldehyde in phosphate buffer, and analyzed for cell fluorescence using a flow cytometer (Gallios, Beckman, USA).

The sample transportation management of each institution will be collected and transported by the person in charge of BioLeaders Co., Ltd. to the research facility according to the guidelines for the management of the sample for clinical examination.

#### 12.2.10 Randomization assignment

Clinical trial patients are assigned sequential accession numbers by researchers (regardless of the order of Screening No.). In random assignment code, random assignment code is randomly generated by a statistian or a randomization assignee in a way that is unique to computer generation. In this study, block randomization method of appropriate block size is used and assigned to each institution. The randomization assignments must be performed in sequence, and the random assignment code can’t be reassigned to another trial patient even after the patient has already been assigned once, even if the subject withdraws consent.

#### 12.2.11 Prescription and grant Investigational Product

The Principal Investigator of the site is responsible for the management of the Investigational Product and the Investigational Product management should keep and manage the Investigational Product used for the clinical trial by the pharmacist's management pharmacist for the purposes other than the clinical trial.

The sponsor should check the quantity and storage status of the Investigational Product during the clinical trial and make sure that the clinical trial is conducted properly. The Principal Investigator is responsible for ensuring that the Investigational Product delivered from the sponsor is received correctly by the management pharmacist, is stored in a safe place under the recommended storage conditions, and is responsible for preserving the drug for trial testing before delivery.

Each individual clinical trial patient package shall be delivered as provided by the sponsor without additional repackaging or labeling at the site.

#### 12.2.12 Returning Investigational Product and assessing compliance

The sponsor shall withdraw and discard the Investigational Product if the clinical trial is discontinued or terminated, or if the clinical trial representative fails to perform the test according to the protocol. After consultation with the investigator, the clinical trial drug manager shall return the returned clinical trial drug to the sponsor after the use of the unused Investigational Product and the clinical trial patient, and preserve the return certificate. The returned investigational Product shall be disposed of or disposed of in accordance with the client's internal regulations.

###### Confirm medication compliance of Investigational Produc

The study patients will return the empty containers with the Investigational Product taken at Visit 2 (0weeks), the unused medicines, and the recorded medicines daily record, and the research coordinator will return the quantity of medicines prescribed at Visit 2 Determine the quantity of Investigational Product taken by the subject of the clinical trial and record the adherence compliance on the case report sheet as follows. The medicinal compliance of the Investigational Product is considered to be "compliant" if it is> 80%.

Compliance (%) = Number of Investigational Products Taken / Number of Investigational Products Taken X 100

- - 1. **Assessing Adverse Events**

Adverse events are closely monitored from the time of administration to the end of the clinical trial. In the event of an adverse event, the date and time of the adverse event, the extent and consequences of the adverse reaction, the measures taken in relation to the Investigational Product, the causal relationship with the Investigational Product, the name of the suspected drug other than the test drug, and details of the contents are recorded. After the visit, the adverse reaction should be immediately reported to the researcher, the clinical trial patient should be trained so that the patient can be admitted to the clinic, and the researcher should be contacted. The evaluation, recording and treatment of detailed adverse events is in accordance with section 16.3.

#### Concomitant medication investigation

During the clinical trial period, drugs other than Investigational Product should not be administered. Therefore, sufficient training for clinical trial subjects should be provided. It is also necessary to educate the researcher to contact the researcher if it is necessary to take medication.

If the drug administered without judgment is expected to have an impact on the efficacy and safety of the trial, the patient will be disqualified. All medications and causes of administration should be investigated through written interviews and charts of subjects and recorded in the case record. The following drugs are prohibited in this trial.

1) Steroids: Betametasone, Dexametasone, Prednisolone, Hydrocortisone, etc., except when except when applied locally to skin or eyes.

2) Immunomodulators: growth hormone, EPO (Erythropoietine), albumin preparations, transfusion of whole blood and leukocyte components, etc

3) Others: Supplements and vitamin preparations other than clinical trial drugs. Chinese medicine

#### 12.2.15 Check Cervical intraepithelial neoplasia (CIN 1) progress

Observe for 8 weeks after completion of the Investigational Product from visit 2 (0 weeks) to visit 3 (4 weeks ± 5 days). At the final visit of the Visit 5 (12 weeks ± 5 days), an effective evaluation test of the cervical dysplasia including Colposcopic Biopsy is performed. If the progression is on the Colposcopic Biopsy, the standardized treatment method and terminates the clinical trial of the patient.

### 12.3. Visit schedule overview

All visits to the patients must be scheduled on a pre-scheduled basis and strictly follow the Visit Window of each visit.

The Visit Window of each visit is within 4 weeks of visit 1, 0 is weekly visit 2, 4 weeks ± 5 days of visit 3, 8 weeks ± 5 days of visit 4, and 12 weeks ± 5 days of visit 5 The total duration of the patients is 12 week.

#### Visit 1(Within -4 weeks, Screening)

If you want to participate in the clinical trial after receiving explanation of this study, write down the written consent of the patient to be tested and perform the screening procedure and examination described below. Those with clinically significant anomalies are excluded from clinical trials.

1) Written consent of clinical trial patients

2) Demographic information

3) Examination of medical history (within 2 years of related disease, within 6 months of other diseases), taking medication survey (within 4 weeks)

4) signs of vitality, physical examination, physical measurement (weight, height)

5) Laboratory test, pregnancy test

6) Electrocardiography

7) Chest X-ray examination

8) Colposcopy (Reid's Index, Biopsy)

The test result within 4 weeks before acquisition of the consent is recognized as screening test.

(However, when using the results of other institutions, the results of biopsy should be secured so that the results can be confirmed at least.)

9) Pap smear test, HPV DNA test, Hybrid Capture II assay

10) Conformity Assessment of Clinical Test Subject (Selection / Exclusion Criteria)

###### Subject Screening/Enrollment Log

###### Record the list of clinical trial patients who signed the clinical trial patients' consent form in the selection log. If the trial box is not eligible or can’t participate in this test for any other reason, the reason should be recorded.

#### Visit 2 (0 week Baseline & Treatment )

1) Final evaluation of applicability of clinical trial patients

2) Measurement of signs of vitality

3) Confirmation of Concomitant medication change

4) Assignment number

5) Prescribing and issuing of Investigational Product

6) NK cell activity and immune phenotype test

###### Review of inclusion / exclusion criteria

The inclusion / exclusion criterion is reviewed through the vital signs and clinical test results of the patient of the clinical trial, and it is decided whether or not to be selected by the suitability evaluation of the final test patient.

#### Visit 3(4 weeks ±5 days, Observation)

1) Measurement of signs of vitality

2) Confirmation of Concomitant medication change

3) Investigational Product Return and assessment of medication compliance

4) Check adverse events

5) NK cell activity and immune phenotype test

6) Laboratory inspection

#### Visit 4(8 weeks ±5 days, Observation)

1) Measurement of signs of vitality

2) Confirmation of Concomitant medication change

3) Check adverse events

4) NK cell activity and immune phenotype test

5) Laboratory inspection

#### 12.3.5 Visit 5(12 weeks ±5 days, Closing)

1) Measurement of signs of vitality

2) Confirmation of Concomitant medication change

3) Check adverse events

4) Laboratory inspection

5) ECG examination

6) Colposcopic Biopsy test

7) Colposcopy (Reid's Index), Pap smear test

8) HPV DNA test, Hybrid Capture II test

9) NK cell activity, immune phenotype test

It is necessary to confirm the cure or progress of the Cervical intraepithelial neoplasia and to give sufficient medical explanation according to the condition of the Cervical intraepithelial neoplasia of the clinical trial patient and to carry out the follow-up observation or standardized treatment.

## [13] Predictive Side Effects and Precautions for Use

### 13.1 Predictive side effects

No significant adverse events were observed in previous clinical trials of poly gamma glutamic acid, so there is no anticipated side effect, but poly gamma glutamic acid may be a viscous syrup formulation that may cause discomfort when taken according to the individual's taste is predicted.

### 13.2 Precautions for use

Careful administration: No specific side effects have been reported so far, so follow the prescribed dosing instructions. If the patient complains of discomfort or abnormal symptoms during the clinical trial, report to the researcher and the researcher will check whether the adverse reaction has occurred and take appropriate action.

**[14] Clinical Trial Suspension / Dropout Criteria and Analysis Exclusion Criteria**

### 14.1 Clinical Trial suspension ·dropout criteria

The followings are the items which can be the clinical trial stoppage and dropout of the clinical trial patients participating in the clinical trial

1) An acute response (allergic, hypersensitive reaction) to Investigational Product

2) If the investigator determines that continuing participation is not appropriate due to the occurrence of a "serious adverse reaction".

3) In the event of a breach of the selection / exclusion criteria or a violation of a significant clinical study protocol.

4) Patients will be able to withdraw from the trial at their own request.

5) In case of no observation due to absence of clinical test patients.

6) If the other tester determines that the investigator should be stopped.

If the patient is withdrawn from the study, the Investigational Product should be discontinued and the date, reason, and findings should be recorded in the case record, along with all the results of the trial obtained to the point of withdrawal. If a clinical trial is excluded from clinical trials as a result of clinically significant laboratory performance, follow-up should be conducted until the event or laboratory performance is resolved or a reasonable period is determined.

**14.2 Analysis exclusion criteria**

In case of a violation of a critical clinical trial plan, the patient should be excluded from the PP analysis.

1) If the patient's compliance with the Investigational Product is less than 80%

2) In case of violation of selection criteria and exclusion criteria during the test

3) In combination with drugs that may affect the efficacy evaluation

4) When the validity evaluation item inspection (visit 1) is not carried out

5) If you visit due to visiting date

Other violations of the clinical trial protocol, which are considered to have no effect on the interpretation of the study results, must be clearly stated in terms of the violation or delay and the reason and the researcher, sponsor, monitor personnel and statisticians and to include them in the PP analysis.

## [15] Evaluation Criteria, Evaluation Method, and Analysis Method (Statistical Analysis Method)

### 15.1 Effect criteria

The primary and secondary efficacy parameters for evaluating clinical trial results are as follows and the validity will be analyzed for each of the outcome variables.

### 15.1.1 Primary efficacy evaluation variable

The definition of comparative healing between test group and control group at 12 weeks after drug administration compared to Baseline (within -4 weeks) before drug administration means the case of normalization at the level of CIN I.

### 15.1.2 Secondary Validity Variables

Compared to baseline (within -4 weeks) before drug administration Reid Colposcopic Index at 12 weeks after drug administration

Compared to baseline (within -4 weeks) before drug administration Pap smear test results at 12 weeks after drug administration

Comparison of HPV DNA chip test results at 12 weeks after drug administration compared to Baseline (within -4 weeks) before drug administration HPV HC Ⅱ assay results at 12 weeks after drug administration compared to baseline (within -4 weeks)

Comparison of results of NK cell activity at 4, 8 and 12 weeks after drug administration compared to baseline (0 week) before drug administration

(MHC class II) CD8, CD56 population at 4 weeks, 8 weeks, and 12 weeks after administration of drug compared to baseline (0 week) before drug administration.

### Evaluation Method

#### 15.2.1 The primary efficacy end point

Compared to baseline (less than -4 weeks) before drug administration, test group vs. control group at 12 weeks after drug administration

Colposcopic biopsy was performed at visit 1 and visit 5 as a result of colposcopic biopsy, which is a Cervical intraepithelial neoplasia, after drug administration compared to baseline before drug administration. The test group and the control group are compared for the measured healing after 3 months compared with baseline

The stage of change in the Cervical intraepithelial neoplasia of this study is defined as follows.

- Regression: if healed in CIN I

→ Normal in CIN I

- Persistence: if CIN I step is the same without change

→ CIN I to CIN I

- Progression: In case of progressing more than 1 step in CIN I

→ CIN I, CIN II, CIN III, SCC (Squamous cell carcinoma).

#### 15.2.2 The secondary efficacy end point

1) Comparison of Reid Colposcopic Index after administration of drug

Reid Colposcopic Index is a grading system that classifies and scales various findings of CIN lesions according to their characteristics. The score is scored from 0 to 8, and the grading according to the score is determined as follows.

0-2: Likely to be CIN I

3-4: Overlapping lesion - likely to be CIN I or CIN II

5-8: Likely to be CIN II

2) Comparison of Pap smear test results after administration

Cervical cytology was performed using the two-slide technique, using a cytobrush of the uterus and an ectopic cytobrush. The cervical cytology was based on the 2001 Cervical Cytologic Diagnoses. The readings are read in six steps: negative, ASC-US, ASC-H, LSIL, HSIL, and invasive cancer (cervical carcinoma).

3) Comparison of HPV DNA chip test results after administration of drug

Cervical cytology is performed using a specimen collected from uterine and ectopic cytobrush. To confirm the correlation between tumor epithelial cells and HPV infection and virus type. HPV types 16 and 18, 31, 33, 35, 39, 45, 51 and 52 are recorded for positive and negative HPV.

4) Comparison of HPV HC Ⅱ assay results after drug administration

Cervical cytology is performed using a specimen collected from uterine and ectopic cytobrush. It is an examination that is performed as one of the methods to predict the severity of cervical intraepithelial neoplasia. The test result is classified as positive or negative based on the light unit of the sample. If the result is positive, it is recorded as Low, Intermediate, High.

5) Comparison of results of NK cell activity after administration of drug compared to drug administration

Blood is collected from visit 2 and visit 3, visit 4, visit 5, and it is evaluated according to the measurement method of 12.2.8 1).

Recent changes in NK activity measurement using CD107a marker As an ex vivo test, mononuclear cells are first isolated from peripheral blood drawn from subjects. K562 cells are used as target cells of natural killer cells to measure the activity of NK (natural killer) cells. The target cells are inoculated in a FACS tube at a concentration of 4 × 10 5 cells / mL at 100 μL, and peripheral blood mononuclear cells are added to the FACS tube at a ratio of 10: 1 to the target cells. Natural killer cell activity is calculated by measuring the protein labeling on the surface of autologous killing cells when K562 cells, the target cell, are killed

6) Phenotypic changes of peripheral blood mononuclear cells (MHC class II) after drug administration compared with the results of CD8, CD56 population

Blood was collected from Visit 2 and Visit 3, Visit 4, and Visit 5, and evaluated according to the measurement method of 12.2.8 2) Peripheral blood mononuclear cells (PBMNCs) are separated by density gradient method using a boiling solution of ficoll-hypaque after peripheral blood of the subject is collected. Blood samples were superimposed on a Histopaque (specific gravity 1.077 g / ml sterile-filtered, Sigma) solution using a SEPMATE tube (stemcell, 15450) and centrifuged at 1200 xg for 10 min. The upper layer was separated and washed with PBS + 2% To separate mononuclear cells. (PBMNCs) of the subjects were treated with FACS buffer (0.1% Bovine serum albumin, 0.01% sodium chloride) to confirm the change of peripheral blood mononuclear cell immunity phenotype through a flow cytometer (BD-true counter) (CD3-Percp-eFluor 710, CD8-FITC, CD56-PE, CD14-APC-eFluor 780, and CD19-APC-eFluor 780, CD107a-AlexaFluor 647) is added and left on ice for 30 minutes. Fluorescent stained cells were washed twice with FACS buffer, fixed with phosphate buffer containing 2% parafomaldehyde, and then the fluorescence of the cells was measured and analyzed using a flow cytometer (Gallios, Beckman, USA).

###### 15.3 Statistical analysis method

**15.3.1 General principles of analysis**

The purpose of this study was to investigate the effect of oral administration of polygamma glutamic acid (γ-PGA) on the cervical intraepithelial dysplasia (CIN) Phase II clinical studies to confirm. All data obtained in this study are summed up as mean ± standard deviation for continuous data and categorical data for the number of subjects (fraction), and various summary statistics are presented when necessary. If it is necessary to provide an interval estimate, a 95% confidence interval shall be provided.

In the statistical analysis of this data, the first validity criterion, the healing rate, is to be carried out by a one-sided test at a significance level of 5% based on the method of Jung (2008) in the appropriate validity analysis group defined in 15.2. In the analysis other than the primary efficacy evaluation item, the two-sided test is performed at the significance level of 5%. In addition to the efficacy evaluation methods and safety evaluation methods described in 15.3.4 and 15.3.6, when prognostic factors contributing to the outcome variables such as age, perinatal risk, baseline laboratory values, (Eg, logistic model, ANOCOVA, generalized linear model, etc.).

###### 15.3.2 Definition of analytical group

The analytical groups needed to assess efficacy and safety in this trial were performed in both the ITT and PP assay groups as defined below for efficacy assessments in accordance with national and international standards, Define the analysis and evaluate it as a reference.

Efficacy analysis group:

- Intent-to-Treatment (ITT) group: Defined as all subjects assigned to randomization after agreement to participate in the study. But patients who did not take the study drug at least once in the ITT group will be excluded from the analysis group.

- PP (Per Protocol) group: defined as the total subjects who completed the clinical study according to the study plan without any violation of the important research plan presented in 14.2 until the end of the study after the consent to participate in the study.

- In the efficacy evaluation, all the analyzes are performed in the ITT group and the PP group, and the final evaluation is decided according to the results of the ITT group analysis. If the results of the ITT group and the PP group are different, an additional analysis will be conducted exploring why the two analyzes are different.

Safety evaluation analysis group:

After agreeing to participate in the study, they will be defined as the whole subjects who were randomized and who took the research drug at least once.

#### 15.3.3 Missing value processing method

In the analysis of validation variables, LOCF (Last Observed Carried Forward) method is applied to replace missing values, and safety evaluation parameters are analyzed without substitution if there is a mistake.

#### 15.3.4 Analysis of primary efficacy endpoints

In this study, the primary efficacy endpoint was to determine the cure rate as normal at the third month of drug administration of CIN I at baseline as described in 15.2.1 and compare the healing rates between the test and control groups.

The evaluation of the healing rate between the groups was based on the method proposed by Jung (2008). As described above, when 43 patients were selected in each group (intermediate analysis) in the first step, When the difference in number is less than 2 (test group-control group), it is judged that there is no effect of poly gamma glutamic acid, and the present study is terminated. If there were two or more difference in level 1, 37 patients were recruited in each group. In the case of more than 11 patients who were judged to be cured by 80 patients per group, it is judged that there is an effect of poly gamma glutamic acid. If it is less than 11, it is concluded that the effect of poly-gamma-glutamic acid is not ultimately.

If the final number of subjects is different in each analysis group after the end of step 2.


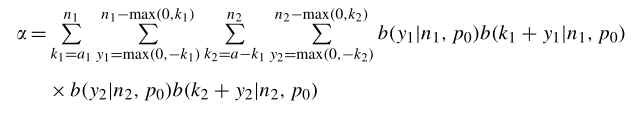


here,

n1, n2: Number of subjects per group in steps 1 and 2

a1, a: The first and final stages to determine whether to adopt the null hypothesis. The difference between the groups of healed subjects y1, y2, x1, x2:

b (y|n, p): probability distribution of binomial distribution

We will present the p-value based on the significance level formula.

In addition, the number of children, number of sex partners, and whether smokers are covariates during baseline diagnosis will be analyzed by logistic regression analysis using "exploratory analysis"

### 15.3.5 Analysis of secondary primary efficacy endpoints

Secondary efficacy variable 1: Reid Colposcopic Index

0-2 Score: Likely to be CIN I

3-4 Score: Overlapping lesion - likely to be CIN I or CIN II

5-8 Score: Likely to be CIN II

The comparison between the baseline scores and the difference of the scores after 12 weeks will be confirmed through the t-test or the Wilcoxon rank sum test. In addition, considering the multinomial distribution of the grading system (0-2, 3-4, 5-8 points), the cumulative or ordinal logistic model of the changes in the grading system after 12 weeks compared to baseline.

Secondary efficacy variable 2: Pap Smear Test

The reading standard is based **on the 2001 Bethesda System for Reporting Cervical Cytologic Diagnoses**. The results of the study were divided into 6 stages: Negative, ASC-US, ASC-H, LSIL, HSIL, and Invasive cancer (Cervical carcinoma). Considering the multinomial distribution, And the cumulative or ordinal logistic model is used to analyze changes in the classification stage 3 months after the baseline.

Secondary efficacy variable 3: HPV DNA Chip Test

The HPV DNA Chip Test is a test to determine the type of virus infection. It is classified as positive or negative according to the test result. GEE (Generalized Estimating Equation) or Generalized Linear Mixed Model is considered to determine time-dependent changes, group-to-group differences, and time-to-group interactions for baseline versus measured clearance.

Secondary efficacy variable 4: HPV HC Ⅱ assay

GEE (Generalized Estimating Equation) or Generalized Linear Mixed Model is considered to determine time-dependent changes, group-to-group differences, and time-to-group interactions for baseline versus measured clearance.

Secondary efficacy variable 5: NK cell activity

To compare the results of NK cell activity measured at 4 weeks, 8 weeks, and 12 weeks versus baseline (0 week), the mean difference between the baseline and 12-week NK cell levels was calculated as t - Black or Wilcoxon rank sum test.

Secondary efficacy variable 6: MHC class II CD8, CD56 population

GEE (Generalized Estimating Equation) or Generalized Linear Mixed Model is considered to determine time-dependent changes, group-to-group differences, and time-to-group interactions for baseline versus measured clearance.

#### 15.3.6 Analysis on safety evaluation variables

All safety data, including all adverse events collected from patients, clinical laboratory test results, 12-lead ECG, and vital signs (blood pressure and pulse rate) are performed

#### 1) Adverse events

Summarize the adverse events observed following administration of the investigational product. The number of cases and the number of patients who experienced an adverse reaction, abnormal drug response, SAE, death, adverse events n that caused discontinuation of clinical trial, and / or "other significant adverse events (OAE) do. The number of subjects who received each of the abnormal responses was also summarized by body-organ system (SOC), recommended language, and maximum severity for each treatment group. In addition to the summary statistics, the incidence or incidence of adverse events in the group are analyzed using Chi-squared test, Fisher's Exact test, or Poisson test.

#### 2) Vital signs and laboratory tests

The results were analyzed by Chi-squared test, Fisher's Exact test, or Poisson test for each group of 12-lead ECG abnormalities at each time point. A summary statistic is presented and, if necessary, GLM or GLMM methods are used to perform group comparisons.

## [16] Evaluation Criteria of Safety Including Side Effects, Evaluation Method and Reporting Method

### 16.1 Adverse events evaluation

The adverse reaction is closely observed from the time of administration of the drug, and it is confirmed through consultation with the examiner during the visit and after consultation with the investigator. The date and date of the adverse reaction, the degree and the result of the adverse reaction, Details of the causal relationship with the measure and the Investigational Product, the name of the suspected drug other than the Investigational Product, the treatment and the treatment of the adverse reaction. In the event of an adverse reaction, the researcher should be informed immediately, and the clinical trial subject should be trained so that the patient can receive medical treatment. The evaluation, recording and treatment of detailed adverse events is in accordance with section 16.3.

### 16.2. Predicted adverse events

No significant adverse events were observed in the preceding clinical trial.

### 16.3 Evaluation methods and evaluation criteria for adverse reactions such as self-elevation symptoms

Adverse events are any undesirable anatomy, physiological or metabolic lesions that occur during the test, regardless of whether they are associated with an Investigational Product, or changes in somatic symptoms, signs, or clinical signs. These include worsening of existing conditions, comorbidities, and drug interactions.

In this clinical study, when the clinical results of the visit 2 were beyond the normal range of the experimental test, the vital sign measurement, and the electrocardiogram, the investigator judged the clinical significance of the result, Adverse events were reported with findings. It is the responsibility of the person responsible for the clinical trial to document any adverse events that occur during clinical trials. Adverse events should be recorded in medical diagnostic terms and, if this is not possible, be observed by the investigator or the person responsible for the clinical trial, or recorded the terms of the symptoms and signs reported by the subject.

The case record records the symptoms and signs of the adverse reaction, duration (start and end date), severity, causal relationship with the drug for the clinical trial, measures taken in relation to the adverse reaction.

1) Severity of adverse events

The severity of the adverse events is assessed in five grades of Grade 1 to 5 according to CTCAE v4.0, but Grade 4 or 5 is considered a serious adverse event. The following is a generic example of CTCAE v4.0.

| 1 = Mild | Asymptomatic or mild symptoms: Only routine or diagnostic observation is possible. Therapeutic intervention is not required |
| --- | --- |
| 2 = Moderate | Minimal, local, or non-invasive treatment is needed: routine activities such as meal preparation and shopping |
| 3 = Severe or medically significant but not immediately life-threatening) | Severe, medically significant, or not immediately life threatening: limited self-care activities such as bathing, dressing, meals, toiletries, etc., without hospitalization or extension of length of stay |
| 4 = Life-threatening consequences) | Immediate treatment is required |
| 5 = Death related to AE | Death-related adverse events |

2) Causality with Investigational Product

When an adverse reaction occurs, the investigator classifies the investigator as follows and records his or her opinion on whether or not it is related to the investigational product.

| Evaluation step | Evaluation standard |
| --- | --- |
| Certain | If the relationship between drug administration and adverse events is reasonable  If the adverse event is not explained by other medicines, chemicals or accompanying diseases  Clinically appropriate response at discontinuation  Re-administration (if possible only) is pharmacologically or phenomenologically critical |
| Probable/Likely | If the temporal relationship between drug administration and adverse events is reasonable  If the adverse event does not appear to be due to other medicines, chemicals or accompanying diseases  Clinically appropriate response at discontinuation  If there is no re-admission information |
| Possible | If the temporal relationship between drug administration and adverse events is reasonable  If the adverse event is also explained as being caused by other medicines, chemicals or accompanying diseases  Insufficient or unclear information about discontinuation |
| Unlikely | In the case of transient cases in which there is no likely causal relationship between drug administration and adverse event manifestations  If the adverse reaction is due to other medicines, chemicals or potential disease. |
| None | If an adverse reaction occurs without drug administration  If the adverse events that occurred before drug administration did not deteriorate after administration |
| Conditional/Unclassified | If you need more material or are reviewing additional material for proper evaluation |
| Inaccessible /Unclassifiable | Incomplete or conflicting information and can’t be determined or supplemented or confirmed |

3) Actions taken in relation to adverse events

| 0 = No action taken |
| --- |
| 1 = Study drug dosage adjusted / temporarily interrupted |
| 2 = Study drug permanently discontinued due to this adverse event |
| 3 = Concomitant medication taken |
| 4 = Non-drug therapy given |
| 5 = Hospitalization / prolonged hospitalization |

### 16.4 Evaluation methods and evaluation criteria for serious adverse events

Serious AEs (ADRs) refer to any of the following adverse events or adverse drug reactions occurring at any dose of the Investigational Product used in the test:

1) In case of death or danger to life

2) If you need to stay in the hospital or need to extend the length of stay

3) Permanent or significant failure or degradation of function

4) If the fetus has malformed or abnormalities

In addition, the following items are not regarded as serious adverse reaction.

1) If you do not need hospitalization due to voluntary will

2) If you are admitted to the hospital due to surgeries, tests, etc.

All adverse events should be recorded in the supporting documentation and case record, and the same adverse reaction terms should be used. If a "serious adverse event" occurs during the study period, report it to the Institutional Review Board to decide whether to continue or discontinue the study.

If serious adverse drug reactions occur during the course of a clinical trial, the test taker will cease part or all of the trials for the clinical trial drug and report it immediately to the trial investigator and the trial sponsor. The investigator should report to the clinical trial committee and test sponsor by telephone or facsimile within 24 hours of occurrence or at least the next day. In addition, within 7 days after the occurrence of a serious adverse event, additional reports should be included with the documentation in details. The sponsor should promptly report any adverse events that have not been reported to other relevant researchers or jury members in accordance with the drug clinical trial management standards.

1. Rapid Reporting: In the event of a serious adverse event occurring during the course of a clinical trial, regardless of whether it is related to the Investigational Product, the investigator or the investigator should contact the Clinical Trials Commission within 24 hours (Or the person in charge) of the clinical trial entrusted institution entrusted by BioLeaders or CRO.

* Monitor Person (or Person in Charge): Attachment 3. Note for Monitor

2. Detailed Reporting: Additional reporting should be made to the documentation within 5 days after the occurrence of the serious adverse event.

3. Tracking adverse events

The investigator or the person in charge should observe the clinical symptoms of the adverse reaction until the symptom subsides and the abnormal clinical test returns to baseline or a satisfactory explanation for the observed change is obtained. In addition, the progress of the adverse reaction should be reported to the person in charge.

## [17] Clinical Trial Patient Consent Form

The consent of the clinical trial patient must be made in accordance with the Helsinki Declaration of Ethical Principles and KGCP standards and the test director must obtain the approval of the clinical trial committee for the protocol form and the protocol to be used prior to commencing the clinical trial. Inform the clinical trial subject or the representative of the clinical trial subject with sufficient information about the contents of the trial and the effect of the trial, the adverse event, and obtain written consent.

Respect the self-determination of the patient of the clinical trial, and conduct interviews, explanations and agreements in a separate space.

If the patient of the trial and the representative of the patient are unable to read the documented information, the enrollee must attend the entire process of obtaining consent.

Each signature must be dated directly by the signer, the consent form and the test subject manual shall be retained by the examiner and the examiner, and a copy of the signed agreement shall be provided to the subject of the clinical trial or to the subject of the clinical trial patient.

Also, make sure to keep a record on the basis document for the process of obtaining consent.

It is the investigator's responsibility to obtain consent from the clinical trial patient or the trial patient 's representative prior to the initiation of any act or treatment of the trial that is not the patient's routine care.

attachment 5 Clinical Trial Patient Manual and Consent

**[18] Clinical Trial Patient Compensation Rule**

**Covenant on Victims Compensation**

1. Compensation shall be made for any damage to the clinical trial subject during the period of the trial, if any of the following items are met. However, reimbursement is limited to cases where there is no evidence that there is no causal relationship between the injury to the trial patient and the Investigational Product administered to the trial or trial.

- damage caused by the use of the Investigational Product in accordance with the agreed protocol

- the damage caused as a result of the diagnostic process performed in accordance with the agreed protocol

- damage resulting from legally required treatment or diagnostic methods as a result of unexpected side effects

2. The following cases shall not be compensated.

- damage due to side effects caused by drugs not provided in this trial

- Injury due to disease or congenital disease inherent in the clinical trial patient regardless of this Investigational Product

- damage caused by the researcher failing to comply with agreed protocol

- damage caused by carelessness of the patient

3. The person responsible for the clinical trial should do his best to comply with the related laws and regulations and the mutually agreed clinical trial plan so that the patient of the clinical trial will not receive any disadvantage by the clinical trial.

Despite these efforts, however, during the clinical trial, if the clinical trial patients suffer from injury (injury or side effects) by the Investigational Product provided in this study, reasonable treatment fees or compensation will be paid according to the following compensation evaluation criteria.

<Reward evaluation standard>

The cost of treatment or compensation shall be the appropriate amount to treat or compensate for the nature, severity, duration, and continuity of the damage, and shall be equal to the amount normally paid for similar damages in Korean courts. If there is a disagreement between the test patient and the trial coordinator regarding the level of compensation, consult with the experts selected by agreement between the two parties, and consult with the adviser when consulting.

I hereby pledge that I will be liable for the damage suffered by the clinical trial patient in this clinical trial based on the above contents.

. .

Responsibility: BioLeaders (Sign)

**[19] Clinical Trial and Treatment Standard**

Because Cervical intraepithelial neoplasia is important for further observation, follow the ASCCP 2006 recommendation for periodic screening.

If the lesion progresses, cone resection is the usual treatment for cervical intraepithelial neoplasia, and it is recommended that periodic screening be done to prevent and early detection of cervical cancer progression

As a general treatment, local destruction therapy crystallizes the intracellular fluid to cause cell necrosis and destroys the cervical epithelium surface layer. Anesthesia-free, safe and effective cryosurgery, laser cervical irradiation of the cervical epithelium, laser therapy that can be used even when the pathologic changes are very extensive or spread to the vaginal area. In the method of heating for 20 seconds, the treatment effect is cold coagulation similar to cryotherapy.

As a resection, wire loops are used to remove the entire strain. (LEEP), cold (cold) or cold (cold) procedures that can be performed easily and quickly because of the advantages of being able to perform the diagnosis and treatment with one procedure using the removed tissue. The most common recurrence rate among cervical intraepithelial neoplasia (CIN) conization and conization, which can be performed for diagnostic and therapeutic purposes, is the resection using knife, CO 2 laser or electrosurgical instrument. The lower method is hysterectomy.

Follow-up tests using cell-alone or colposcopy are performed every year if the test results are negative for a certain period of time. It is reported that complications due to therapeutic intervention may occur after phantom diathermy or cone resection, bleeding, infection, adherence to the surface, cervical incompetence, spontaneous abortion, premature labor, and low birth weight.

Provide guidance to other patients who have been withdrawn from clinical trials to receive other appropriate treatment. Clinical trial patients who have completed clinical trials should receive diagnostic and appropriate treatment according to the instructions of the clinical investigator.

**[20] Measures to Protect the Safety of Patients**

1) Before participating in the clinical trials, strictly evaluate whether the subjects are appropriate for the study. Particularly in the case of women of childbearing age, the Urine hCG test should not be omitted. Select the person whose pregnancy response is negative by the test, and keep the contraception during the trial.

2) Any adverse event will be reported to the researcher about the symptoms, signs, and diseases that occur after the next day of Baseline, ie after taking the Investigational Product.

3) Clinical trial is conducted according to CLINICAL STUDY PROTOCOL, and evaluation and evaluation of adverse reactions and the occurrence of harmful drug cases are carried out through inspection and screening at the time of visit.

## [21] What Else is Needed to Conduct Clinical Trials Safely and Scientifically

. **21.1 Data Quality Assurance and Data Management Plans**

It will be responsible for the data management of this test at the specialized institution responsible for the culling and statistics. We will review the written case records and input the data into the database by logical check using the computer program to confirm and guarantee the accuracy, completeness, consistency and logical data, and solve all problems found in the data management process. I will be in close contact with my researchers.

**21.2 Clinical Trial Management Standard (KGCP)**

In carrying out this test, we will conduct research under ethical and scientific considerations in accordance with the spirit of the KGCP and the Helsinki Declaration. When inviting the clinical trial patients to the clinical trial, they should obtain a consent by interviewing them after fully explaining the benefits of the clinical trials in the independent space and the possible risks.

### 21.3 Direct Access to evidence

To ensure the safety of this trial patients and to obtain accurate, complete, and reliable data, the investigator should keep the clinical records, clinical records, and medical records of the clinical trial patient as documentation. The researcher should allow direct access to the test materials at the request of the sponsor, the Ministry Food and Drug Safety, and the Clinical Trials Panel.

### Monitoring

The monitoring staff appointed by BioLeaders Co., Ltd. visits the testing institute at appropriate intervals at regular intervals for monitoring the progress of the test, and performs a task of comparing case records and source documents. The researcher must agree to cooperate with the test monitor personnel or the mandate of this service to access the storage place of the Investigational Product and the documents related to the test. Monitoring is carried out basically in the vulture and BioLeaders Co., Ltd.

### Case Report Form

The source documents of the patients of the clinical trial means the patient records of the doctors who are kept in the testing laboratory. Most source documents are charts from the hospital or physician, and in this case all information collected and recorded in the patient's electronic case record should match the chart.

When the data to be recorded is obtained, a case record should be written as soon as possible. It is the researcher's responsibility to record, review, and sign case records. After writing a case record, the researcher signs each case record to ensure that the information recorded in the case record is true. In all cases, the investigator has final responsibility for the accuracy and reliability of all clinical and laboratory values recorded in the case record.

### 21.6 Confidentiality of research data and archival provisions for clinical trials

It is the responsibility of the Research Staff to ensure that protocol patients have received the Center’s Notice of Privacy Practices. If the patient has not already done so, personnel of the relevant participating Center must try to obtain acknowledgment before the patient participates in this study.

The Center’s Privacy Office may allow the use and disclosure of protected health information pursuant to a completed and signed Research Authorization form. The use and disclosure of protected health information will be limited to the individuals described in the Research Authorization form. A Research Authorization form must be completed by the Principal Investigator and approved by the IRB and Privacy Board.

### 21.7 Protocol Change

If the clinical trial protocol is approved by the clinical trial committee and changes to the clinical trial protocol are made due to a wider range of test procedures, increased risk, changes in patient selection criteria, or additional safety information, it must be approved. When revising the clinical trial protocol, the date of revision, the reason for revision, and the details of revision should be recorded and retained.

Approval is not required for minor changes that do not affect the progress of the test, such as changing words, changing the monitor, or modifying plans for statistical analysis to correct misspellings or to make them more explicit.

### 21.8 Protocol Violation

Except for medical emergencies, it is not permitted to violate the final signed clinical trial protocol without prior consent between the researcher and BioLeaders.

If the violation of the clinical trial protocol is a serious violation that requires the patient to continue the trial, the researcher or monitor should record the information in a protocol deviation form and send it to BioLeaders, it is required to obtain the approval of BioLeaders Co., Ltd. for the continuation of the test of the patient to be tested.

## [22] References

[1] Laimins LA. Humanpapillomaviruses target differentiating epitheria for virion production and malignant conversion. Semin Virol 1996;7:305-13

[2] Depuydt CE, Vereecken AJ, Salembier GM et al.Thin-layer liquid-based cervical cytology and PCR for detecting and typing humanpapillomavirus DNA in Flemish woman. Br J Cancer 2003;88:560-6

[3] Van Hentenryck M, Noel JC, Simon P. Obstetric and neonatal outcome after surgical treatment of cervical dysplasia. Eur. J. Obstet. Gynecol. Reprod. Biol. (2012) 162: 16-20

[4] Lee TY, Kim YH, Yoon SW, Choi JC, Yang JM, Kim CJ, Schiller JT, Sung MH, Poo H. Oral administration of poly-gamma-glutamate induces TLR4- and dendritic cell-dependent antitumor effect.

Cancer Immunol. Immunother. (2009) 58: 1781-1794

[5] Tomas C. Wright Jr, MD;L. Stewart Massad, MD;Charles J.Dunton, MD;Ma가 Spitzer, MD;EdwardJ.Wilkinson,MD; Diane Solomon,MD;for the 2006 American Society for Colposcopy and Cervical Pathology-sponsored Consensus Conference

[6] Saw HS, Lee JK, et al. Natural History of Low-Grade Squamous Intraepithelial Lesion (Journal of Lower Genital Tract Disease, Volume5, Number3,2001,153-158)

[7] Jung SH. Randomized phase Ⅱ trials with a prospective control. Statist. Med. (2008) 27: 568-583

[8] Barnett et al. A randomised, double‐blind, placebo‐controlled trial of photodynamic therapy using 5‐ aminolaevulinic acid for the treatment of cervical intraepithelial neoplasia. International Journal of Cancer 2003;103:829-832.

[9] Trimble et al. Spontaneous Regression of High-Grade Cervical Dysplasia: Effects of Human Papillomavirus Type and HLA Phenotype. Clin Cancer Res July 1, 2005 11; 4717.1

[10] Bansal N, Wright JD, Cohen CJ, Herzog TJ. Natural History of Established Low Grade Cervical Intr aepitherial (CIN1) Lesions. Anticancer Res. (2008) 28 : 1763-1766

[11] Hefler L, Grimm C, Tempfer C, Reinthaller A. Treatment with Vaginal Progesterone in Women with Low-grade Cervical Dysplasia. A Phase Ⅱ Trial. Anticancer Res. (2010) 30 : 1257-1262

[12] Bentley J. Colposcopic management of abnormal cervical cytology and histology. J Obstet Gynaecol Can. 2012;34(12):1188-202.

[13] Teresa M. Darragh, et al. The Lower Anogenital Squamous Terminology Standardization Project for HPV-associated Lesions: Background and Consensus Recommendations From the College of American Pathologists and the American Society for Colposcopy and Cervical Pathology. Int J Gynecol Pathol 2013;32(1).

[14] ANCUŢA BOICEA, et al. Correlations between colposcopy and histologic results from colposcopically directed biopsy in cervical precancerous lesions. Rom J Morphol Embryol 2012;53(3):735–741.

[15] Nu´ria-Laia Rodrı´guez-Mias, et al. Current Situation: Lower Genital Tract Pathology and Colposcopy Training in Spanish Gynecology and Obstetrics Residents. Journal of Lower Genital Tract Disease, 2013;17(1):12-16

[16] Reid R, Scalzi P. Genital warts and cervical cancer: VII. An improved colposcopic index for differentiating benign papillomaviral infections from high-grade cervical intraepithelial neoplasia. Am. J.

Obstet. Gynecol. (1985) 153: 611-618

[17] 대한 산부인과학회와 국립암센터 2001, 조기검진 권고안_학회지

[18] Jae-Sun Park, M.D., Hee-sug Ryu., M.D., Suk-jun Chang,.M.D. The association of the cervical intraepitherial neoplasia and human papillomavirus viral load. Kor.Obstet. Gynecol. Vol. 48 No.13 Dec. 2005

[19] 진소영, 박상모, 김미선, 진윤미, 김동원, 이동화 Diagnostic Accuracy of Cervicovaginal Cytology in the Detection of Squamous Epithelial Lesions of the Uterine Cervix; Cytologic/Histologic Correlation of 481

Cases. (2008) 111-118

[20] Hilleman MR. Overview of vaccinology with special reference to papillomavirus vaccines. J Clin Virol 2000;19:79-90

[21] Stanley MA Immunobiology of papillomavirus infections. J Rreprod Immunol 2001;52:45-59

[22] Abe K, Ito Y, Ohmachi T, Asada Y. Purification and properties of two isozymes of γ

-glutamyltranspeptidase from Bacillus subtilis TAM-4. Biosci. Biotechnol. Biochem. (1997) 61: 1621-1625

[23] Birrer GA, Cromwick AM, Gross RA. Poly-γ-glutamic acid formation by Bacillus licheniformis 9945A : physiological and biochemical studies. Int. J. Biol. Macromol. (1994) 16: 265-275

[24] Discacciati MG, de Souza CA, d'Otavianno MG, Angelo-Andrade LA, Westin MC, Rabelo-Santos SH, Zeferino LC. Outcome of expectant management of cervical intraepithelial neoplasia grade 2 in women followed for 12 months. Eur. J. Obstet. Genecol. Reprod. Biol. (2011) 155: 204-208

[25] Eom SY et al. Effect of Keumsa Sangwhang (Phellinus linteus) mushroom extracts on the natural killer cell activity in human. Korean J. Food Sci. Technol. (2006) 38(5): 717–719

[26] Perez-Camero G, Congregado F, Bou JJ, Munoz-Guerra S. Biosynthesis and ultrasonic degradation of bacterial poly-γ-glutamic acid. Biotechnol. Bioeng. (1999) 63: 110-115

[27] Goto A, Kunioka M. Biosynthesis and hydrolysis of poly-γ-glutamic acid from Bacillus subtilis IFO3335.

Biosci. Biotechnol. Biochem. (1992) 56: 1031-1035

[28] Hezayen FF, Rehm BH, A Tindall BJ, Steinbuchel A. Transfer of Natrialba asiatica B1T to Natrialba taiwanensis sp. nov., a novel extremely halophilic, aerobic, non-pigmented member of the Archaea from Egypt that produces extracellular poly(glutamic acid). Int. J. Syst. Evol. Microbiol. (2001) 51: 1133-1142

[29] Hiroyuki T et al. A calcium supplement containing poly-γ-glutamic acid increase human calcium absorption. Nippon Nogeikagaku Kaishi. (2003) 77(5): 504–507

[30] Ing-Lung Shih, Yi-Tsong Van. The production of poly-γ-glutamic acid from microorganisms and its vari-ous applications. Bioresource Technol. (2001) 79: 207-225

[31] Ito Y, Tanaka T, Ohmachi T, Asada Y. Glutamic acid independent production of poly-γ-glutamic acid by Bacillus subtilis TAM-4. Biosci. Biotechnol. Biochem. (1996) 60: 1239-1242

[32] Kim TW, Lee TY, Bae HC, Hahm JH, Kim YH, Park C, Kang TH, Kim CJ, Sung MH, Poo H. Oral administration of high molecular mass poly-gamma-glutamate induces NK cell-mediated antitumor immunity. J. Immunol. (2007) 179: 775–780

[33] King EC, Blacker AJ, Bugg TDM. Enzymatic breakdown of poly-γ-D-glutamic acid in Bacillus li-cheniformis: identification of a polyglutamyl-γ-hydrolase enzyme. Biomacromolecules (2000) 1: 75-83

[34] Kubota H, Nambu Y, Endo, T. Convenient and quantitative esterification of poly-γ-glutamic acid pro-duced by microorganism. J. Polym. Sci. Part A: Polym. Chem. (1933) 31: 2877-2878.

[35] Kubota H, Matsunobu T, Uotani K, Takebe H, Satoh A, Tanaka T, Tanguchi M. Production of poly-γ

-glutamic acid by Bacillus subtilis F-2-01. Biosci. Biotechnol. Biochem. (1993) 57: 1212-1213

[36] Kubota H, Nambu Y, Endo T. Alkaline hydrolysis of poly-γ-glutamic acid produced by microorganism.

J. Poly. Sci. Chem. (1996) 34: 1347-1351

[37] Matsumoto K, Oki A, Furuta R, Maeda H, Yasugi T, Takatsuka N, Hirai Y, Mitsuhashi A, Fujii T, Iwasaka T, Yaegashi N, Watanabe Y, Nagai Y, Kitagawa T, Yoshikawa H. Tobacco smoking and regression of low-grade cervical abnormalities. Cancer Sci. (2010) 101: 2065-2073

[38] Monteiro DL, Trajano AJ, Russomano FB, Silva KS. Prognosis of intraepithelial cervical lesion during adolescence in up to two years of follow-up. J Pediatr. Adolesc. Gynecol. (2010) 23:230-236

[39] Moscicki AB, Shiboski S, Hills NK, Powell KJ, Jay N, Hanson EN, Miller S, Canjura-Clayton KL, Farhat S, Broering JM, Darragh TM. Regression of low-grade squamous intra-epithelial lesions in young women.

Lancet (2004) 364: 1678-1683

[40] Sung MH, Park C, Kim CJ, Poo H, Soda K, Ashiuchi M. Natural and Edible Biopolymer Poly-γ-glutamic acid: Synthesis, Production, and Applications. Chem Rec. (2005) 5: 352-66.

[41] Tanaka T, Yaguchi T, Hiruta O, Futamura T, Uotani K, Satoh A, Taniguchi, M, Oi S. Screening for microorganism having poly-γ-glutamic acid endohydrolase activity and the enzyme production by Myrothecium sp. TM-4222. Biosci. Biotechnol. Biochem. (1993) 57: 1809-1810

[42] Tanaka T, Hiruta O, Futamura T, Uotani K, Satoh A, Taniguchi M, Oi S. Purification and characte-rization of poly-γ-glutamic acid hydrolase from a filamentous fungus, Myrothecium sp.

TM-4222. Biosci. Biotechnol. Biochem. (1993) 57: 2148-2153

[43] Thorne CB, Gomez CG, Blind GR, Housewright, RD. Synthesis of glutamic acid and glutamyl po-lypeptide by Bacillus anthracis. III. Factors affecting peptide production in synthetic liquid media. J.

Bacteriol. (1953) 65: 472-478

[44] Thorne CB, Gomez CG, Noyes HE, Housewright RD. Production of glutamyl polypeptide by Bacillus subtilis. J. Bacteriol. (1954) 68: 307-315

[45] Troy FA. Chemistry and biosynthesis of the poly(γ-D-glutamyl) capsule in Bacillus licheniformis. 1. Properties of the membrane-mediated biosynthetic reaction. J. Biol. Chem. (1973) 248: 305-316

[46] Torii, M. Studies on the chemical structure of bacterial glutamyl polypeptides by hydrazinolysis. J. Biochem. (1959) 46: 189-200

[47] Zanuy, D, Aleman, C, Muñoz-Guerra S. On the helical conformation of un-ionized poly (γ-D-glutamic acid). Int. J. Biol. Macromol. (1998) 23: 175-184

## [23] Attachment List

Attachment 1. Sub-Investigators

Attachment 2. Study Pharmacist

Attachment 3. Monitor List

Attachment 4. Clinical Trial Patient Compensation Rule
