# Supplementary material for: Short-term clinical and immunologic effects of poly-gamma-glutamic acid (γ-PGA) in women with cervical intraepithelial neoplasia 1 (CIN 1): A multicenter, randomized, double blind, phase II trial
Source: PLoS One. 2019 Jun 20;14(6):e0217745. doi: 10.1371/journal.pone.0217745 (PMC6586279; doi:10.1371/journal.pone.0217745)
Supplement: S2 Text — (PDF) [file pone.0217745.s002.pdf]

임상시험 계획서

Protocol No. UMT2012-BL-PGA-01

가임기 여성의 자궁경부상피이형증(CIN Ⅰ)을 대상으로 한 폴리감마글루탐산(γ-PGA)의 효과와 안전성 평가를 위한 다기관, 무작위배정, 이중맹검, 위약대조, 평행설계, 2b 임상시험
A multi-center, randomized, double blind, placebo control, parallel design, phase 2b trial to evaluate the efficacy and safety of PGA (Poly-gamma-glutamic Acid) for the fertile women with Cervical Intraepithelial Neoplasia(CIN I)

책임연구자 : 가톨릭대학교 서울성모병원 교수 박종섭
고려대학교구로병원 교수 이재관
제일병원 교수 김태진
계명대학교 동산의료원 교수 조치흠
강남차병원 교수 성석주
강서 미즈메디병원 과장 박용수

작성일자 : 2015년 08월 05일
(UMT2012-BL-PGA-01, Version. 4.8)

- 기밀유지 -

본 임상시험계획서에 포함된 모든 정보는 임상시험책임 및 임상시험 담당자, 임상시험심사위원회, 보건당국을 위해 제공된 것으로서, 임상시험에 사용되는 의약품을 투여 받는 사람에게 시험 참가에 대한 서면 동의를 받기 위한 경우를 제외하고는 (주)바이오리더스의 사전 서면 동의 없이 제 3자에게 공개될 수 없습니다.

약어의 정의

CD: Cluster differentiation
CD8: Killer T cell
CD56: NK cell
MHC: class II Major Histo-compatibility Complex class II
INF-γ: Interferon-γ
NK cell: Natural killer cell
TNF-α: Tumor necrosis factor-α

ECG: Electrocardiogram

AE: Adverse event
ADR: Adverse Drug Reaction
SAE: Serious adverse event

ITT: Intent-to-treat
PP: Per-Protocol

ASCUS: Atypical squamous cells of undetermined significance

LGSIL: Low grade squamous intraepitherial lesion

HGSIL: High grade squamous intraepitherial lesion

CIN: Cervical intraepitherial neoplasia

HPV: Human papillomavirus

ASCCP Consensus Guideline: American Society for Colposcopy and Cervical Pathology

임상시험계획서 요약

제목	가임기 여성의 자궁경부상피이형증(CIN Ⅰ)을 대상으로 한 폴리감마글루탐산(γ-PGA)의 효과와 안전성 평가를 위한 다기관, 무작위배정, 이중맹검, 위약대조, 평행설계, 2b 임상시험<br>A multi-center, randomized, double blind, placebo control, parallel design, phase 2b trial to evaluate the efficacy and safety of PGA (Poly-gamma-glutamic Acid) for the fertile women with Cervical Intraepithelial Neoplasia(CIN I)
단계 및 디자인	-2b<br>-다기관<br>-무작위배정, 이중맹검, 위약대조, 평행설계
실시기관 및 연구자	가톨릭대학교 서울성모병원 교수 박종섭<br>고려대학교구로병원 교수 이재관<br>제일병원 교수 김태진<br>계명대학교 동산의료원 교수 조치흠<br>강남차병원 교수 성석주<br>강서 미즈메디병원 과장 박용수
의뢰자	(주)바이오리더스
목적	자궁경부상피이형증(CIN Ⅰ)의 가임여성을 대상으로 폴리감마글루탐산(γ-PGA)을 경구투여 시, 자궁경부상피이형증의 자연치유율 대비 치유율의 증가(면역치료 효과)와 안전성을 확인하며, 면역학적 지표 변화 평가를 통한 면역증강 효과를 확인하기 위함이다.
임상시험용 의약품 및 투여방법	-시험약: 폴리감마글루탐산 1,500mg<br>-대조약: 위약<br>-투여방법: 1일 1회, 취침 전, 경구복용
시험대상	가임여성으로 자궁경부상피이형증 진단을 받은 자로<br>병리소견 상 CIN I 으로 HPV 양성인 자
선정기준	1. 만 20세 이상 만 49세 이하의 가임 여성<br>2. 자궁경부상피이형증 CIN I 으로 진단을 받은 자<br>3. HPV 양성인 자<br>4. 스크리닝 검사 결과가 WBC: 4 $10^3/\mu L$ 이상, Hemoglobin: 9.0g/dL 이상, Platelet 150 $10^3/\mu L$이상, ANC(Absolute Neutrophil Count): 1,500 10*6/L 이상일 경우<br>5. AST/ALT: 정상범위 상한치의 2.5배 이내, BUN / Creatinine: 정상범위 상한치의 1.5배 이내인 경우

<table>
<tr><td></td><td>6. 심전도 검사에서 심장질환을 나타내는 비정상적 소견이나 흉부X-ray 검사에서 활동성 질환이 없는 자
7. 임상시험의 내용을 이해하고 참여에 동의하여 동의서에 자필로 서명한 자</td></tr>
<tr><td>제외기준</td><td>1. 자궁경부의 이형증 이외의 다른 장기의 악성 종양이 있는 자
2. 활동성 간질환, 면역관련질환, 또는 중증의 신부전이 있는 자
3. 백혈병, 교원질증, 다발성경화증, 자가면역질환 및 임상적으로 의미 있는 알레르기성 질환(투약을 필요로 하지 않는 경미한 알러지성 질환 제외)
4. 당뇨병으로 진단 받은 자
5. 7일 이내에 면역반응에 영향을 줄 수 있는 약물을 복용한 자(Glucocorticoid 제제, 비타민 제제, 건강식품, 한약 등)
6. 임신이 확인 되었거나 또는 수유 중인 자
7. 타 임상연구에 등록되어 있는 자
8. 기타 시험자가 부적합 하다고 판단한 자</td></tr>
<tr><td>목표
임상시험대상자 수</td><td>총 200명, 각 군당 100명
기관배정: 34명 (경쟁적 등록)
산정근거: CIN의 3개월간 자연치유율 31.0% 대비 20% 증가율을 고려하여 군당 80 명
<table>
<tr><th>군 구성</th><th>용량</th><th>임상시험 대상자 수</th><th>예상 치유율</th><th>치유예상 임상시험 대상자 수</th><th>투약기간/관찰기간</th></tr>
<tr><td>위약군</td><td>-</td><td>80명</td><td>31%</td><td>24.8명</td><td rowspan="2">4주간 1일 1회 투약 및 8주간 추가 관찰</td></tr>
<tr><td>시험군</td><td>1,500mg /day</td><td>80명</td><td>51%</td><td>40.8명</td></tr>
<tr><td>총</td><td></td><td>160명</td><td></td><td></td><td></td></tr>
</table>
자궁경부상피이형증(CIN)의 가임여성을 대상으로 폴리감마글루탐산(γ-PGA)을 경구투여 시, 자궁경부상피이형증 자연치유율 대비 치유율의 증가(면역치료 효과)를 확인하고, 안전성을 확인하며, 면역학적 지표 변화 평가를 통한 면역증강 효과를 확인하기 위한 2상 연구로 본 연구에서는 폴리감마글루탐산군의 치유율이 위약군에 비하여 20%이상 증가할 경우 대단위 3상 임상연구의 수행을 결정하는 연구로 위약군 대비 치유율의 20%를 고려할 때 통계적 가설은 다음과 같다.

$H_0 : p_1 \leqq p_2$ vs. $H_1 : p_1 > p_2$
p1: Treatment의 치유율, p2: Control의 치유율
기대하는 치유율: 시험군: 81%, 대조군: 51%
유의수준: 0.05, 검정력: 80%, 단측검정</td></tr>
</table>

<table>
<tr><td></td><td>
위 임상시험대상자수 산출 근거에 따라 위의 가설 미 예상 치율을 고려할 때 연구대상자 수는 Single stage 방법을 이용할 경우 군당 80명 총 160명 요구되며, 20% 중도탈락을 고려할 경우 군당 100명, 총 200명의 임상시험대상자수가 필요하다.
이외에 Jung(2008)·에 근거한 Two stage optimal design방법을 고려하여 임상시험대상자수를 산출하였는데, Jung(2008)의 방법은 Simon의 단일군 two stage optimal design을 비교군이 포함된 경우로 확장한 방법으로 이에 근거한 대상자 수는 다음의 표와 같다.

[Jung(2008)의 Two-stage 방법을 적용할 경우 다음과 같은 단계를 수행]
<table>
<tr><th>단계</th><th>총대상자수</th><th>종료 기준: 두 군의 차이 (Trt 치유 환자수-Con 치유 환자수)</th><th>실제 유의수준, 검정력</th></tr>
<tr><td>1</td><td>43</td><td>+2 명 미만</td><td rowspan="2">0.461, 0.800</td></tr>
<tr><td>2</td><td>80(43+37명)</td><td>+11 명 미만</td></tr>
</table>

즉 1단계에서 각 군에 43명을 배정하고, 1단계 종료 시 치유 환자수의 차이가 +2명 미만이면, 효과 없음으로 판정하여 연구종료하고, 그렇지 않을 경우 추가로 각 군당 37명을 모집하여 진행한다.
최종적으로 각 군당 80명의 대상자가 모집된 경우 두 군에서 치유된 환자 수의 차이가 11명 미만일 경우 효과 없음으로 판단하고, 11명 이상일 경우 효과 있음으로 판정한다.
위 Two stage optimal design방법에 따라 20%의 중도탈락을 고려할 경우 임상시험대상자 수는 1단계에서는 군당 54명씩 총 108명의 임상시험대상자가 필요하며, 2단계까지 추가모집을 진행할 경우 군당 100명씩 총 200명의 임상시험대상자수가 필요하다. 본 연구에서는 Jung(2008)에 기초하여 연구대상자 수를 설정하고, 2단계 디자인을 고려하기로 한다.
</td></tr>
<tr><td>시험기간</td><td>IRB 승인일로부터 48개월</td></tr>
<tr><td>시험방법</td><td>
임상시험대상자가 자발적으로 임상시험에 참여할 것을 동의하면 선정기준 및 제외기준을 확인하는 적합성 평가를 거쳐 폴리감마글루탐산 시험군 또는 위약대조군에 1:1의 비율로 무작위배정 되며, 이중맹검으로 4주간 임상시험용의약품의 투여와 8주간의 관찰을 포함하여 총 12주간 시험에 참여한다.

임상시험대상자의 방문은 총 5회로 -4주 이내(스크리닝, 방문1)과 0주(Baseline, 방문2), 4주(Observation, 방문3), 8주(Observation, 방문4), 12주(Closing, 방문5)이며 일정표에 따라 스크리닝을 위한 검사, 유효성과 관련된 자궁경부상피이형증의 진단 및 면역 기능 평가, 안전성과 관련된 실험실적 검사를 실시한다.
</td></tr>
</table>

병용금지 약물	1. 스테로이드계 약물: Betametasone, Dexametasone, Prednisolone, Hydrocortisone 등이며 국소적으로 피부나 눈 등에 적용하는 경우는 예외.<br>2. 면역조절물질: 성장호르몬, EPO(Erythropoietine), 알부민 제제, 전혈 및 백혈구 성분수혈 등<br>3. 기타: 임상시험용의약품 이외의 건강기능 보조식품 및 비타민 제제. 한약
시험중지 및 탈락기준	1. 임상시험용 의약품에 대하여 급성반응(알러지, 과민반응 등)을 보이는 경우<br>2. "중대한 이상반응"의 발생으로 지속적인 참여가 적절하지 않다고 시험자가 판단하는 경우<br>3. 선정/제외기준의 위반 또는 중대한 임상시험 계획서의 위반이 발생된 경우<br>4. 임상시험대상자가 임상시험용 의약품의 투여중단을 요구하거나, 임상시험 참여 동의를 철회하는 경우<br>5. 임상시험대상자의 불참으로 지속적인 관찰이 불가한 경우<br>6. 기타 시험자가 시험을 중지하여야 한다고 판단한 경우
평가방법	1. 유효성 평가 변수<br>1차 평가: 약물 투여 전 Baseline(-4주 이내) 대비 약물 투여 후 12주째 치유율의 시험군과 대조군의 비교<br>치유의 정의는 CIN I 의 단계에서 Normal로 된 경우를 의미 한다.<br><br>본 연구의 자궁경부상피이형증에서 변화의 단계는 다음과 같이 정의 한다.<br>-Regression: CIN I 단계에서 정상으로 치유된 경우<br>→CIN I 에서 Normal<br>-Persistence: CIN I 단계의 변화가 없이 동일한 경우<br>→CIN I 에서 CIN I<br>-Progression: CIN I에서 1단계 이상 증가하여 진행된 경우<br>→CIN I 에서 CIN II, CIN III, SCC(Squamous cell carcinoma)<br><br>약물 투여 전 Baseline 대비 약물 투여 후 Colposcopic biopsy 결과인 자궁경부상피이형증의 단계로 방문1, 방문5에서 Colposcopic biopsy를 실시한다.<br><br>2차 평가: 약물 투여 전 Baseline(-4주 이내) 대비, 약물 투여 후 12주째<br>-자궁경부상피이형증의 평가

	1) Reid Colposcopic Index 비교<br>2) Pap Smear Test 결과 비교<br>3) HPV DNA Chip Test 결과 비교<br>4) HPV HC Ⅱ assay 결과 비교<br><br>-면역기능 평가<br>:약물 투여 전 Baseline(0주) 대비, 약물 투여 후 4주와 8주, 12주째<br>1) 자연살해세포(NK cell) activity의 결과 비교<br>2) 말초혈액 단핵세포의 표현형 변화(MHC class II) CD8, CD56 population의 결과 비교<br>2. 안전성 평가변수<br>:약물 투여 전 Baseline(0주) 대비 약물 투여 후 4주와 8주, 12주째<br>이상반응, 활력징후, 실험실적 검사결과 비교
통계분석 방법	1. 분석의 일반적인 원칙<br>본 연구는 자궁경부상피이형증(CIN)의 가임여성을 대상으로 폴리감마글루탐산(γ-PGA)을 경구투여 시, 자궁경부상피이형증 자연치유율 대비 치유율의 증가(면역치료 효과)를 확인하고, 안전성을 확인하는 2b상 임상연구로 계획되었다. 본 연구에서 얻어지는 모든 자료는 연속자료의 경우 평균±표준편차, 범주형 자료는 대상자수(분율)로 정리요약하기로 하고, 필요시에는 다양한 요약통계량을 제시하기로 한다. 만약 구간추정치를 제시할 필요가 있을 경우 95% 신뢰구간을 제시하기로 한다.<br>본 자료의 통계적 분석에 있어 제 1 차 유효성 평가 기준인 치유율은 15.2에 정의한 적절한 유효성 평가 분석군에서 Jung (2008)의 방법에 기초하여 유의수준 5%에서 단측검정으로 실시하기로 한다. 1차 유효성 평가항목 이외의 분석에서는 유의수준 5%에서 양측검정을 실시하기로 한다. 15.3.4 와 15.3.6에서 제시된 유효성 평가 방법 및 안전성 평가 방법 외에 임상시험대상자의 연령, 위증도, 기저 실험실 수치 등 결과변수에 기여하는 예후 인자에 대해 군간 차이가 확인될 경우에는 예후인자를 보정하는 적절한 통계적 방법(예: logistic 모형, ANOCOVA, 일반화 선형 모형 등)을 추가적으로 적용하기로 한다.<br><br>2. 분석군의 정의<br>본 임상시험에서 유효성과 안전성을 평가하는데 필요한 분석집단은 국내 및 국제 기준에 따라 유효성 평가에 있어서는 아래에 정의하는 바와 같이 ITT 및 PP 분석군에서 모두 실시하고, 안전성 평가에 있어 아래의 정의에 따라 분석을 정의하고 이를 기준으로 평가한다.<br>유효성 평가 분석군:<br>- ITT(Intent-to-Treatment)군: 연구참여 동의 후 확률화 배정된 모든 대상자로 정의한다. 다만 ITT 군에서 연구약물을 최소 1번이라도 복용하지 않은 경우는 분석군에서 제외하기로 한다.

	- PP(Per Protocol)군: 연구참여 동의 후 연구의 종료 시까지 중대한 연구계획서 위반 사항이 없이 연구계획에 따라 임상연구를 완료한 대상자 전체로 정의한다.<br>- 유효성 평가에 ITT군과 PP군에서 모든 분석을 실시 및 결과보고에 제시하기로 하고, 최종 평가는 ITT군의 분석결과에 따라 결정하기로 한다. 만약 ITT군과 PP군의 결과가 상이할 경우, 두 분석결과가 상이한 이유를 탐색하는 추가적인 분석을 실시하기로 한다.<br>안전성 평가 분석군:<br>연구참여에 동의한 후 확률화 배정되어 최소 1회 이상 연구약물을 복용한 대상자 전체로 정의하기로 한다.<br><br>3. 일차 유효성 평가변수에 관한 분석<br>기저에서 CIN I의 약물 투여 3개월째에 정상으로 치유된 경우로 판정하고 시험군과 대조군 간의 치유율을 비교하는 것이다.<br>각 군간 치유율의 평가는 Jung (2008)이 제시한 방법에 기초하는데 앞에서 기술한 것과 같이 1단계 각 군에서 43명을 선정하여 결과를 확인하였을 때, 치유에 해당하는 임상시험대상자의 수의 차이가 +2명 미만(폴리감마글루탐산군-대조군)인 경우 폴리감마글루탐산의 효과가 없음으로 판정하고, 본 연구는 종료된다. 만약 1단계에서 +2명 이상 차이가 발생할 경우로 추가로 각 군당 37명을 모집하여 총 각 군당 80명을 대상으로 치유로 판정받은 임상시험대상자의 수가 +11명 이상이면 자궁경부상피이형증의 치유에 있어 폴리감마글루탐산의 효과가 있을 것으로 판단하게 된다. 만약 +11명 미만인 경우 폴리감마글루탐산의 효과는 최종적으로 없는 것으로 결론 짓게 된다.<br><br>4. 이차 유효성 평가변수에 관한 분석<br>2차 유효성 평가변수 1: Reid Colposcopic Index 비교<br>Reid Colposcopic Index는 CIN 병변에 대한 여러 소견들을 특징에 따라 분류하고 점수화한 Grading System로 0에서 8점까지 채점되며 점수에 따른 Grading은 아래와 같이 판정된다.<br>0-2점: Likely to be CIN I<br>3-4점: Overlapping lesion - likely to be CIN I or CIN II<br>5-8점: Likely to be CIN II<br>Reid Colposcopic Index의 분석에 있어 기저의 점수 대비 12주 후의 점수의 차이에 대한 군간 비교를 이표본 t-검정 또는 윌콕슨 순위합 검정을 통하여 확인하기로 한다. 또한 Grading체계(0-2점, 3-4점, 5-8점)를 multinomial 분포로 고려하여 기저 대비 12주 후의 Grading체계 변화의 군간 차이를 누적 또는 순서 로지스틱 모형(cumulative or ordinal logistic model)을 통해 분석하기로 한다.<br><br>2차 유효성 평가변수 2: Pap Smear Test 결과 비교

	자궁경부 세포검사는 자궁내경관에 brush(broom)의 긴 중앙부분을 넣어 동일한 방향으로 2~3회 회전 후 보존액이 담겨진 용기 안에 brush를 분리시켜 넣고 뚜껑을 닫는다. 판독기준은 **The 2001 Bethesda System for Reporting Cervical Cytologic Diagnoses** 에 의한다. 판독결과는 Negative, ASC-US, ASC-H, LSIL, HSIL, Invasive cancer(Cervical carcinoma)의 6단계로 나뉘는데, multinomial 분포로 고려하여 기저 및 연구종료 시에서의 군간 비교를 카이제곱 또는 정확검정을 통해 수행하고, 기저대비 3개월 후의 분류단계의 변화를 확인하기 위해 누적 또는 순서 로지스틱 모형(cumulative or ordinal logistic model)을 통해 분석하기로 한다.<br><br>2차 유효성 평가변수 3: HPV DNA Chip Test 결과 비교<br>HPV DNA Chip Test는 바이러스의 감염타입을 확인하기 위해 실시하는 시험이다. 시험결과에 의해 Positive 또는 Negative로 분류하게 되는데, 기저 대비 측정된 치유 여부에 대해서 시간의 따른 변화와 군간 차이 그리고 시간 및 군간 상호작용을 확인하기 위하여 GEE(Generalized Estimating Equation) 또는 일반화 선형 혼합 모형(Generalized Linear Mixed Model)을 고려하기로 한다.<br><br>2차 유효성 평가변수 4: HPV HC Ⅱ assay 결과 비교<br>HPV HC(Hybrid Capture) Ⅱ assay 는 자궁경부 상피내 종양의 중증도를 예측할 수 있는 방법의 하나로서 시행되는 검사이다. 검사결과는 시료의 빛의 단위를 기준으로 Positive 또는 Negative로 분류되는데, 이항 분포로 고려하여 기저 및 연구종료 시에서의 군간 비교를 카이제곱 또는 정확검정을 통해 수행하고, 기저 대비 12주 후에 측정된 치유 여부에 대해서 시간의 따른 변화와 군간 차이 그리고 시간 및 군간 상호작용을 확인하기 위하여 GEE(Generalized Estimating Equation) 또는 일반화 선형 혼합 모형(Generalized Linear Mixed Model)을 고려하기로 한다.<br><br>2차 유효성 평가변수 5: 자연살해세포(NK cell) activity의 결과 비교<br>기저(0주) 대비 4주 및 8주, 12주 후에 측정된 자연살해세포(NK cell) activity의 결과 비교를 위해 기저 및 12주 후의 자연살해세포(NK cell) 수치 차이에 대한 평균 비교를 t-검정 또는 윌콕슨 순위합 검정을 통해 수행할 것이다.<br>2차 유효성 평가변수 6: 말초혈액 단핵세포의 표현형 변화 CD8, CD56 population의 결과 비교<br>기저(0주) 대비 12주 후에 측정된 말초혈액 단핵세포의 표현형 변화(MHC class II) CD8, CD56 population의 결과 비교에 있어 표현형의 변화에 대해 기저 및 12주 후의 변화 여부에 대해 군간 비교를 위해 카이제곱 검정 또는 정확 검정법을 사용하고, 기저 대비 12주 후에 측정된 치유 여부에 대해서 시간의 따른 변화와 군간 차이 그리고 시간 및 군간 상호작용을 확인하기 위하여 GEE(Generalized Estimating Equation) 또는 일반화 선형 혼합 모형(Generalized Linear Mixed Model)을 고려하기로 한다.

	5. 안전성 평가변수에 관한 분석<br>대상자로부터 수집된 모든 이상반응, 임상 실험실적 검사치, 12-lead ECG, 및 활력 징후(혈압 및 맥박수) 등 모든 자료에 대해 안전성 평가를 수행한다.<br>1) 이상반응<br>시험약 투여 후 관찰된 이상반응을 요약한다. 이상반응, 이상약물반응, SAE, 사망, 임상시험 중단을 초래한 이상반응, 및/또는 “기타 유의한 이상반응(OAE)”을 경험한 임상시험대상자의 수, 발생 건수를 각 치료군 별로 요약한다. 각 이상반응을 나타낸 임상시험대상자 수 또한 각 치료군 별로 신체기관계(SOC), 권장용어 및 최대 중증도 별로 요약한다. 요약 통계량 이외에 군간 이상반응 발생비율 또는 발생건수는 Chi-squared Test, Fisher’s Exact Test 또는 Poisson test를 이용하여 분석한다.<br>2) 활력징후 및 실험실 검사<br>실험실적 검사치, 12-lead ECG의 이상치 발현 정도의 각 시점별로 군간 비교를 위해 Chi-squared Test, Fisher’s Exact Test 또는 Poisson test를 이용하여 분석하고, 활력징후에 대해서도 매 시점별로 연속형 자료의 요약통계량을 제시하고, 필요시 GLM 또는 GLMM 방법을 이용하여 군간 비교를 실시하기로 한다.

[임상시험 흐름도]

관찰 및 검사 항목 및 방법

Visit Type	Screening	Baseline & Treatment	Observation	Observation	Closing
Visit No.	1	2	3	4	5
Visit Week	-4주 이내	0주	4주±5일	8주±5일	12주±5일
서면 동의	V				
기초정보 조사	V				
선정/제외기준 검토	V				
과거력[1]	V				
신체검사/흉부X선 검사	V				
활력징후	V	V	V	V	V
실험실 검사[2]	V		V	V	V
임신반응 검사[3], HbA1c[4]	V				
ECG	V				V
Reid Colposcopic Index[6]	V				V
Colposcopy with Biopsy[5]	V				V
Pap Smear Test[6]	V				V
HPV DNA Chip Test[6]	V				V
HPV HC Ⅱ[6]	V				V
NK Cell Activity[6]		V	V	V	V
PBMCs 면역표현형 검사[6]		V	V	V	V
배정번호 부여		V			
시험약품교부		V			
복용 순응도			V		
이상반응			V	V	V
병용약물			V	V	V

1. 과거력
   1) 병력 및 수술력: 스크리닝 방문 기준으로 관련 질환은 2년 이내, 기타 질환은 6개월 이내
   2) 복용약물: 스크리닝 방문 기준으로 4주 이내에 투여하였거나 현재 투여 중인 약제
2. 실험실적 검사 항목
   1) 혈액학적 검사: Hct, Hb, WBC(diff. count포함), RBC, Platelet, ANC
   2) 혈액응고 검사: aPTT, PT

3) 일반화학 검사: Glucose, AST, ALT, Alkaline phosphatase, Total Protein, Albumin Total Bilirubin, γ-GTP, Cholesterol (Total, LDL, HDL), Triglyceride, BUN, Creatinine, Uric acid, Sodium, Calcium, Potassium

4) 뇨검사: PH, Nitrite, Ketone, Specific gravity, Glucose, Protein, Urobilinogen, RBC

3. 임신반응 검사: Urine hCG
4. 당뇨병 검사: 당화혈색소 HbA1c
5. 1차 유효평가변수: Colposcopic Biopsy (생검)

동의취득 전 4주 이내의 검사결과는 스크리닝 검사로 인정.

(단, 타 기관의 검사결과를 사용 시 최소한 결과를 확인할 수 있는 조직검사 결과지가 확보되어야 함.)

6. 2차 유효평가 변수:

<u>자궁경부 상피이형증 평가</u>

1) Reid Colposcopic Index
2) Pap smear test (세포진 검사)
3) HPV DNA Chip test(감염 Type 및 소실 검사)
4) HPV Hybrid Capture Ⅱ assay(viral load 정량검사)

<u>면역기능 평가</u>

1) 자연살해세포(NK cell) activity의 측정
2) 말초혈액 단핵세포의 표현형 변화(MHC class II) CD8, CD56 population의 측정

목 차

약어의 정의 2
임상시험계획서 요약 3
[1] 임상시험의 명칭 및 단계 14
[2] 임상시험의 실시기관명 및 주소 15
[3] 임상시험의 책임자, 담당자 및 공동연구자의 성명 및 직명 16
[4] 임상시험용 의약품등을 관리하는 약사의 성명 및 직명 17
[5] 임상시험의 의뢰자명 및 주소 18
[6] 임상시험의 목적 및 배경 19
[7] 임상시험용 의약품등의 코드명이나 주성분의 일반명, 원료약품 및 그 분량, 제형 등 23
[8] 대상질환 25
[9] 임상시험대상자의 선정기준, 제외기준, 목표한 임상시험대상자의 수 및 그 근거 26
[10] 임상시험의 기간 29
[11] 임상시험의 방법 (투여-사용량, 투여-사용방법, 투여-사용기간, 병용요법등) 30
[12] 관찰항목·임상검사항목 및 관찰검사방법 32
[13] 예측 부작용 및 사용상의 주의사항 45
[14] 시험중지·탈락기준 및 분석제외 기준 46
[15] 효과 평가기준, 평가방법 및 해석방법(통계분석방법) 47
[16] 부작용을 포함한 안전성의 평가기준, 평가방법 및 보고방법 53
[17] 임상시험대상자 동의서 양식 57
[18] 임상시험대상자 보상 규약 58
[19] 임상시험 후 임상시험대상자의 진료 및 치료기준 59
[20] 임상시험대상자의 안전보호에 관한 대책 60
[21] 그 밖에 임상시험을 안전하게 과학적으로 실시하기 위하여 필요한 61
[22] 참고문헌 63
[23] 별첨 목록 66

[1] 임상시험의 명칭 및 단계

가임기 여성의 자궁경부상피이형증(CIN Ⅰ)을 대상으로 한 폴리감마글루탐산(γ-PGA)의 효과와 안전성 평가를 위한 다기관, 무작위배정, 이중맹검, 위약대조, 평행설계, 2b 임상시험

A multi-center, randomized, double blind, placebo control, parallel design, phase 2b trial to evaluate the efficacy and safety of PGA (Poly-gamma-glutamic Acid) for the fertile women with Cervical Intraepithelial Neoplasia(CIN I)

[2] 임상시험의 실시기관명 및 주소

2.1 임상시험 실시기관

실시 기관	주소	전화
가톨릭대학교 서울성모병원	서울시 서초구 반포동 505	1588-1511
제일병원	서울시 중구 묵정동 1-19	02-2000-7070
고려대학교 구로병원	서울시 구로구 구로동로 148	02-2626-0114
계명대학교 동산의료원	대구시 중구 달성로 56	053-250-7114
강남차병원	서울시 강남구 논현로 566	02-3468-3000
강서 미즈메디병원	서울시 강서구 내발산동 701-4	1588-2701

2.2 참여기관

참여 기관	주소	전화
한국생명공학연구원	대전광역시 유성구 과학로 111	042-860-4157

[3] 임상시험의 책임자, 담당자 및 공동연구자의 성명 및 직명

3.1 시험조정자

성명	소속 기관명	직명	전화
이재관	고려대학교 구로병원	교수	02-2626-3142

3.2 시험책임자

성명	소속 기관명	직명	전화
박종섭	가톨릭대학교 서울성모병원	교수	02-2258-2722
이재관	고려대학교 구로병원	교수	02-2626-3142
김태진	제일병원	교수	02-2000-4713
조치흠	계명대학교 동산의료원	교수	053-250-7518
성석주	강남차병원	교수	02-3468-3023
박용수	강서 미즈메디병원	과장	02-2007-1316

3.3 공동연구자

별첨1. 공동연구자 참조

[4] 임상시험용 의약품등을 관리하는 약사의 성명 및 직명

별첨2. 임상시험용 의약품 관리자 참조

[5] 임상시험의 의뢰자명 및 주소

5.1 의뢰자

회사명	대표이사	소재지	전화
㈜바이오리더스	성문희	대전광역시 유성구 용산동 559번지	042-934-7671

5.2 모니터요원

별첨 3. 모니터 요원 참조

[6] 임상시험의 목적 및 배경

6.1. 목적

자궁경부상피이형증(CIN)의 가임여성을 대상으로 폴리감마글루탐산(γ-PGA)을 경구투여 시, 자궁경부상피이형증 자연치유율 대비 치유율의 증가(면역치료 효과)를 확인하고, 안전성을 확인하며, 면역학적 지표 변화 평가를 통한 면역증강 효과를 확인하기 위함이다.

6.1.1 자궁경부상피이형증

자궁경부상피이형증(Cervical Intraepitherial Neoplasia)은 자궁경부암의 전암 단계 질환으로 발병원인 바이러스인 인유두종바이러스(HPV, human papillomavirus) 감염에 의해 발생되며, 조직학적 진단법에 따라 1, 2, 3기 (CIN I, II, III) 단계를 거쳐 자궁경부암으로 발전된다.[1][2]

자궁경부상피이형증 환자는 세계적으로 매년 3,000만명 수준이 발생하며, 국내에서도 매년 약 20만명의 환자가 발생하는 것으로 추산되고 있다. 국내의 경우 국가 암 예방 조기발견 정책으로 암 검진 사업이 이루어지면서 질환의 조기발견으로 인해 자궁경부암 환자 수는 감소하고 있으나, 전암단계인 자궁경부상피이형증 환자의 수는 급격히 증가하여 건강보험심사원의 발표에 의하면 2007년에서 2011년 사이에 34%의 증가율을 보였다.

자궁경부암 발생에 가장 중요한 인자인 HPV 감염 예방을 위한 고위험 HPV 양성 자궁경부암 예방백신이 출시되었으나, 대한산부인과학회의 조사에 의하면 2009년~2010년의 국내 백신 접종률은 3.3%로 나타났다. 높은 가격, 백신 접종으로 모든 type의 HPV감염을 예방할 수 없다는 점, 6개월에 걸쳐 3회의 내원 접종이 필요한 주사제라는 점 등으로 접종율은 크게 증가되지 못하고 있다.

또한 첫 성경험 연령이 가파른 속도로 내려오고 있다는 것도 문제로 지적 된다. 20~30대의 가임기가 되었을 때 자궁경부암 위험이 크게 증가할 수 있기 때문이다. 자궁경부상피이형증으로 자궁경부원추절제술을 시행한 과거력이 있는 여성은 그렇지 않은 여성과 비교했을 때 조기 진통, 조산, 조기양막 파수, 신생아 저체중 등의 합병증이 유의하게 높게 발생하는 것으로 알려져 있다.[3] 자궁경부이형증 초기 단계의 경우에도 길게는 3년까지 4~6개월 단위로 내원 검진을 통해 질환의 진행 여부를 확인해야 하기 때문에 장기간의 암으로의 진행 여부에 대한 정신적인 고통 및 의료비 부담이 필요한 상태이다.

따라서 자궁경부상피이형증 (CIN I)으로 진단된 젊은 가임기 여성의 경우 비수술적 방법이 우선적으로 고려되어야 하며, 질환 초기 단계 환자들 또한 비수술적 방법을 통한 빠른 치유를 필요로 하고 있다. 가임기 여성의 자궁경부암 진행을 예방하며 가임률을 높이고 안전한 출산을 돕는 효과적인 방법이자, 질환의 치유율을 높이고 치유기간을 앞당기는 측면에서 (주)바이오리더스의 폴리감마글루탐산 면역치료제가 기여할 수 있을 것으로 기대한다.

6.2. 배경

본 임상시험용의약품의 주성분인 폴리감마글루탐산(poly-gamma-glutamic acid; γ-PGA)은 천년 이상

먹어왔던 우리 전통 콩 발효식품인 청국장에 존재하는 끈적끈적한 점액성의 물질이다. 폴리감마글루탐산은 글루탐산(glutamic acid)이 감마 펩타이드 결합에 의해 연결된 음이온성 고분자 물질로 GRAS (generally recognized as safe) 미생물인 바실러스에 의해 생산되는 생물학적 안전성이 확보되어 있는 바이오 고분자 소재이다. 2010년 일본의 아지노모토社에서 미 FDA에 제시하여 salt substitute product의 debittering agent와 KCl을 포함하는 FOOD 항목이 GRAS로 지정되었다. 폴리감마글루탐산은 우리나라의 청국장과 유사한 일본의 콩 발효식품이 낫또에도 존재하며, 일본에서는 오래 전부터 다양한 연구가 진행되어 식품 첨가물과 특정 보건용 식품에 이미 사용되고 있으며, 천연 고분자 물질이 가지는 다양한 기능성에 대한 많은 연구가 이루어지고 있는 소재이다.

바이오리더스는 한국식품의약품안전처(MFDS)로부터 폴리감마글루탐산과 관련되어 2003년 식품원료(식규 65421-326), 2006년 천연 식품첨가물(제60호), 2009년 건강기능식품 개별인정형 기능성 원료로 국내 최초로 승인 받은 바 있다.

바이오리더스는 약 10년간의 연구를 통해 국내 순수 기술로 일본의 낫또 유래의 바실러스 균에 비해 물리·화학적, 생물학적 특성이 우수한 폴리감마글루탐산을 생산 정제하는 기술을 확보하였으며, 다양한 용도 개발을 수행하였고 이를 통해 기존에 알지 못했던 다양한 폴리감마글루탐산의 기능을 입증하였다. 새롭게 확인된 기능성 중 고분자량 폴리감마글루탐산이 가지는 면역증강 활성이 면역치료를 필요로 하는 질환에 대한 치료제로서의 개발 가능성을 동물 효력시험들을 통해 확인하였으며, 이를 바탕으로 면역치료제 개발을 진행하게 되었다.

청국장을 통해 암을 극복한 많은 보고들이 되어져 왔으며, 실제로 국내 및 일본의 많은 사람들이 청국장(일본 낫또)을 민간 면역요법의 일환으로 섭취하고 있다. 실제로 대장암, 신장암, 위암, 유방암 등 암환자들의 암 극복 성공사례들이 많이 보고되어 짐에 따라 청국장의 성분 중 어떠한 성분이 항암효과를 가지는 지에 대한 많은 연구들이 진행되어 왔다. 청국장으로 알려진 항암효과를 가진 성분 중 이소플라본 계열의 제니스틴이 알려져 있으나, 실제로 항암 효과를 나타낼 수 있을 만큼의 충분한 양으로 보기는 어려운 점이 있었고, 실제로 생청국장에서 추출한 폴리감마글루탐산(γ-PGA)이 면역활성 (대식세포 처리 시, 면역유도 관련 사이토카인인 TNF-α와 IFN-β의 분비를 유도하는 활성)을 가지는 것을 확인하였다.

(주)바이오리더스는 한국생명공학연구원과의 공동 연구를 통해 고분자량의 폴리감마글루탐산이 항암면역 증강 기능을 가지는 것을 세계 최초로 입증하였으며(*J. Immunol.* 2007, *Cancer Immunol. Immunother.* 2009)[4], 시중에 판매되고 있는 제품들을 분석 해 본 결과 50 g의 생청국장 1팩에 약 150 mg 정도의 폴리감마글루탐산이 함유되어 있음을 확인할 수 있었다. 폴리감마글루탐산은 삶아진 콩을 발효하는 *Bacillus subtilis* 균의 종류에 따라 다른 물리화학적, 생물학적 특성(평균분자량, 점도, 면역활성 등)이 다르기 때문에 동일한 효능을 기대할 수는 없을 것으로 판단하여, 의약품 grade의 폴리감마글루탐산을 주성분으로 하는 신규 의약품을 개발하고자 생산 균주, 발효 및 정제 공정 확립을 통해 고순도로 정제된 폴리감마글루탐산을 확보하였고, 이를 이용한 비임상 및 임상시험을 진행하여 새로운 의약품으로 개발하고자 연구를 진행하고 있다.

본 연구에서는 순수 정제된 폴리감마글루탐산을 자궁경부상피이형증 여성에게 경구 투여하여 면역치료 효과를 평가하기 위한 무작위배정, 이중맹검, 위약대조 임상시험을 수행하고자 한다.

6.3 생물학적 성질 중 면역증강 기능성 개요

대상 질환인 자궁경부상피이형증은 체내 면역 작용에 의해 발병자 중 일정 비율이 자연치유 되는 특성을 가지고 있다. 자연치유와 관련된 면역 기작이 명확하게 규명된 상태는 아니지만 다수의 연구결과를 종합해 볼 때, 바이러스 감염에 따른 선천 면역 작용 및 바이러스 항원 특이 세포성 면역 작용 등이 관여하는 것으로 알려져 있으며, 자연 치유가 이루어지지 않고 질환이 진행되는 것은 면역회피 기전에 의한 것으로 알려져 있다.

지금까지 규명된 개발 대상 의약품의 주성분인 폴리감마글루탐산의 면역치료 작용 기작은 크게 3가지로 1) 바이러스 초기 감염 예방 및 치료에 효과적인 innate immunity 활성화, 2) 바이러스 감염 또는 이형화된 세포 제거에 작용할 수 있는 자연살해세포(natural killer cell) 및 세포독성 T 세포(cytotoxic T cell) 등의 세포성 면역 활성화 유도, 3) 질환의 진행을 억제 또는 치유하는데 관련된 체내 면역작용을 회피하는 기전을 억제하는 기작 등으로 나누어 볼 수 있다.

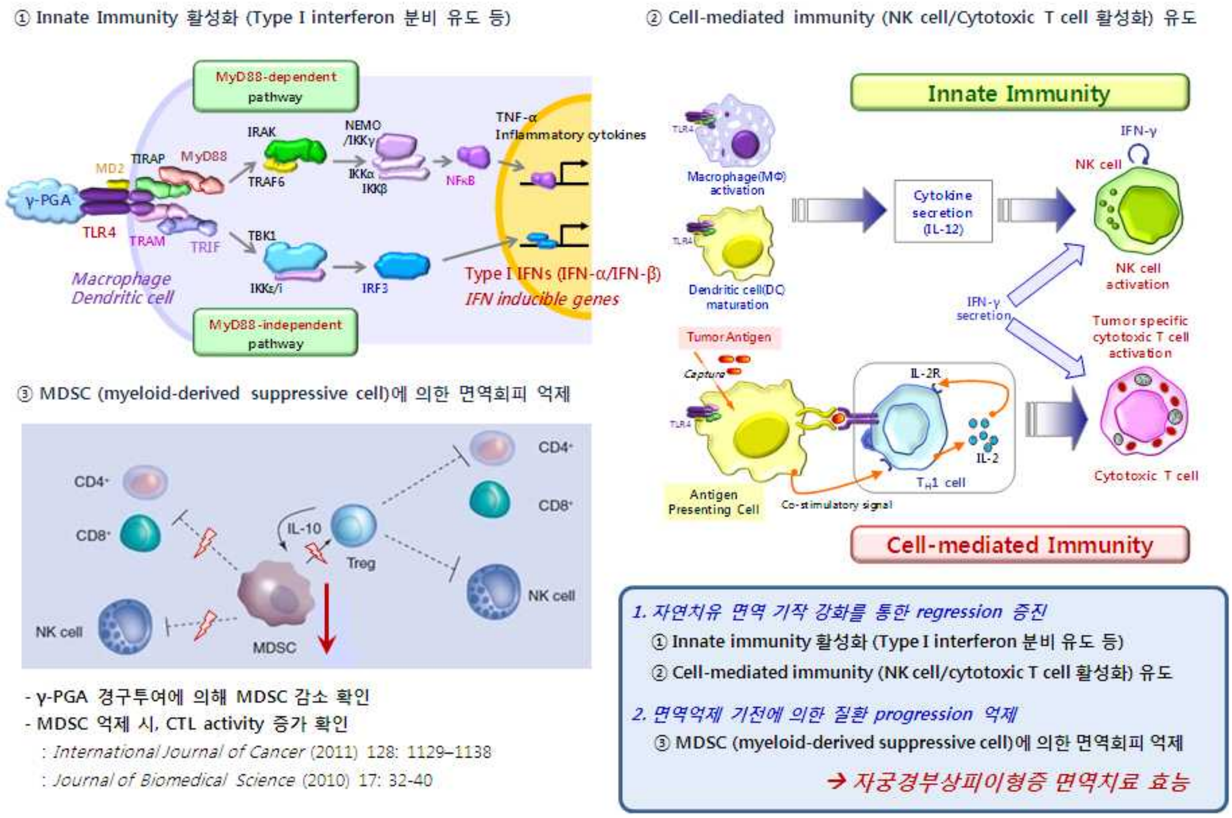

폴리감마글루탐산의 경구투여를 통한 면역증강 효력은 소장 점막에 존재하는 면역세포인 대식세포(macrophage cell)와 수지상세포(dendritic cell, DC)의 활성화를 통해 유도되며, 궁극적으로 혈액 중 자연살해세포(natural killer cell) 등의 세포성 면역을 유도하는 것으로 확인되었으며 (*J. Immunol.* 2007), 자연살해세포 및 세포독성 T 세포 등의 세포성 면역을 활성화시키는 항암면역의

기전은 대식세포(macrophage cell)와 수지상세포(dendritic cell, DC) 표면에 존재하는 receptor인 toll-like-receptor 4 (TLR4)를 매개로 하는 것이 확인되었다(Cancer Immunol. Immunother. 2009).

6.4 선행 임상 연구 결과

1) 가톨릭대학교 서울성모병원 김경수 교수 폴리감마글루탐산 8주간의 투여가 사람의 면역력 증강에 미치는 영향을 평가하기위한 단일기관 무작위배정, 이중맹검,무작위 배정, 이중맹검, 위약대조 임상시험
   - 감마글루탐산 저용량군(500mg) 33명, 감마글루탐산 고용량군(1000mg) 33명, 위약군 33명의 총 99명이 참가한 임상시험으로 일차 유효성 평가를 위해 기저 시점 대비 각 시점(4주, 8주)에서의 natural killer 세포의 cytotoxicity 평균 변화량의 차이를 세 군간 비교하기 위하여 ITT 임상시험대상자 총 99명에 대하여 각 군별로 기저 시점과 각 시점(4주, 8주)의 자연살해세포의 cytotoxicity 평균 변화량을 자료의 정규성 검토를 토대로 Kruscal-Wallis test를 사용하여 p-value<0.05를 만족하면서 통계적으로 유의한 차이가 있다고 판단하였다.
   - 기저 시점 대비 각 시점(4주, 8주)에서의 자연살해세포의 cytotoxicity 평균 변화량을 비교한 결과 기저 시점 대비 4주에서의 평균 변화량을 살펴보면, 저용량군은 0.5±3.5% 증가(증가율:4.5%), 고용량군은 4.3±7.5% 증가(증가율:39.1%), 위약군은 0.3±3.3%로 증가(증가율:2.7%)하였으며, 통계적으로 유의한 차이가 있음을 보였다(p-value=0.0174). 다중비교 결과 저용량군과 고용량군, 고용량군과 위약군에서 p-value=0.0013). 다중비교 결과 저용량군과 고용량군, 고용량군과 위약군에서 cytotoxicity 변화량에 통계적으로 유의한 차이를 보였으며, 고용량군에서의 cytotoxicity 증가율이 저용량군과 위약군에 비해 증가율이 높음을 알 수 있었다.
   - 이차유효성으로 말초혈액 단핵세포의 표현형 변화(MHC class Ⅱ) : CD3, CD4, CD8, CD14, CD56에 대한 세 군간 변화량을 비교한 결과, CD8 항목이 기저 시점 대비 4주 시점에서 p-value는 0.0248로 통계적으로 유의한 차이가 있음을 보였으며, CD56 항목이 기저시점 대비 4주와 8주 시점에서 p-value=0.0157, p-value=0.0002로 통계적으로 유의한 차이가 있음을 보였다. 다중비교 결과 두 항목(CD8, CD56) 모두 저용량군과 고용량군, 고용량군과 위약군에서 변화량에 차이가 있었다. 안전성 평가에서는 이상반응으로 인한 중도탈락 임상시험대상자는 없었으며, 중대한 이상반응은 나타나지 않았다. 혈압과 맥박 모두 세 군간 유의한 차이를 보이지 않았으며, 실험실 검사 상에서 이상소견이 발견되지 않았다.

2) 건강한 남성 임상시험대상자를 대상으로 폴리감마글루탐산의 경구 투여 후 안전성, 내약성 및 약동학적 특성을 평가하기 위한 무작위 배정, 위약대조, 단회/반복 투여, 단계적 증량 임상 1상 시험
   - 건강한 남성자원자에게 폴리감마글루탐산을 경구 투여하였을 때, 3.0gram/day 용량까지 안전성과 내약성을 보였다.

[7] 임상시험용 의약품등의 코드명이나 주성분의 일반명, 원료약품 및 그 분량, 제형 등

7.1 임상시험에 사용되는 임상시험용 의약품의 개요

7.1.1 시험약품

- 개 발 명 : BLS-PGA
- 성 상 및 제 형 : 투명한 점성이 있는 시럽이 들어 있는 갈색 유리병
- 저 장 방 법 : 냉장 보관(2℃~8℃), 기밀용기
- 재 검 사 일 자 : 제조일로부터 24개월까지 '의약품 등 안정성 시험기준(식약처고시)' 등에 따라 자체 재검사
- 원료약품 및 그 분량 : 1병(100mL) 중,

함량기준	배합목적	성분명	규격	분량	단위
100 mL 시럽제 중	주성분	폴리감마글루탐산용액<br>(폴리감마글루탐산으로1,500mg포함)	별규	93	g
	감미제	아스파탐	식첨	30	mg
	감미제	스테비오사이드	식첨	10	mg
	보존제	파라옥시벤조산메틸	KP	80	mg
	보존제	파라옥시벤조산프로필	KP	20	mg
	용제	에탄올	KPC	3	g
	용제	정제수	KP	3.86	g

7.1.2 대조약품: 위약품

- 성상 및 제형 : 투명한 점성이 있는 시럽이 들어 있는 갈색 유리병
- 저장 방법 : 냉장 보관(2℃~8℃), 기밀용기
- 재검사일자 : 제조일로부터 24개월까지 '의약품 등 안정성 시험기준(식약처고시)' 등에 따라 자체 재검사
- 원료약품 및 그 분량 : 시험약에서 주성분이 제외된 동일한 성분 및 분량

7.2 시험약품의 투여방법

1일 1회, 취침 전 1병씩 경구 복용

7.3 포장 및 라벨링

7.3.1 시험약품의 포장

액상제형이 들어있는 병으로 1회 복용 가능한 100mL 단위로 포장되어 있다.

시험약과 대조약이 구별되지 않도록 동일한 외형의 갈색 유리병에 동량으로 포장하여 이중맹검을 유지하도록 한다.

임상시험약품의 임상시험대상자 불출 시의 지급 포장은 4주 분에 추가 5일의 여유분을 포함한다.

7.3.2 임상시험용 의약품의 라벨링

임상시험에 사용되는 약품의 용기 및 포장 라벨은 약사법 시행규칙 제 75조 제6항에 준하여 다음의 내용을 표시하도록 한다.

1) "임상시험용"이라는 표시
2) 제품의 코드명 또는 주성분의 일반명
3) 제조번호 및 사용(유효)기한 또는 재검사일자
4) 저장방법
5) 임상시험계획 승인을 받은 자의 상호와 주소
6) "임상시험 외의 목적으로 사용할 수 없음"이라는 표시

7.4 임상시험용 의약품의 관리 및 보관 방법

7.4.1 약품의 보관/교부/관리

시험책임자는 의뢰자로부터 배송 된 임상시험용 의약품이 담당자에 의해 정확히 수령되고, 안전한 장소에 보관됨을 확인해야 하며 임상시험용 의약품이 교부 전에 손상되지 않도록 보존할 책임이 있다. 본 연구에서 사용되는 임상시험용 의약품을 임상시험 이외의 목적으로 사용되지 않도록 관리한다. 관리약사는 보관 시 눈금 온도계 또는 열전대를 사용하여 실제 온도를 기록하며, 최소한 근무일에는 측정한 온도를 일지에 기록해야 한다. 허용되는 범위를 벗어난 온도편차가 있을 경우는 담당 모니터에게 반드시 연락해야 한다. 온도일지는 임상시험 종결 후 시험기관 연구자 파일에 보관되어야 한다.

의뢰자는 임상시험 실시 중 임상시험용 의약품의 수량 및 보관상태 등을 확인하고 임상시험이 적절히 진행될 수 있도록 조치하여야 한다.

각 개별 임상시험대상자 포장은 시험기관에서 추가 재포장 또는 라벨 부착 없이 의뢰사에서 제공한 그대로 교부되어야 한다.

7.4.2 임상시험용 의약품의 반납

의뢰자는 임상시험의 중지 및 종료 또는 임상시험 담당자가 계획서에 따라 시험을 실시하지 않는 경우 미사용 임상시험용 의약품을 회수하여 폐기하도록 한다. 이때 임상시험용 의약품 관리자는 시험책임자와 협의 후, 미사용된 임상시험용 의약품을 의뢰자에게 반납하고 반납증을 보존하여야 한다. 반납된 임상시험용 의약품은 의뢰자의 회사 내 규정에 따라 조치되거나 폐기된다.

[8] 대상질환

자궁경부상피이형증 CIN Ⅰ

2006년 ASCCP(American Society for Colposcopy and Cervical Pathology)Consensus Guideline에 따르면, 여성에게 진단된 CIN Ⅰ은 치료 없이 정기적인 검사를 통한 관찰을 실시한다.[5]

[9] 임상시험대상자의 선정기준, 제외기준, 목표한 임상시험대상자의 수 및 그 근거

9.1. 선정기준(Inclusion criteria)

다음의 기준에 모두 해당하는 경우에만 본 임상시험에 참여할 수 있다.

1. 만 20세 이상 49세 이하의 가임 여성
2. 자궁경부상피이형증 CIN I 으로 진단을 받은 자

3 HPV 양성인 자

4. 4. 스크리닝 검사 결과가 WBC: 4 $10^3/\mu L$ 이상, Hemoglobin: 9.0g/dL 이상, Platelet 150 $10^3/\mu L$이상, ANC(Absolute Neutrophil Count): 1,500 10*6/L 이상일 경우
5. AST/ALT: 정상범위 상한치의 2.5배 이내, BUN / Creatinine: 정상범위 상한치의 1.5배 이내인 경우
6. 심전도 검사에서 심장질환을 나타내는 비정상적 소견이나 흉부X-ray 검사에서 활동성 질환이 없는 자
7. 임상시험의 내용을 이해하고 참여에 동의하여 동의서에 자필로 서명한 자

9.2. 제외기준(Exclusion criteria)

다음의 기준에 하나라도 해당하면 본 임상시험에 참여할 수 없다.

1. 자궁경부의 이형증 이외의 다른 장기의 악성 종양이 있는 자
2. 활동성 간질환, 면역관련질환, 또는 중증의 신부전이 있는 자
3. 백혈병, 교원질증, 다발성경화증, 자가면역질환 및 임상적으로 의미 있는 알레르기성 질환(투약을 필요로 하지 않는 경미한 알러지성 질환 제외)
4. 당뇨병으로 진단 받은 자
5. 7일 이내에 면역반응에 영향을 줄 수 있는 약물을 복용한 자(Glucocorticoid 제제, 비타민 제제, 건강식품, 한약 등)
6. 임신이 확인 되었거나 또는 수유 중인 자
7. 타 임상연구에 등록되어 있는 자
8. 기타 시험자가 부적합 하다고 판단한 자

9.3 목표한 임상시험대상자의 수 및 그 근거

9.3.1 목표 임상시험대상자 수

- 총 200명 (시험군 100명, 대조군 100명)
- 기관 배정: 34명 (각 기관 경쟁적 등록)

CIN의 3개월간 자연치유율 31.0% 대비 20% 증가율을 고려하여 군당 80명을 모집한다.

군 구성	용량	임상시험대상자 수	예상 치유율	치유예상 임상시험대상자 수	투약기간/관찰기간
대조군	-	80명	31%	24.8명	4주간 1일 1회 투약 및 8주간 추가 관찰
시험군	1,500mg/day	80명	51%	40.8명	
총		160명			

자궁경부상피이형증(CIN)의 가임여성을 대상으로 폴리감마글루탐산(γ-PGA)을 경구투여 시, 자궁경부상피이형증 자연치유율 대비 치유율의 증가(면역치료 효과)를 확인하고, 안전성을 확인하며, 면역학적 지표 변화 평가를 통한 면역증강 효과를 확인하기 위한 2상 연구로 본 연구에서는 폴리감마글루탐산군의 치유율이 위약군에 비하여 20%이상 증가할 경우 대단위 3상 임상연구의 수행을 결정하는 연구로 위약군 대비 치유율의 20%를 고려할 때 통계적 가설은 다음과 같다.

$H_0 : p_1 \leqq p_2$ vs. $H_1 : p_1 > p_2$

p1: Treatment의 치유율, p2: Control의 치유율
기대하는 치유율: 대조군: 31%, 시험군: 51%
유의수준: 0.05, 검정력: 80%, 단측검정

위 임상시험대상자수 산출 근거에 따라 위의 가설 미 예상 치율을 고려할 때 연구대상자 수는 Single stage 방법을 이용할 경우 군당 80명 총 160명이 요구되며, 20% 중도탈락을 고려할 경우 군당 100명, 총 200명의 임상시험대상자 수가 필요하다.
이외에 Jung(2008) 에 근거한 Two stage optimal design방법을 고려하여 임상시험대상자 수를 산출하였는데, Jung(2008)의 방법은 Simon의 단일군 two stage optimal design을 비교군이 포함된 경우로 확장한 방법으로 이에 근거한 대상자 수는 다음의 표와 같다.[7]

[Jung(2008)의 Two-stage 방법을 적용할 경우 다음과 같은 단계를 수행]

단계	그룹당 대상자수	종료 기준: 두 군의 차이 (Trt 치유 환자수-Con 치유 환자수)	실제 유의수준, 검정력
1	43	+2 명 미만	0.461, 0.800
2	80(43+37명)	+11 명 미만	

즉 1단계에서 각 군에 43명을 배정하고, 1단계 종료 시 치유 환자수의 차이가 +2명 미만이면, 효과 없음으로 판정하여 연구종료하고, 그렇지 않을 경우 추가로 각 군당 37명을 모집하여 진행한다.
최종적으로 각 군당 80명의 대상자가 모집된 경우 두 군에서 치유된 환자 수의 차이가 11명 미만일 경우 효과 없음으로 판단하고, 11명 이상일 경우 효과 있음으로 판정한다.
위 Two stage optimal design방법에 따라 20%의 중도탈락을 고려할 경우 임상시험대상자 수는 1단계에서는 군당 54명씩 총 108명의 임상시험대상자가 필요하며, 2단계까지 추가모집을 진행할 경우 군당 100명씩 총 200명의 임상시험대상자 수가 필요하다. 본 연구에서는 Jung(2008)에 기초하여 연구대상자 수를 설정하고, 2단계 디자인을 고려하기로 한다.

9.3.2 산정 근거

한국인에 대한 자궁경부상피이형증에 대한 유병율, 자연 치유율 등에 대한 기초 정보는 매우 제한적으로 나와 있고[6], 치료 효과에 대한 임상연구가 거의 없는 실정으로 본 연구에서는 자궁경부상피이형증에 대한 폴리감마글루탐산의 효과를 탐색하기 위해 국외의 연구결과를 참고하였다.
자궁경부상피이형증에 에 대한 자연 치유율에 대한 연구는 대부분 6개월 이상의 관찰연구가 대부분인데, 본 연구에서와 같이 3개월 시점에서의 자연치유율은 Barnett et al.(2003)이 제시한 CIN Ⅰ, Ⅱ 대상으로 한 연구결과 위약군에서는 약 31%, 시험약인 Photodynamic therapy (PDT)군에서는 약 33%를 보였으며,[8] Trimble(2005)의 CIN Ⅱ, Ⅲ를 대상으로 한 15주간의 연구결과는 자연치유율이 28%로 나타났다.[9]
6개월의 관찰시점에서 측정된 선행연구로, 대단위 관찰연구중 하나로 1001명의 대상으로 한 코호트연구를 수행한 Bansal et al.(2008)의 연구결과를 참고하였을 때, 이 연구에서는 1001명의 CIN Ⅰ에 해당하는 임상시험대상자를 대상으로 최대 12개월간의 추적관찰을 통해 자연치유율을 확인하였는데, 6개월 시점에서 약 49%의 임상시험대상자가 정상으로 회귀하였음을 보고하였다.[10]
본 연구와 유사한 임상연구 단계로 프로제스테론의 국소적 주입의 효과를 확인하기 위한 2상 임상연구(Hefler et al., 2010)에서 특별한 치료를 수행하지 않는 대조군의 자연치유율은 96명중 38.8%로 나타났다.[11] 위의 연구들의 결과를 종합하여 볼 때 대부분의 자연치유율은 3개월 이내에서 나타나고, 추가적인 3개월 후의 추가 효과는 약 7.8%~18.0%로 나타나는 것으로 요약될 수 있다 따라서 이와 같은 결과를 반영하여 대략적인 3개월 시점에서의 자연 치유율은 약 31.0%로 설정하기로 하였다.
본 연구는 2상 임상연구로 탐색적인 연구로 설정되며, 높은 자연치유율과 관련하여 적어도 20%이상의 효과가 밝혀져야 대규모 임상연구가 가능하리라 판단하였고, 그 이하의 효과가 나타났을 경우 대규모 임상연구는 가치가 없을 것으로 판단하였다.

[10] 임상시험의 기간

IRB 승인일로부터 마지막 임상시험대상자의 시험 종료일까지 약 48개월.

[11] 임상시험의 방법 (투여-사용량, 투여-사용방법, 투여-사용기간, 병용요법등)

본 연구는 자궁경부상피이형증(CIN)의 가임여성을 대상으로 폴리감마글루탐산(γ-PGA)을 경구투여 시, 자궁경부상피이형증 자연치유율 대비 치유율의 증가(면역치료 효과)를 확인하고, 안전성을 확인하며, 면역학적 지표 변화 평가를 통한 면역증강 효과를 확인하기 위한 2상 다기관, 평행, 이중맹검, 위약대조 연구로 설계되었다.
본 연구에는 군당 총 80명(중도탈락 고려 시 100명)의 대상자가 참여되어 있는데, 연구 대상자의 참여는 Jung(2008)이 제시한 2단계에 걸쳐 진행된다. 1단계에서는 각 군당 43명의 연구대상자의 모집되어 주 효과 변수인 치유 여부를 확인하고 치유인 경우의 임상시험대상자 수의 차이가 2명 미만(폴리감마글루탐산의 치유 임상시험대상자수-위약군에서의 치유 임상시험대상자의 수)일 경우 자궁경부상피이형증에 있어 폴리감마글루탐산의 효과는 없는 것을 판단하고 연구를 종료하게 된다. 만약 1단계에서 2명 이상 차이가 나면, 2단계에 진입하여 추가적으로 각 군당 37명을 추가하게 되는 2단계 연구 디자인을 고려하기로 한다. 1, 2단계 모두 합하여 각 군당 80명의 대상자 중에서 군간 치유인 경우의 임상시험대상자 수 차이가 11명 이상 차이가 발생하게 되면 폴리감마글루탐산의 치유효과를 확인하게 되고, 그렇지 않을 경우 폴리감마글루탐산은 자궁경부상피이형증 치유에 효과적인 못한 것으로 판단하게 될 것이다.

11.1 투여 사용량

11.1.1 투여 사용량의 근거

임상1상으로 5일 연속투여 후 5일 관찰한 연구에서 면역학적 지표 변화 확인 결과 위약군에 비해 투약군이 전반적으로 높은 자연살해세포 활성을 나타냈으며, 투약 후 3일과 5일의 경과시점에서 저용량군(1.5g/일)이 고용량군(3.0g/일)보다 높은 자연살해세포 활성을 나타내었다
위의 선행시험에서 중대한 이상반응/이상약물반응의 사례는 없었으며, 1.5g/일과 3.0g/일의 용량 중 면역치료제로서의 기능 상 높은 자연살해세포 활성을 나타내는 1.5g/일 용량으로 투여를 결정 하였다.

11.1.2 투여 사용량

시험군: 폴리감마글루탐산 1,500mg / 100mL
대조군: 위약 / 100mL.

11.2 투여 사용방법

폴리감마글루탐산 또는 위약을 100mL를 1일 1회 경구 복용 한다.
음식물은 이 약의 흡수를 방해하기 때문에 충분한 흡수를 위하여 취침 전에 복용 한다. 정해진 복용일에 복용하는 것을 잊은 경우는 당일에만 복용하고 같은 날 1회 용량 이상을 복용하지 않도록 한다.

11.3 투여 사용기간

11.3.1 투여 사용기간의 근거

임상 1상과 건강인 대상 임상 연구의 결과자료를 근거로 4주의 투여기간으로 폴리감마글루탐산의 임상적 유효성이 나타날 것으로 판단한 기간이며 비설치류 반복투여 독성시험자료와 동일한 기간이

다.

11.3.2 투여 사용기간

4주

11.4 병용약물

11.4.1 병용가능 약물

임상의의 판단 하에 허용하되, 타 질환 또는 이상반응 발현 시 약물투여를 포함한 모든 병용약물의 투여 시 사용한 약물에 대한 약물명, 투여목적, 용량, 투여기간 등의 정보를 증례기록서에 기록 한다.

11.4.2 병용금지 약물

임상시험기간 중 금지해야 할 약물은 다음과 같으며, 병용금기 약물의 사용이 필요한 경우에 해당 임상시험대상자는 임상시험을 중단하여야 한다.

1)스테로이드계 약물: Betametasone, Dexametasone, Prednisolone, Hydrocortisone 등이며 국소적으로 피부나 눈 등에 적용하는 경우는 예외

2)면역조절물질: 성장호르몬, EPO(Erythropoietine), 알부민 제제, 전혈 및 백혈구 성분수혈 등

3)기타: 임상시험용의약품 이외의 건강기능 보조식품 및 비타민 제제, 한약

[12] 관찰항목·임상검사항목 및 관찰검사방법

12.1. 관찰 및 임상검사항목

관찰 및 검사항목과 실시일정은 다음의 임상시험 진행일정표를 따른다.

Visit Type	Screening	Baseline &Treatment	Observation	Observation	Closing
Visit No.	1	2	3	4	5
Visit Week	-4주 이내	0주	4주 ± 5일	8주 ± 5일	12주 ± 5일
서면 동의	V				
기초정보 조사	V				
선정/제외기준 검토	V				
과거력[1]	V				
신체검사/흉부X선 검사	V				
활력징후	V	V	V	V	V
실험실 검사[2]	V		V	V	V
임신반응 검사[3], HbA1c[4]	V				
ECG	V				V
Reid Colposcopic Index[6]	V				V
Colposcopy with Biopsy[5]	V				V
Pap Smear Test[6]	V				V
HPV DNA Chip Test[6]	V				V
HPV HC Ⅱ[6]	V				V
NK Cell Activity[6]		V	V	V	V
PBMCs 면역표현형 검사[6]		V	V	V	V
배정번호 부여		V			
시험약품교부		V			
복용 순응도			V		
이상반응			V	V	V
병용약물			V	V	V

1. 과거력
   1) 병력 및 수술력: 스크리닝 방문 기준으로 관련 질환은 2년 이내, 기타 질환은 6개월 이내
   2) 복용약물: 스크리닝 방문 기준으로 4주 이내에 투여하였거나 현재 투여 중인 약제
2. 실험실적 검사 항목
   1) 혈액학적 검사: Hct, Hb, WBC(diff. count포함), RBC, Platelet, ANC
   2) 혈액응고 검사: aPTT, PT
   3) 일반화학 검사: Glucose, AST, ALT, Alkaline phosphatase, Total Protein, Albumin Total Bilirubin, γ-GTP, Cholesterol (Total, LDL, HDL), Triglyceride, BUN, Creatinine, Uric acid, Sodium, Calcium, Potassium
   4) 뇨검사: PH, Nitrite, Ketone, Specific gravity, Glucose, Protein, Urobilinogen, RBC
3. 임신반응 검사: Urine hCG
4. 당뇨병 검사: 당화혈색소 HbA1c
5. 1차 유효평가변수: Cloposcopic Biopsy (생검)
   동의취득 전 4주 이내의 검사결과는 스크리닝 검사로 인정.
   (단, 타 기관의 검사결과를 사용 시 최소한 결과를 확인할 수 있는 조직검사 결과지가 확보되어야 함.)

6. 2차 유효평가 변수:
   자궁경부 상피이형증 평가
   1) Reid Colposcopic Index
   2) Pap smear test (세포진 검사)
   3) HPV DNA Chip test(감염 Type 및 소실 검사)
   4) HPV Hybrid Capture Ⅱ assay(viral load 정량검사)

   면역기능 평가
   1) 자연살해세포(NK cell) activity의 측정
   2) 말초혈액 단핵세포의 표현형 변화(MHC class II) CD8, CD56 population의 측정

12.2. 관찰검사방법

12.2.1 임상시험대상자 동의서 취득

본 임상시험을 실시하기에 앞서, 연구자는 다음 사항에 관한 내용을 임상시험대상자 본인에게 설명한다. 임상시험대상자가 내용을 잘 이해한 것을 확인한 다음, 본인의 자유의사에 따른 임상시험 참가의 동의는 서명자 자필로 서명과 날짜를 기록한 문서로 받는다. 또한 동의를 취득한 연월일을 증례기록서에 기입한다.

1) 시험의 목적 및 방법
2) 예상되는 이상반응
3) 임상시험대상자가 시험 참가를 동의하지 않아도 불이익을 당하지 않는다는 점

4) 임상시험대상자가 시험 참가에 동의해도 언제든지 이를 철회할 수 있다는 점
5) 기타, 임상시험대상자의 인권보호 및 시험약의 이해에 필요한 사항

12.2.2 인구학적, 병력, 복용약물 조사 및 신체 검사

임상시험에 들어가기 전 임상시험대상자 인구학적 조사, 병력 등에 대하여 면담, 차트 확인 및 질문 등을 통하여 다음 사항 등을 점검하고 증례기록서에 기록한다.

1) 인구학적 조사: 임상시험대상자 이니셜, 성별, 연령
2) 병력 조사: 현재 앓고 있는 질환 유무, 과거 약물 급성반응 유무, 면역계 질환 유무, 특수 체질 유무 등
3) 복용약물 조사: 과거 4주 이내(현재 복용 중 약물 포함) 복용한 약물 조사
4) 기왕력 조사: 기타 질병 기왕력(2년 이내 ~ 현재는 치료된 경우)
5) 신체검사: 현재의 신체 상태에 대한 검진

12.2.3 활력징후(vital signs)

12.1항의 진행일정표에 따라 혈압 및 맥박수를 측정한다. 혈압은 안정된 상태에서 5분간 휴식 후 측정한다. 앉은 자세에서 온몸에 힘을 빼고 호흡을 편안하게 한 후 표준혈압계로 측정한다.

12.2.4 임상 실험실적 검사

12.1항의 진행일정표에 따라 검사를 수행한다.
검체 채취 및 기록: 채혈은 검사일정에 따라 무균적으로 시행하며, 채혈된 혈액의 검사는 상용화된 혈액학적, 혈액화학적 검사방법에 따라 정도관리 인증된 임상병리검사실에서 시행한다. 검사 결과를 증례기록서에 기록하고 비정상적인 수치에 대해서는 연구자의 의견을 기록한다.
검사항목:

1) 혈액학적 검사: Hct, Hb, WBC(diff. count 포함), RBC, Platelet, ANC
2) 혈액응고 검사: aPTT, PT
3) 일반화학 검사: Glucose, AST, ALT, Alkaline phosphatase, Total Protein, Albumin, Total Bilirubin, γ-GTP, Cholesterol (Total, LDL, HDL), Triglyceride, BUN, Creatinine, Uric acid, Sodium, Calcium, Potassium
4) 뇨검사: PH, Nitrite, Ketone, Specific gravity, Glucose, Protein, Urobilinogen, RBC
5) 임신반응 검사: Urine hCG
6) 당뇨병 검사: HbA1c(당화혈색소)

12.2.5 심전도(ECG)

12.1항의 진행일정표에 따라 12-lead ECG를 수행한다. 기본적인 기록 이외에 automatic analysis & recording이 출력되는 항목인 ventricular rate (beats/min), PR interval (msec), QRS (msec), QT/QTc (msec)를 CRF에 기록한다.

12.2.6 흉부X-선 검사

흉부 폐질환이나 병소 등 소견 상의 유의할 만한 이상이 있는지 확인하기 위해 흉부 X-선 촬

영을 실시한다.

12.2.7 자궁경부상피이형증 진단 검사[12-15]

12.1항의 진행일정표에 따라 검사를 수행한다.

1) Colposcopic Biopsy (생검)

질 확대경하 조직 생검을 시행한 결과는 World Health Organization (WHO)의 CIN 체계에 의거하여 Normal, CIN I, CIN II, CIN III, 그리고 SCC로 분류하며, 만성 염증 소견은 정상으로 분류 한다. 본 임상시험은 질 확대경하 조직 생검을 시행한 결과로 CIN I을 대상으로 선정한다.

< Colposcopy에 대한 표준화된 방법 >

1. 질확대경 검사 시 환자는 쇄석위(lithotomy position)로 위치시키고 회음부의 이상부위를 확인한 후 질경을 삽입하여 자궁경부가 노출되면 질확대경을 통하여 먼저 자궁경부를 자세히 관찰한다.

2. 시야를 방해하는 질 분비물은 조심스럽게 제거하며 먼저 정상 생리 식염수를 도포하여 5~10배율로 전체적인 모습, 백반증 (leukoplakia), 혈관상태를 자세히 관찰한다. 이때 녹색 필터 (green filter)를 사용하면 혈관의 붉은색을 여과시켜 검은 색으로 보이게 하므로 혈관의 모습을 더 잘 관찰할 수 있다.

3. 생리 식염수에 의한 검사 후에는 초산 용액에 의한 상피의 변화를 관찰하게 되며, 3~5% 초산 용액으로 적신 면구나 면봉을 이용하여 자궁경부를 도포한다. 처음 초산 용액을 도포한 후 약 30초~1분 후에 관찰하며, 초산 용액을 한 번 더 도포하면 첫 번째 도포로 시작된 초산용액의 반응이 증가한다.

4. 초산 용액 도포 후 편평 세포 접합부(junction of squamous cell)를 확인하고 미란(erosion), 초산 백색 상피(aceto-white areas)여부와 혈관모양을 관찰한다. 일반적으로 5~16배의 배율에서 초산 백색상피를 분명히 관찰할 수 있다.

5. 초산 용액의 도포에 의한 검사가 끝나면 25~50%의 Lugol 요오드 용액을 도포한 다음 모든 부위를 다시 관찰해야 한다. 정상 편평상피는 glycogen을 포함하고 있어서 요오드 용액에 의해 짙은 갈색 iodine positive으로 보이며 병변은 요오드에 반응하지 않는다.

6. 질확대경하 생검은 가장 심한 이상이 보이는 부위들 중에서 2군데를 생검 하는 것을 원칙으로 하나 병변이 작아서 2군데의 생검이 어려운 경우는 1군데만 하고, 병변이 넓어서 2군데로 정확도가 떨어진다고 판단하는 경우는 3군데까지 허용하여 생검 집게(biopsy forceps)를 사용하여 조직을 채취한다. 더 많은 부위의 생검은 병변의 제거 목적이 될 수 있으므로 허용하지 않는다.
7. 생검 부위로부터 출혈은 대개는 스스로 멈추지만 즉시 지혈시키거나 지연 출혈을 막기 위해 농축된 Monsel 용액을 사용할 수 있다.

8. Reid’s index 및 사진을 촬영하여 기록으로 증명 한다.
질확대경 상 관찰된 소견의 촬영은 자궁경부 외구와 이행대를 중심으로 병변을 모두 포함 할 수 있

도록 하며 초산 용액과 요오드 용액을 사용 후 변한 것을 찍어서 보관 한다. 이 사진을 보고 객관적으로 Reid's index의 점수를 줄 수 있어야 하며, 병변이나 자궁경부가 한 장의 사진에 들어 오지 않는 경우는 2장으로 나누어서 찍는다.

<비정상 질확대경 소견>

백색 상피 (white epithelium): 초산 점적 후 경계가 명확한 백색의 병변이 나타나는 것으로 육안적으로 나타나는 백반 (leukoplakia)과는 다르다. 상피세포의 증식이 왕성하여 핵의 밀도가 증가하여 일어나는 현상으로 자궁경부 상피내종양 (CIN)에서 흔히 보는 소견이다.	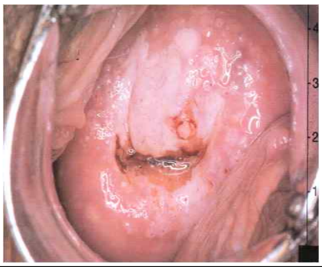
적점반 (punctation): 국소 상피의 이상으로 모세혈관이 명확한 경계를 가진 점상으로 나타나는 것으로 자궁경부 상피내종양 (CIN)에서 흔히 보는 소견이다.	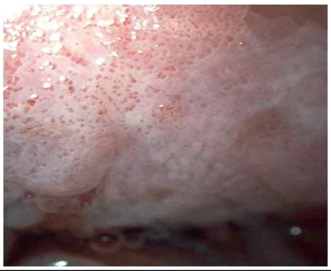
모자이크 (mosaic) : 표면 상피 사이로 바구니 모양의 혈관망을 형성하여 모자이크 형상을 나타내는 것을 말하며 경도의 이형 증 시는 모양이 규칙적이나 병변이 진행됨에 따라 불규칙 하 게 된다. 주로 CIN I 이상의 병변과 관련 있다.	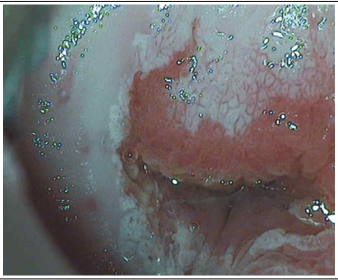
백반 (leukoplakia) : 국소 상피에 조직학적으로 과각화 (hyperkeratosis)되어 생기는 것으로 초산 점적과 무관하며 편평하게 융기된 백색병변을 말한다. 정상 편평 상피에 산재되 어 있는 경우는 임상적으로 별 의의가 없으나, 변형대에 국소 적으로 있는 경우 전암병소를 의심하여야 한다.	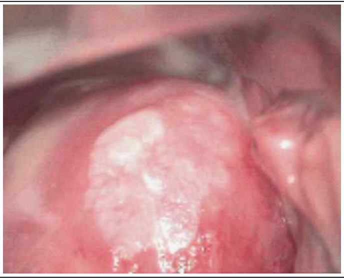
비정상 혈관 (atypical vessel) : 말초 모세혈관의 모양, 직경, 주 행 상태, 혈관사이의 간격 등이 정상 편평상피의 혈관과 달리 불규칙성을 가진 것을 말하며, 대부분의 경우 침윤암의 위험성 을 가진다.	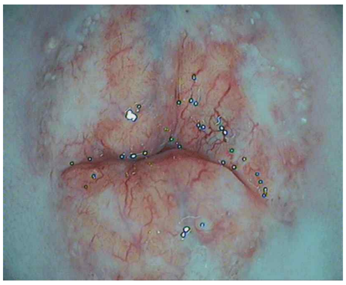

<현미경 관찰을 위한 조직표본 제작법>

1. 질확대경하 생검을 통해 채취된 조직은 포름알데하이드(formaldehyde, HCHO) 넣어 고정(fixation)을 시킨다.
2. 고정시킨 조직은 파라핀을 이용하여 포매(Embedding)시킨다.
3. 파라핀 블록은 현미경 관찰을 위해 ㎛단위의 얇은 조직절편으로 박절(Section)한다.
4. 조직절편은 헤마톡실린-에오신(Hematoxylin-Eosin) 염색을 하여 현미경으로 관찰한다.

<자궁경부상피내종양(CIN, cervical intraepithelial neoplasia) 진단>

CIN I	mild dysplasia: 상피층의 하 1/3이 미성숙 미분화세포로 대치됨
CIN II	moderate dysplasia: 상피층의 중간 1/3까지 미성숙 미분화세포로 대치됨
CIN III	severe dyaplasia: 상피층의 상 1/3까지 포함되었으나 표면에는 아직 성숙세포가 존재
	상피내암(carcinoma in situ): 구별할 수 없는 미분화된 이상세포로 전상피세포층이 대치됨

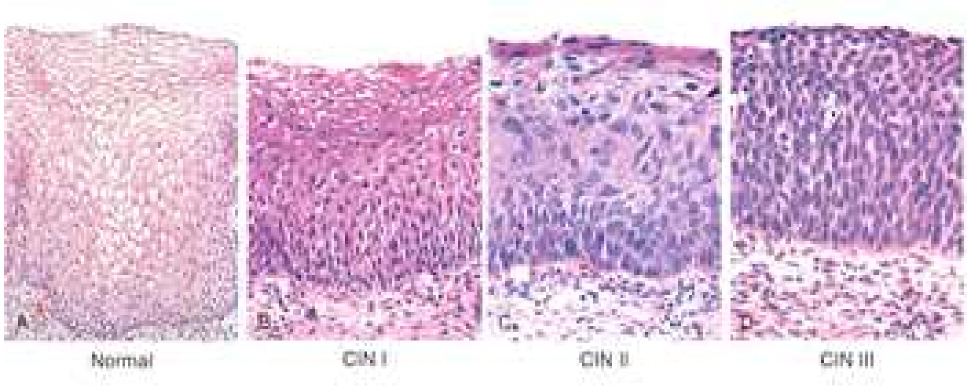

2) Reid Colposcopic Index

CIN 병변의 심한 정도(severity)를 예측하는데 있어서 객관적이고, 정확하며, 재현 가능한 지표를 만들기 위해 여러 colposcopy 소견들을 그 특징에 따라 분류하고 점수화한 grading system으로 margin of the lesion, color of the acetowhitening, type of vascular pattern, iodine staining reaction 등 네 가지 colposcopic criteria를 사용 한다[16].

**Reid Colposcopic Index**						
Colposcopic Sign	Zero Point (0)	Check	One Point (1)	Check	Two Points (2)	Check
**Margin**	Exophytic condylomas;areas showing a micropapillary contour. Lesions with distinct edges. Feeatheard, scalloped		Lesions with a regular(circular) shape, showing smooth, straight edges.		Rolled, peeling edges. Any internal demarcation between areas of differing colposcopic appearance.	
**Color**	Shiny, snow-white color. Areas of faint (semi-transparent) whitening.		Intermediate shade (shiny, but gray-white).		Dull reflectance with oyster-white color.	
**Vessels**	Fine-caliber vessels, poorly formed patterns.		No surface vessels.		Definite, coarse punctation or mosaic.	
**Iodine**	Any lesion staining mahogany brown; mustard-yellow staining by a minor lesion (by first three criteria).		Partial iodine staining (mottled pattern).		Mustard-yellow staining of a significant lesion (an acetowhite area scoring 3 or more points by the first three criteria).	

점수는 0에서 8점까지 채점되며 점수에 따른 Grading은 아래와 같이 판정된다.
0-2점: Likely to be CIN I
3-4점: Overlapping lesion - likely to be CIN I or CIN II
5-8점: Likely to be CIN II

3) Pap smear(Liqid Based cytology, 세포진 검사)
자궁경부 세포검사는 자궁내경관에 brush(broom)의 긴 중앙부분을 넣어 동일한 방향으로 2~3회 회전 후 보존액이 담겨진 용기 안에 brush를 분리시켜 넣고 뚜껑을 닫는다.

검사결과는 Negative, ASC-US, ASC-H, LSIL, HSIL, Invasive cancer(Cervical carcinoma)로 판독 한다.

4) HPV DNA Chip test

자궁경부 세포검사 시 자궁내구와 자궁외구 cytobrush를 이용하여 채취한 검체를 사용한다. 종양상피세포와 HPV 감염 여부 및 virus type의 상관관계를 확인하기 위하여 방문1, 방문5에서 실시한다. HPV의 양성과 음성으로 분류하며 양성인 경우 HPV 16, 18, 31 ,33, 35 ,39, 45, 51, 52 등의 HPV type을 기록한다.

5) HPV Hybrid Capture Ⅱ assay(viral load, 정량검사)

HPV viral load의 측정은 Hybrid captureⅡ system을 이용하여 Collection kit내 용액을 원심분리하여 상충액을 분리하고 denaturation reagent 와 섞은 후 65C에서 45분간 배양한 다음 13가지 type의 고/중증도 위험군 인두유종 바이러스의 RNA probe가 들어 있는 용액과 섞어 60C에서 60분간 배양하여 RNA/DNA hybrid에 대한 단일 클론항체를 표면에 부착 시킨 시험관에 넣어 항체에 capture 시킨 후, 이렇게 결합된 hybrid에 이차면역항체와 화학발광물질인 Lumiphos530을 첨가하여 15분간 배양 alkaline phosphatase 에 의해 기질이 분해되어 발산되는 빛을 luminometer를 이용하여 측정한다. 시료의 빛의 단위는 HPV -16,-18의 DNA가 10 pg/mL로 들어있는 양성 대조군으로 잡아 양성군에 대한 상대적인 밝기 RLU(Related light unit로 정하고, 이 비율이 1.0 이상 이면 HPV 양성, 1.0 미만이면 HPV 음성으로 판단한다. 이는 상피내종양과 HPV virus와의 상관관계를 알아보기 위해 실시한다.

조직검사는 각 기관의 정도 관리가 된 병리실에서 시행하며 표준화된 방법 조직검사 기준에 따라 실시하게 된다. Pap smear test, HPV test는 각 병원 간의 분석법이나 절차에 따라 결과의 차이가 있을 수 있음을 고려하여 지정된 임상시험 실시기관의 검사실에서 통합하여 실시한다.
각 기관에서의 검체 운송 관리는 본 임상시험 검체관리지침에 따라 (주)바이오리더스의 담당자가 직접 수거 및 운송하여 연구시설에 배송 할 것이다.

12.2.8 선정/제외기준 검토

방문1의 Screening 시기에 실시된 배경조사, 병력조사, 복용약물조사, 신체 검사, ECG, 활력징후, 임상 실험실적 검사 결과를 확인하며 Biopsy의 조직학적 검사 결과 CIN I으로 진단받은 자, HPV DNA test 결과 인두유종 바이러스가 양성으로 나온 자로 임상시험대상자 선정 및 제외 기준에 적합한지 평가한다.

12.2.9 면역학적 지표 평가

HPV감염에서 혈청학적 및 세포면역체계(Serologic, cellular immune system)의 세포 매개 면역반응은 감염된 상태 및 감염과 연관된 질병인 자궁경부상피이형증의 소실에 중요하다. HPV에 이미 감염된 사람을 통하여 세포 매개 면역반응을 유도함으로써 HPV 감염 및 관련 병변의 진행을 조절 또는 억제 시킬 수 있다
12.1항의 진행일정표에 따라 면역학적 지표 평가 검사를 위한 채혈을 수행한다.

<라벨링>
각각의 혈장 튜브에는 미리 제공된 라벨을 사용하여 부착한다. 라벨 위에 필요한 정보를 유성잉크를 사용하여 기재하도록 하며, 기재 시 필수 기록 항목은 임상시험대상자 배정번호, 이니셜, 혈액채취 일시로 한다.
<검사 시기>
본 연구에서 수집된 모든 혈장 샘플에 대하여 검사는 일괄 실시함을 원칙으로 한다.
<검사 방법>
샘플의 전처리와 정량은 사전 validation된 방법에 따라 수행되어야 하며, 그 방법의 신뢰성을 분석실시기관의 장과 임상시험 의뢰자가 보증하여야 한다.
<검사기관 및 검사자>
분석은 대한 임상병리정도관리협회로부터 인증서를 발급 받은 임상시험실시기관의 해당 부서(진단검사의학과)나 또는 이에 준하는 수준으로 본 검사법이 잘 확립된 연구시설에서 실시하며, 검사자는 본 검사에 숙련된 자로서 해당 기관의 표준작업지침(SOP)에 따라 실시한다. 검사자의 인적사항과 시험 결과의 원 자료(Raw data)에 대한 사후 점검이 가능하도록 문서화 하여야 한다.

1) 자연살해세포(NK cell activity)의 측정
최근의 CD107a marker를 이용한 NK 활성 측정 방법으로 변경 Ex vivo test로서, 임상시험대상자로부터 채혈된 말초혈액에서 먼저, 단핵구를 분리한다. 자연살해(NK, natural killer)세포의 활성을 측정하기 위하여 K562세포를 자연살해 세포의 표적세포로 사용한다. 표적세포를 FACS tube에 4X105cell/mL 농도로 100μL 접종한 후 말초혈액 단핵세포를 표적세포와 10:1의 비율이 되도록 FACS tube에 첨가한다. 자연살해 세포 활성은 표적세포인 K562 세포를 사멸시킬 때 자연살해 세포 표면에 나타나는 단백질 표지를 측정하여 계산한다.

2) 말초혈액 단핵세포의 표현형 변화(MHC class II CD8, CD56 population)의 측정
임상시험대상자의 말초혈액을 채혈한 뒤 ficoll-hypaque의 비등차 용액을 이용한 density gradient법으로 말초혈액 단핵 세포(PBMNCs)를 분리한다. 약술하면 SEPMATE tube (stemcell, 15450) 를 이용하여 혈액시료를 Histopaque (비중 1.077 g/ml sterile-filtered, Sigma)용액 위에 중첩시키고 1200Xg에서 10분간 원심분리한 후 상층을 분리하여 PBS+2% FBS 배지로 2회 세척하여 단핵구르 분리한다.
Ex vivo test로서 유세포 분석기(BD-true counter이용)를 통하여 말초 혈액 단핵세포의 면역 표현형 변화를 확인하기 위하여 임상시험대상자의 말초 혈액 단핵구(PBMNCs)를 FACS buffer (0.1% Bovine serum albumin, 0.01% sodium azide, PBS)으로 2회 세척한 다음
FACS buffer 50 ul 에 여러가지 형광이 결합된 특이 항체 (CD3-Percp-eFluor 710, CD8-FITC, CD56-PE, CD14-APC-eFluor 780, CD19-APC-eFluor 780, CD107a-AlexaFluor647) 를 첨가하여 얼음에서 30분간 방치한다.
형광 염색된 세포를 FACS buffer 로 2회 세척한 후 2% parafomaldehyde 가 포함된 인산 완충액으로 고정시킨 다음, 세포의 형광을 유세포 측정기(Gallios, Beckman, USA)를 이용하여 측정하고 분석한다.

각 기관에서의 검체 운송 관리는 본 임상시험 검체관리지침에 따라 (주)바이오리더스의 담당자

가 직접 수거 및 운송하여 연구시설에 배송 할 것이다.

12.2.10 확률화 배정

임상시험대상자는 연구자에 의해 순차적으로 배정번호를 부여 받는다(Screening No.의 순서와 무관함). 무작위 배정코드는 통계학자 또는 확률화 배정 담당자가 컴퓨터 발생의 고유의 방법으로 배정코드를 무작위로 발생시키는데 본 연구에서는 적절한 블록크기의 블록 확률화 배정 방법을 사용하여 각 기관별로 배정하기로 한다. 확률화 배정은 순서대로 진행되어야 하며, 이미 한 번 배정된 이후에는 임상시험대상자가 동의를 철회했다하더라도 다른 임상시험대상자에게 해당 무작위 배정코드를 재부여할 수 없다.

12.2.11 임상시험용 의약품 처방 및 교부

시험기관의 시험책임자는 임상시험용 의약품의 관리에 대한 책임이 있으며, 임상시험용 의약품 관리는 임상약국의 관리약사가 본 임상시험에서 사용되는 임상시험용 의약품을 임상시험이외의 목적으로 사용되지 않도록 보관, 관리해야 한다.

의뢰자는 임상시험 실시 중 임상시험용 의약품의 수량 및 보관상태 등을 확인하고 임상시험이 적절히 진행될 수 있도록 조치하여야 한다. 시험책임자는 의뢰자로부터 배송 된 임상시험용 의약품이 관리약사에 의해 정확히 수령되고, 권장 보관 조건에서 안전한 장소에 보관됨을 확인해야 하며 임상시험용 의약품이 교부 전에 손상되지 않도록 보존할 책임이 있다.

각 개별 임상시험대상자 포장은 시험기관에서 추가 재 포장 또는 라벨 부착 없이 의뢰사에서 제공한 그대로 교부되어야 한다.

12.2.12 시험약 반납 및 복용 순응도 평가

의뢰자는 임상시험의 중지 및 종료 또는 임상시험 담당자가 계획서에 따라 시험을 실시하지 않는 경우 임상시험용 의약품 을 회수하여 폐기하도록 한다. 이때 임상시험용 의약품 관리자는 시험책임자와 협의 후, 미 사용된 임상시험용 의약품과 임상시험대상자의 사용 후 반납된 임상시험용 의약품을 의뢰자에게 반납하고 반납증을 보존하여야 한다. 반납된 임상시험용 의약품은 의뢰사의 회사 내부 규정에 따라 조치되거나 폐기된다.

임상시험용 의약품의 복약 순응도 확인

임상시험대상자는 방문2(0주)에서 수령하였던 임상시험용 의약품을 복용한 빈 용기와 미복용한 약물, 기록된 약물일지를 모두 지참하여 반납하며, 연구코디네이터는 방문2에서 처방된 약품의 수량 및 임상시험대상자가 복용한 임상시험용 의약품의 수량을 파악하여 복약순응도를 아래와 같이 계산 후 증례기록지에 기록한다. 임상시험용 의약품의 복약 순응도는 80% 이상의 경우를 "순응"으로 간주한다.

복약 순응도(%) = 복용한 임상시험용 의약품의 개수/복용해야 할 임상시험용 의약품의 개수 X 100

12.2.13 이상반응 평가

이상반응은 투약이 이루어진 시점부터 임상시험 종료 시까지 면밀히 관찰하며, 방문 시에 시험담당자의 면담 및 문진 등 진료를 통하여 확인한다. 이상반응의 발생 시에는 발현일 및 소실일, 이상반응의 정도 및 결과, 임상시험용 의약품과 관련하여 취해진 조치 및 임상시험용 의약품과의 인과관계, 시험약 이외 의심되는 약제명, 이상반응에 대한 치료 여부 및 내용 등을 상세히 기록한다. 방문 후에도 이상반응의 발생 시 즉시 연구자에게 보고하고, 내원하여 진료를 받을 수 있도록 임상시험대상자를 교육하며, 임상시험대상자에게 연구자의 연락처를 제공하여야 한다. 세부적인 이상반응의 평가와 기록 및 처치는 **16.3**항에 따른다.

12.2.14 병용약물 조사

임상시험 기간 동안 임상시험용 의약품 이외의 약물은 투여하지 않는 것을 원칙으로 하므로, 이에 대한 충분한 임상시험대상자 교육을 한다. 또한 불가피하게 약물 복용이 필요한 경우 연구자에게 연락하도록 교육한다.

시험책임자의 판단 없이 투약한 약물이 본 임상시험의 유효성 및 안전성 평가에 영향을 줄 수 있다고 예상되는 경우 이 임상시험대상자는 탈락하게 된다. 투여한 모든 약물 및 투여사유는 임상시험대상자에 대한 문진 및 챠트 기록을 통하여 조사하여 증례기록서에 기록한다. 본 임상시험에서의 병용금지 약물은 다음과 같다.

1)스테로이드계 약물: Betametasone, Dexametasone, Prednisolone, Hydrocortisone 등이며 국소적으로 피부나 눈 등에 적용하는 경우는 예외
2)면역조절물질: 성장호르몬, EPO(Erythropoietine), 알부민 제제, 전혈 및 백혈구 성분수혈 등
3)기타: 임상시험용의약품 이외의 건강기능 보조식품 및 비타민 제제, 한약

12.2.15 자궁경부상피이형증 진행여부 확인

방문2(0주)에서 방문3(4주±5일)까지의 임상시험용 의약품을 투여 완료한 이후 8주간의 관찰을 실시한다. 방문5(12주±5일)의 최종방문 시에는 Colposcopic Biopsy를 포함한 자궁경부상피이형증의 유효평가검사를 실시하며 Colposcopic Biopsy 상으로 progression이 된 경우는 단계에 맞는 표준화된 치료법을 실시할 수 있도록 안내하며 임상시험대상자의 임상시험을 종료 한다.

12.3. 방문일정 개요

임상시험대상자의 모든 방문은 사전 예약된 일정에 따라 이루어져야 하며, 각 방문의 Visit Window를 엄격하게 지켜야 한다.

각 방문의 Visit Window는 방문1은 -4주 이내, 방문2는 Baseline으로 0주, 방문3은 4주±5일, 방문4는 8주±5일, 방문5는 12주±5일로 임상시험대상자의 참여 기간은 총 12주 이다.

12.3.1. 방문1(-4주 이내, Screening)

본 연구에 대한 설명을 듣고 임상시험에 참여 하기를 원하면, 임상시험대상자 서면 동의서를 작성 후 아래에 기술된 스크리닝 절차 및 검사를 시행한다. 임상적으로 유의한 이상이 있는 자는 임상시험에서 제외한다.

1) 임상시험대상자 서면 동의서
2) 인구학적 정보
3) 병력조사(관련질환 2년 이내, 기타 질환 6개월 이내), 복용약물 조사(4주 이내)
4) 활력징후, 이학적 검사, 신체계측(체중, 키)
5) 실험실 검사, 임신반응 검사
6) 심전도 검사
7) 흉부 X선 검사
8) Colposcopy(Reid's Index, Biopsy)
동의취득 전 4주 이내의 검사결과는 스크리닝 검사로 인정.
(단, 타 기관의 검사결과를 사용 시 최소한 결과를 확인할 수 있는 조직검사 결과지가 확보되어야 함.)
9) Pap smear 검사, HPV DNA test, Hybrid Capture Ⅱ assay 검사
10) 임상시험대상자 적합성 평가(선정/제외기준)

선별일지(Subject Screening/Enrollment Log)

선별일지에 임상시험대상자 동의서에 서명한 임상시험대상자의 목록을 기록한다. 만약 임상시험대상자가 자격이 없거나 다른 이유로 인해 이 시험에 참가할 수 없다면 그 이유가 기록되어야 한다.

12.3.2. 방문2(0주 Baseline & Treatment)

1) 임상시험대상자 적합성 최종 평가
2) 활력징후 측정
3) 복용약물 변화 확인
4) 배정번호 부여
5) 임상시험용 의약품 처방 및 교부
6) NK cell activity 및 면역표현형 검사

선정/제외기준 검토

임상시험대상자의 활력징후, 임상적인 검사 결과 등을 통해 선정/제외기준을 검토하여 최종적인 임상시험대상자의 적합성 평가로 선정여부를 결정한다.

12.3.3. 방문3(4주±5일, Observation)

1) 활력징후 측정
2) 병용약물 확인
3) 임상시험용 의약품 반납 및 복약 순응도 평가
4) 이상반응 확인
5) NK cell activity 및 면역표현형 검사
6) 실험실 검사

12.3.4. 방문4(8주±5일, Observation)

1) 활력징후 측정
2)이상반응 확인
3)병용약물 확인
4) NK cell activity 및 면역표현형 검사
5) 실험실 검사

12.3.5 방문5(12주±5주, Closing)

1) 활력징후 측정
2) 이상반응 확인
3) 병용약물 확인
4) 실험실 검사
5) ECG 검사
6) Colposcopic Biopsy 검사
7) Colposcopy(Reid's Index), Pap smear 검사
8) HPV DNA test, Hybrid Capture Ⅱ 검사
9) NK cell activity, 면역표현형 검사

자궁경부상피이형증의 치유 혹은 진행을 확인하며, 임상시험대상자의 자궁경부상피이형증의 상태에 따라 충분한 의학적인 설명과 지속적인 추후 관찰이나 표준화된 치료법의 실시 등을 안내하며 임상시험대상자의 임상시험을 종료 한다.

[13] 예측 부작용 및 사용상의 주의사항

13.1 예측 부작용

폴리감마글루탐산의 선행된 임상시험에서 유의할 만한 유해사례가 관찰되지 않았으므로 예측되는 부작용은 없으나 폴리감마글루탐산의 특성 상 점성이 있는 시럽의 제제로 개인의 취향에 따라 복용 시 불편감을 호소할 수도 있을 것으로 예측된다.

13.2 사용상의 주의사항

신중한 투여: 현재까지 특이 부작용이 보고되지 않았으므로 정해진 용법에 따라 정량 복용을 준수한다. 임상시험대상자에게 임상시험 기간 중 불편감이나 이상증상 등이 나타나면 연구자에게 보고하도록 하고 연구자는 이상반응의 발생여부를 확인하여 적절한 조치를 한다.

[14] 시험중지·탈락기준 및 분석제외 기준

14.1 시험중지·탈락기준

임상시험에 참여한 임상시험대상자의 임상시험 중지 및 탈락이 될 수 있는 해당 사항은 다음과 같다.

1) 임상시험용 의약품에 대하여 급성반응(알러지, 과민반응 등)을 보이는 경우
2) "중대한 이상반응"의 발생으로 지속적인 참여가 적절하지 않다고 연구자가 판단하는 경우
3) 선정/제외기준의 위반 또는 중대한 임상시험 계획서의 위반이 발생된 경우
4) 임상시험대상자가 임상시험용 의약품의 투여중단을 요구하거나, 임상시험 참여 동의를 철회하는 경우
5) 임상시험대상자의 불참으로 지속적인 관찰이 불가한 경우
6) 기타 연구자가 시험을 중지하여야 한다고 판단한 경우

임상시험대상자가 임상시험에서 탈락 되는 경우, 임상시험용 의약품의 투여를 중단하고, 탈락 시점까지 획득한 시험의 모든 결과와 함께 중단 날짜와 사유 및 소견을 증례기록서에 기록하여야 한다. 임상적으로 유의한 실험실적 이상의 결과로 임상시험에서 제외하는 경우 해당사건이나 실험실적 이상이 해결될 때까지 또는 합리적인 기간이라고 결정한 기간 동안 추적조사를 한다.

14.2 분석 제외기준

중대한 임상시험계획서 위반의 경우, 해당 임상시험대상자를 PP분석에서 제외함을 원칙으로 하며 해당사항은 다음과 같다.

1) 임상시험대상자의 임상시험용 의약품 복용 순응도가 80%미만인 경우
2) 시험 도중 선정기준 및 제외기준에 위반되는 사항이 발생한 경우
3) 유효성 평가에 영향을 줄 수 있는 약물을 병용한 경우
4) 유효성 평가항목 검사 (방문1)를 실시하지 않은 경우
5) 방문예정일을 어겨 방문한 경우

기타, 연구결과 해석에 영향을 미치지 않을 것으로 판단되는 경미한 임상시험계획서의 위반사항은 위반 또는 지연 정도와 사유를 정확히 기재하고 결과보고서 작성 시 연구자, 의뢰자, 모니터요원, 통계학자가 임상시험에 영향을 주었는지 종합적으로 고찰하여 PP분석에 포함하도록 한다.

[15] 효과 평가기준, 평가방법 및 해석방법(통계분석방법)

15.1 효과평가기준

임상시험 결과를 평가하기 위한 1차 및 2차 유효성 평가변수는 아래와 같으며 각각의 평가변수에 대하여 유효성이 분석 될 것이다.

15.1.1 1차 유효성 평가변수

약물 투여 전 Baseline(-4주 이내) 대비 약물 투여 후 12주째 치유율의 시험군과 대조군의 비교
치유의 정의는 CIN I 의 단계에서 Normal로 된 경우를 의미 한다.

15.1.2 2차 유효성 평가변수

약물 투여 전 Baseline(-4주 이내) 대비 약물 투여 후 12주째 Reid Colposcopic Index 비교
약물 투여 전 Baseline(-4주 이내) 대비 약물 투여 후 12주째 Pap Smear Test 결과 비교
약물 투여 전 Baseline(-4주 이내) 대비 약물 투여 후 12주째 HPV DNA Chip Test 결과 비교
약물 투여 전 Baseline(-4주 이내) 대비 약물 투여 후 12주째 HPV HC Ⅱ assay 결과 비교
약물 투여 전 Baseline(0주) 대비 약물 투여 후 4주, 8주 및 12주째 자연살해세포(NK cell) activity 의 결과 비교
약물 투여 전 Baseline(0주) 대비 약물 투여 후 4주, 8주 및 12주째 말초혈액 단핵세포의 표현형 변화(MHC class II) CD8, CD56 population의 결과 비교

15.2 평가방법

15.2.1 1차 유효성 평가변수

약물 투여 전 Baseline(-4주 이내) 대비 약물 투여 후 12주째 치유율의 시험군과 대조군의 비교

약물 투여 전 Baseline 대비 약물 투여 후 Colposcopic biopsy 결과인 자궁경부상피이형증의 단계로 방문1, 방문5에서 Colposcopic biopsy를 실시한다. 기저 대비 3개월 후에 측정된 치유 여부에 대해서 시험군과 대조군을 비교한다.

본 연구의 자궁경부상피이형증에서 변화의 단계는 다음과 같이 정의 한다.

-Regression: CIN I 단계에서 정상으로 치유된 경우
  →CIN I 에서 Normal
-Persistence: CIN I 단계의 변화가 없이 동일한 경우
  →CIN I 에서 CIN I
-Progression: CIN I에서 1단계 이상 증가하여 진행된 경우
  →CIN I에서 CIN II, CIN III, SCC(Squamous cell carcinoma)

15.2.2 2차 유효성 평가변수

1) 약물 투여 전 대비 투여 후 Reid Colposcopic Index 비교

Reid Colposcopic Index는 CIN 병변에 대한 여러 소견들을 특징에 따라 분류하고 점수화한 Grading System이다. 점수는 0에서 8점까지 채점되며 점수에 따른 Grading은 아래와 같이 판정된다.

0-2점: Likely to be CIN I

3-4점: Overlapping lesion - likely to be CIN I or CIN II

5-8점: Likely to be CIN II

2) 약물 투여 전 대비 투여 후 Pap Smear Test 결과 비교

자궁경부 세포검사는 two-slide technique을 이용하여 자궁내구와 자궁외구 cytobrush를 이용하여 검체를 채취하여 판독기준은 **The 2001 Bethesda System for Reporting Cervical Cytologic Diagnoses** 에 의한다. 판독결과는 Negative, ASC-US, ASC-H, LSIL, HSIL, Invasive cancer(Cervical carcinoma)의 6단계로 판독 한다.

3) 약물 투여 전 대비 투여 후 HPV DNA Chip Test 결과 비교

자궁경부 세포검사 시 자궁내구와 자궁외구 cytobrush를 이용하여 채취한 검체를 사용한다.

종양상피세포와 HPV 감염 여부 및 virus type의 상관관계를 확인하기 위하여 실시 한다

방문1, 방문5에서 실시하며 HPV의 양성과 음성으로 분류하며 양성인 경우 HPV 16, 18, 31, 33, 35, 39, 45, 51, 52 등의 HPV type을 기록한다.

4) 약물 투여 전 대비 약물 투여 후 HPV HC II assay 결과 비교

자궁경부 세포검사 시 자궁내구와 자궁외구 cytobrush를 이용하여 채취한 검체를 사용한다. 자궁경부 상피내 종양의 중증도를 예측할 수 있는 방법의 하나로서 시행되는 검사이다. 검사결과는 시료의 빛의 단위를 기준으로 Positive 또는 Negative로 분류하며 Positive인 경우 Low, Intermediate, High로 기록한다

5) 약물 투여 전 대비 약물 투여 후 자연살해세포(NK cell) activity의 결과 비교

방문2와 방문3, 방문4, 방문5에서 채혈하여 12.2.8 1)의 측정방법에 따라 평가 한다.

최근의 CD107a marker를 이용한 NK 활성 측정 방법으로 변경 Ex vivo test로서, 임상시험대상자로부터 채혈된 말초혈액에서 먼저, 단핵구를 분리한다. 자연살해(NK, natural killer)세포의 활성을 측정하기 위하여 K562세포를 자연살해 세포의 표적세포로 사용한다. 표적세포를 FACS tube에 4X105cell/mL 농도로 100μL 접종한 후 말초혈액 단핵세포를 표적세포와 10:1의 비율이 되도록 FACS tube에 첨가한다. 자연살해 세포 활성은 표적세포인 K562 세포를 사멸시킬 때 자연살해 세포 표면에 나타나는 단백질 표지를 측정하여 계산한다.

6) 약물 투여 전 대비 약물 투여 후 말초혈액 단핵세포의 표현형 변화(MHC class II) CD8, CD56 population의 결과 비교

방문2와 방문3, 방문4, 방문5에서 채혈하여 12.2.8 2)의 측정방법에 따라 평가 한다

임상시험대상자의 말초혈액을 채혈한 뒤 ficoll-hypaque의 비등차 용액을 이용한 density gradient법으로 말초혈액 단핵 세포(PBMNCs)를 분리한다. 약술하면 SEPMATE tube (stemcell, 15450) 를 이용하여 혈액시료를 Histopaque (비중 1.077 g/ml sterile-filtered, Sigma)용액 위에 중첩시키고 1200Xg에서 10분간 원심분리한 후 상층을 분리하여 PBS+2% FBS 배지로 2회 세척하여 단핵구를 분리한다.

Ex vivo test로서 유세포 분석기(BD-true counter이용)를 통하여 말초 혈액 단핵세포의 면역 표현형 변화를 확인하기 위하여 임상시험대상자의 말초 혈액 단핵구(PBMNCs)를 FACS buffer (0.1% Bovine serum albumin, 0.01% sodium azide, PBS)으로 2회 세척한 다음 FACS buffer 50 ul 에 여러가지 형광이 결합된 특이 항체 (CD3-Percp-eFluor 710, CD8-FITC, CD56-PE, CD14-APC-eFluor 780, CD19-APC-eFluor 780, CD107a-AlexaFluor647) 를 첨가하여 얼음에서 30분간 방치한다.

형광 염색된 세포를 FACS buffer 로 2회 세척한 후 2% parafomaldehyde 가 포함된 인산 완충액으로 고정시킨 다음, 세포의 형광을 유세포 측정기(Gallios, Beckman, USA)를 이용하여 측정하고 분석한다.

15.3 통계분석 방법

15.3.1 분석의 일반적인 원칙

본 연구는 자궁경부상피이형증(CIN)의 가임여성을 대상으로 폴리감마글루탐산(γ-PGA)을 경구투여시, 자궁경부상피이형증 자연치유율 대비 치유율의 증가(면역치료 효과)를 확인하고, 안전성을 확인하는 2상 임상연구로 계획되었다. 본 연구에서 얻어지는 모든 자료는 연속자료의 경우 평균±표준편차, 범주형 자료는 대상자수(분율)로 정리요약하기로 하고, 필요시에는 다양한 요약통계량을 제시하기로 한다. 만약 구간추정치를 제시할 필요가 있을 경우 95% 신뢰구간을 제시하기로 한다.

본 자료의 통계적 분석에 있어 제 1 차 유효성 평가 기준인 치유율은 15.2에 정의한 적절한 유효성 평가 분석군에서 Jung (2008)의 방법에 기초하여 유의수준 5%에서 단측검정으로 실시하기로 한다. 1차 유효성 평가항목 이외의 분석에서는 유의수준 5%에서 양측검정을 실시하기로 한다. 15.3.4 와 15.3.6에서 제시된 유효성 평가 방법 및 안전성 평가 방법 외에 임상시험대상자의 연령, 위증도, 기저 실험실 수치 등 결과변수에 기여하는 예후 인자에 대해 군간 차이가 확인될 경우에는 예후인자를 보정하는 적절한 통계적 방법(예: logistic 모형, ANOCOVA, 일반화 선형 모형 등)을 추가적으로 적용하기로 한다.

15.3.2 분석군의 정의

본 임상시험에서 유효성과 안전성을 평가하는데 필요한 분석집단은 국내 및 국제 기준에 따라 유효성 평가에 있어서는 아래에 정의하는 바와 같이 ITT 및 PP분석군에서 모두 실시하고, 안전성 평가에 있어 아래의 정의에 따라 분석을 정의하고 이를 기준으로 평가한다.

유효성 평가 분석군:

- ITT(Intent-to-Treatment)군: 연구참여 동의 후 확률화 배정된 모든 대상자로 정의한다. 다만

ITT 군에서 연구약물을 최소 1번이라도 복용하지 않은 경우는 분석군에서 제외하기로 한다.

- PP(Per Protocol)군: 연구참여 동의 후 연구의 종료 시까지 14.2에서 제시한 중대한 연구계획서 위반 사항이 없이 연구계획에 따라 임상연구를 완료한 대상자 전체로 정의한다.

- 유효성 평가에 ITT군과 PP군에서 모든 분석을 실시 및 결과보고에 제시하기로 하고, 최종 평가는 ITT군의 분석결과에 따라 결정하기로 한다. 만약 ITT군과 PP군의 결과가 상이할 경우, 두 분석결과가 상이한 이유를 탐색하는 추가적인 분석을 실시하기로 한다.

안전성 평가 분석군:

연구 참여에 동의한 후 확률화 배정되어 최소 1회 이상 연구약물을 복용한 대상자 전체로 정의하기로 한다.

15.3.3 결측치 처리방법

유효성 평가변수의 분석에 있어 결측이 발생할 경우 LOCF(Last Observed Carried Forward)방법을 적용하여 결측값을 대치하기로 하고, 안전성 평가 변수에 대해서는 결측이 발생될 경우 대치를 실시하지 않고 분석하기로 한다.

15.3.4 일차 유효성 평가변수에 관한 분석

본 연구에서 1차 유효성 평가변수는 15.2.1에서 기술한 것과 같이 기저에서 CIN I의 약물 투여 3개월째에 정상으로 치유된 경우로 판정하고 시험군과 대조군 간의 치유율을 비교하는 것이다.
각 군간 치유율의 평가는 Jung (2008)이 제시한 방법에 기초하는데 앞에서 기술한 것과 같이 1단계에서(중간분석) 각 군당 43 명을 선정하여 결과를 확인하였을 때, 치유에 해당하는 임상시험대상자의 수의 차이가 2명 미만(시험군-대조군)인 경우 폴리감마글루탐산의 효과가 없음으로 판정하고, 본 연구는 종료된다. 만약 1단계에서 2명이상 차이가 발생할 경우로 추가로 각 군당 37명을 모집하여 총 각 군당 80 명을 대상으로 치유로 판정받은 임상시험대상자의 수가 11 명 이상이면 자궁경부상피이형증의 치유에 있어 폴리감마글루탐산의 효과가 있을 것으로 판단하게 된다. 만약 11 명 미만인 경우 폴리감마글루탐산의 효과는 최종적으로 없는 것으로 결론짓게 된다.

만약 2단계 종료 후 각 분석군에서 최종 대상자 수가 다를 경우 Jung(2008)에서 제시한

$$\alpha = \sum_{k_1=a_1}^{n_1} \sum_{y_1=\max(0,-k_1)}^{n_1-\max(0,k_1)} \sum_{k_2=a-k_1}^{n_2} \sum_{y_2=\max(0,-k_2)}^{n_2-\max(0,k_2)} b(y_1|n_1, p_0)b(k_1+y_1|n_1, p_0) \times b(y_2|n_2, p_0)b(k_2+y_2|n_2, p_0)$$

여기서,

n1, n2: 1, 2단계에서의 각 군당 대상자수
a1, a: 귀무가설을 채택 여부를 판단하는 1단계 및 최종단계 치유된 대상자 수의 군간 차이
y1, y2, x1, x2: 두 군에서 각 단계의 확률변수
b(y|n, p): 이항분포의 확률분포

유의수준 공식에 근거하여 p-값을 제시하기로 한다.

추가적으로, 기저 진단 시 자녀수, 성관계 파트너수 그리고 흡연여부를 공변량으로 하여 군간 치유율의 차이에 대해 로지스틱 회귀분석을 통하여 "탐색적 분석"으로 실시하기로 한다.

15.3.5 이차 유효성 평가변수에 관한 분석

2차 유효성 평가변수 1: **Reid Colposcopic Index 비교**

Reid Colposcopic Index는 CIN 병변에 대한 여러 소견들을 특징에 따라 분류하고 점수화한 Grading System로 0에서 8점까지 채점되며 점수에 따른 Grading은 아래와 같이 판정된다.

0-2점: Likely to be CIN I

3-4점: Overlapping lesion - likely to be CIN I or CIN II

5-8점: Likely to be CIN II

Reid Colposcopic Index의 분석에 있어 기저의 점수 대비 3개월 후의 점수의 차이에 대한 군간 비교를 이표본 t-검정 또는 윌콕슨 순위합 검정을 통하여 확인하기로 한다. 또한 Grading체계(0-2점, 3-4점, 5-8점)를 multinomial 분포로 고려하여 기저 및 연구 종료 시에서의 군간 비교를 카이제곱 또는 정확검정을 통해 수행하고, 기저 대비 3개월 후의 Grading체계 변화의 군간 차이를 누적 또는 순서 로지스틱 모형(cumulative or ordinal logistic model)을 통해 분석하기로 한다.

2차 유효성 평가변수 2: **Pap Smear Test 결과 비교**

자궁경부 세포검사는 자궁내경관에 brush(broom)의 긴 중앙부분을 넣어 동일한 방향으로 2~3회 회전 후 보존액이 담겨진 용기 안에 brush를 분리시켜 넣고 뚜껑을 닫는다. 판독기준은 **The 2001 Bethesda System for Reporting Cervical Cytologic Diagnoses** 에 의한다. 판독결과는 Negative, ASC-US, ASC-H, LSIL, HSIL, Invasive cancer(Cervical carcinoma)의 6단계로 나뉘는데, multinomial 분포로 고려하여 기저 및 연구종료 시에서의 군간 비교를 카이제곱 또는 정확검정을 통해 수행하고, 기저대비 3개월 후의 분류단계의 변화를 확인하기 위해 누적 또는 순서 로지스틱 모형(cumulative or ordinal logistic model)을 통해 분석하기로 한다.

2차 유효성 평가변수 3: **HPV DNA Chip Test 결과 비교**

HPV DNA Chip Test는 바이러스의 감염타입을 확인하기 위해 실시하는 시험이다. 시험결과에 의해 Positive 또는 Negative로 분류하게 되는데, 이항 분포로 고려하여 기저 및 연구종료 시에서의 군간비교를 카이제곱 또는 정확검정을 통해 수행하고, 기저 대비 3개월 후에 측정된 치유 여부에 대해서 시간의 따른 변화와 군간 차이 그리고 시간 및 군간 상호작용을 확인하기 위하여 GEE(Generalized Estimating Equation) 또는 일반화 선형 혼합 모형(Generalized Linear Mixed Model)을 고려하기로 한다.

2차 유효성 평가변수 4: **HPV HC Ⅱ assay 결과 비교**

HPV HC(Hybrid Capture) Ⅱ assay 는 자궁경부 상피내 종양의 중증도를 예측할 수 있는 방법의 하나로서 시행되는 검사이다. 검사결과는 시료의 빛의 단위를 기준으로 Positive 또는 Negative로 분류되는데, 이항 분포로 고려하여 기저 및 연구종료 시에서의 군간 비교를 카이제곱 또는 정확검정을 통해 수행하고, 기저 대비 3개월 후에 측정된 치유 여부에 대해서 시간의 따른 변화와 군간 차이 그리고 시간 및 군간 상호작용을 확인하기 위하여 GEE(Generalized Estimating Equation) 또는 일반화 선형 혼합 모형(Generalized Linear Mixed Model)을 고려하기로 한다.

2차 유효성 평가변수 5: **자연살해세포(NK cell) activity의 결과 비교**
기저 대비 1개월, 2개월 및 3개월 후에 측정된 자연살해세포(NK cell) activity의 결과 비교를 위해 기저 및 1개월, 2개월과 3개월 후의 자연살해세포(NK cell) 수치 차이에 대한 평균 비교를 t-검정 또는 윌콕슨 순위합 검정을 통해 수행할 것이다.

2차 유효성 평가변수 6: **말초혈액 단핵세포의 표현형 변화 CD8, CD56 population의 결과 비교**
기저 대비 1개월, 2개월 및 3개월 후에 측정된 말초혈액 단핵세포의 표현형 변화(MHC class II) CD8, CD56 population의 결과 비교에 있어 표현형의 변화에 대해 기저 및 1개월, 2개월과 3개월 후의 변화 여부에 대해 군간 비교를 위해 카이제곱 검정 또는 정확 검정법을 사용하고, 기저 대비 3개월 후에 측정된 치유 여부에 대해서 시간의 따른 변화와 군간 차이 그리고 시간 및 군간 상호작용을 확인하기 위하여 GEE(Generalized Estimating Equation) 또는 일반화 선형 혼합 모형(Generalized Linear Mixed Model)을 고려하기로 한다.

15.3.6 안전성 평가변수에 관한 분석

대상자로부터 수집된 모든 이상반응, 임상 실험실적 검사치, 12-lead ECG, 및 활력 징후(혈압 및 맥박수)등 모든 자료에 대해 안전성 평가를 수행한다.

1) 이상반응

시험약 투여 후 관찰된 이상반응을 요약한다. 이상반응, 이상약물반응, SAE, 사망, 임상시험 중단을 초래한 이상반응, 및/또는 "기타 유의한 이상반응(OAE)"을 경험한 임상시험대상자의 수, 발생건수를 각 치료군 별로 요약한다. 각 이상반응을 나타낸 임상시험대상자 수 또한 각 치료군 별로 신체기관계(SOC), 권장용어 및 최대 중증도 별로 요약한다. 요약 통계량 이외에 군간 이상반응 발생비율 또는 발생건수는 Chi-squared Test, Fisher`s Exact Test 또는 Poisson test를 이용하여 분석한다.

2) 활력징후 및 실험실 검사

실험실적 검사치, 12-lead ECG의 이상치 발현 정도의 각 시점별로 군간 비교를 위해 Chi-squared Test, Fisher's Exact Test 또는 Poisson test를 이용하여 분석하고, 활력징후에 대해서도 매 시점별로 연속형 자료의 요약통계량을 제시하고, 필요시 GLM 또는 GLMM 방법을 이용하여 군간 비교를 실시하기로 한다.

[16] 부작용을 포함한 안전성의 평가기준, 평가방법 및 보고방법

16.1 이상반응 평가

이상반응은 약품 투여가 이루어진 시점부터 면밀히 관찰하며, 이후 방문 시에 시험담당자의 면담 및 문진 등 진료를 통하여 확인하며, 발현일 및 소실일, 이상반응의 정도 및 결과, 임상시험용 의약품과 관련하여 취해진 조치 및 임상시험용 의약품과의 인과관계, 임상시험용 의약품 이외의 의심되는 약제명, 이상반응에 대한 치료 여부 및 내용 등을 상세히 기록한다. 이상반응의 발생 시 즉시 연구자에게 보고하고 내원하여 진료를 받을 수 있도록, 임상시험대상자를 교육하고 임상시험대상자에게 연구자의 연락처를 제공하여야 한다. 세부적인 이상반응의 평가와 기록 및 처치는 16.3항에 따른다.

16.2. 예측되는 이상반응

선행된 임상시험에서 유의할 만한 유해사례가 관찰되지 않았다.

16.3 자・타각 증상 등 이상반응의 평가방법 및 평가기준

이상반응이란, 임상시험용 의약품과의 관련여부와 상관없이 시험도중 발생하는 모든 바람직하지 않은 해부, 생리학적 병변이나 대사기능의 병변으로 인해 나타나는 신체증상, 징후 및 임상 검사치의 변화 등을 말한다. 여기에는 기존상태의 악화, 병발질환, 약물상호작용 등도 포함된다.
본 임상시험에서는 방문2의 임상시험용의약품의 투여 이후 실시하는 실험실적 검사와 활력징후 측정, 심전도 검사에서 정상범위를 벗어난 경우, 연구자는 결과치의 임상적 유의성을 판단하여 임상적으로 유의한 결과에 대한 소견과 함께 이상반응으로 보고 한다. 임상시험 중 발생하는 모든 이상반응을 기록하는 것은 임상시험 책임자와 담당자의 의무이다. 이상반응은 의학진단용어로 기록하여야 하며, 이것이 불가능할 경우 임상시험 책임자 또는 담당자가 관찰하거나, 임상시험대상자가 보고한 증상 및 징후에 대한 용어를 기록한다.
증례기록서에는 이상반응의 증상 및 징후, 지속시간 (시작 및 종료 날짜), 중증도, 임상시험용 의약품과의 인과관계, 이상반응에 관련하여 취해진 조치, 중대한 이상반응의 여부, 결과 등에 관하여 기록한다.

1) 이상반응의 중증도

이상반응의 중증도는 CTCAE v4.0에 따라 Grade 1~5 의 5단계로 나누어 평가하되, Grade 4 또는 5에 해당하는 경우는 중대한 이상반응으로 간주한다. 다음은 CTCAE v4.0의 일반적인 예이다.

1 = 경증 (Mild)	무증상 또는 경증의 증상: 일상적 또는 진단적 관찰만 가능. 치료적 중재는 필요하지 않음
2 = 중등도 (Moderate)	최소, 국소적 또는 비침습성 치료가 필요함: 식사준비나 쇼핑 등의 일상적인 활동 제한
3 = 중증 (Severe or medically significant but not immediately life-threatening)	중증 또는 의학적으로 유의하거나, 즉각적으로 생명의 위협을 초래하지 않음: 입원이나 입원 기간의 연장, 불구, 누워있지 않으나 목욕, 옷 갈아입기, 식사, 용변 등의 스스로 돌보는 일상 활동이 제한됨
4 = 생명을 위협함 (Life-threatening consequences)	즉각적인 치료가 필요함
5 = 사망 (Death related to AE)	사망과 관련된 이상반응

2) 임상시험용 의약품과의 인과관계

이상반응 발현 시 임상시험용 의약품과의 연관성 여부는 담당 연구자가 하기와 같이 분류하고, 견해를 기록한다.

평가 단계	평가 기준
확실함 (Certain)	약물 투여와 이상반응 발현의 전후관계가 타당한 경우<br>이상반응이 다른 의약품이나 화학물질 또는 수반하는 질환으로 설명되지 않는 경우<br>투여 중단 시 임상적으로 타당한 반응을 보이는 경우<br>재투여 시 (가능한 경우에만 실시) 약물학적 또는 현상학적으로 결정적인 경우
상당히 확실함 (Probable/Likely)	약물 투여와 이상반응 발현의 시간적 관계가 합당한 경우<br>이상반응이 다른 의약품이나 화학물질 또는 수반하는 질환에 의한 것으로 보이지 않는 경우<br>투여 중단 시 임상적으로 합당한 반응을 보이는 경우<br>재투여 정보는 없는 경우
가능함 (Possible)	약물 투여와 이상반응 발현의 시간적 관계가 합당한 경우<br>이상반응이 다른 의약품이나 화학물질 또는 수반하는 질환에 의한 것으로도 설명되는 경우<br>투여 중단에 관한 정보가 부족하거나 불명확한 경우
가능성 적음 (Unlikely)	약물 투여와 이상반응 발현에 인과관계가 있을 것 같지 않은 일시적 사례인 경우<br>이상반응이 다른 의약품이나 화학물질 또는 잠재된 질환에 의한 것으로도 타당한 설명이 가능한 경우
없음 (None)	약물을 투여하지 않은 상태에서 이상반응이 발생한 경우<br>약물 투여 전 발생한 이상반응이 투여 후 악화되지 않은 경우
평가곤란 (Conditional/Unclassified)	적절한 평가를 위해 더 많은 자료가 필요하거나 추가 자료를 검토 중인 경우
평가 불가 (Inaccessible / Unclassifiable)	정보가 불충분하거나 상충되어 판단할 수 없고 이를 보완하거나 확인할 수 없는 경우

3) 이상반응과 관련하여 취해진 조치

0 = 취해진 조치 없음<br>No action taken
1 = 임상시험용 의약품의 투여용량 변경 / 일시적 중단<br>Study drug dosage adjusted / temporarily interrupted
2 = 임상시험용 의약품의 투여 중단<br>Study drug permanently discontinued due to this adverse event
3 = 치료약물 병용 투여<br>Concomitant medication taken
4 = 비약물 치료<br>Non-drug therapy given
5 = 입원/입원 기간의 연장<br>Hospitalization / prolonged hospitalization

16.4 중대한 이상반응의 평가방법 및 평가기준

중대한 이상반응/유해약물사례(Serious AE, ADR)는 시험에 사용되는 임상시험용 의약품의 임의의 용량에서 발생한 이상반응 또는 유해약물사례 중에서 다음 각목의 1에 해당하는 경우를 말한다.
1) 사망하거나 생명에 대한 위험이 발생한 경우
2) 입원할 필요가 있거나 입원 기간을 연장할 필요가 있는 경우
3) 영구적이거나 중대한 장애 및 기능저하를 가져온 경우
4) 태아에게 기형 또는 이상이 발생한 경우

또한 다음의 항목에 해당하는 경우에는 중대한 이상반응으로 간주하지 않는다.
1) 자발적인 의지로 필요하지 않은 입원을 하는 경우
2) 임상시험 참여 전 계획되어 있던 수술, 검사 등으로 입원하게 되는 경우

모든 이상반응은 근거문서와 증례기록서에 기록하며, 동일한 이상반응 용어를 사용해야 한다. 시험기간 중 “중대한 이상반응(serious adverse event)” 발생 시에는 해당 기관 임상시험심사위원회에 보고하여 시험의 지속 또는 중단 여부를 결정한다.
임상시험기간 중 중대한 이상반응/유해약물사례가 발생한 경우 시험담당자는 일단 임상시험용 의약품에 대한 시험의 일부 또는 전부를 중단하고 즉시 시험책임자 및 시험의뢰자에게 보고한다. 시험책임자는 임상시험심사위원회 및 시험의뢰자에게 발생 후 24시간 이내, 혹은 늦어도 다음 근무일까지 전화 또는 팩시밀리로 보고해야 한다. 또한, 중대한 이상반응 발생 후 7일 이내에 문서로 상세한 내용이 포함된 추가 보고를 해야 한다. 의뢰자는 기타 관련된 연구자, 심사위원회에 예상하지 못한 모든 유해사례를 의약품 임상시험 관리기준에 따라 신속히 보고하여야 한다.

1. 신속 보고 : 중대한 이상반응이 임상시험 기간 중 발생하는 경우, 임상시험용 의약품과의 관련 여부와 관계없이 시험책임자 또는 담당자는 이상약물반응 신속보고양식에 의거, 24시간 이내에 임상시험 심사위원회와 아래에 기술한 ㈜바이오리더스 또는 ㈜바이오리더스가 위탁한 임상시험 수탁기관의 모니터 요원(또는 담당자) 에게 보고하여야 한다.

*모니터 요원 (또는 담당자) : 별첨3. 모니터 요원 참고

2. 상세 보고: 중대한 이상반응 발생 후 5일 이내에 문서로 상세한 내용이 포함된 추가 보고를 하여야 한다.

3. 이상반응의 추적 관리
   시험책임자 또는 담당자는 이상반응이 나타난 임상시험대상자에 대해 증상이 가라앉고 비정상적 임상 검사치가 기준치로 회복되거나, 혹은 관찰된 변화에 대해 만족스러운 설명이 될 때까지 추적 관찰하여야 한다. 또한 이상반응의 진행 경과에 대하여 담당자에게 보고하여야 한다.

[17] 임상시험대상자 동의서 양식

임상시험대상자의 동의는 Helsinki 선언 근거한 윤리적 원칙 및 KGCP의 기준에 따라 이루어져야 하며, 시험책임자는 임상시험을 시작하기 전에 사용할 동의서 서식과 임상시험대상자설명서에 대해 임상시험심사위원회의 승인을 받아야 한다. 임상시험대상자 또는 임상시험대상자의 대리인에게 본 임상시험의 내용 및 임상시험용의약품의 효과, 이상반응에 대해 충분한 정보를 알린 후 서면으로 동의서를 받아야 한다.

임상시험대상자의 자기결정권을 존중하며 면담 실시, 설명 및 동의취득 시는 독립된 공간에서 할 수 있도록 한다.

만약 임상시험대상자와 임상시험대상자의 대리인이 문서화 된 정보를 읽을 수 없는 경우에는 입회자가 동의를 얻는 전 과정에 참석하여야 한다.

각 서명은 서명자에 의해 직접 날짜가 기재 되어야 하고 동의서와 임상시험대상자설명서는 시험책임자 및 시험 담당자가 원본을 보관하며, 서명된 동의서 사본은 임상시험대상자 또는 임상시험대상자의 대리인에게 제공한다.

또한 동의서 취득 과정에 대해서 반드시 근거문서에 기록을 남기도록 한다.

환자의 일상적인 진료가 아닌 임상시험의 어떠한 행위 또는 치료가 시작되기 전에 임상시험대상자 또는 임상시험대상자의 대리인에게 동의서를 받는 것은 연구자의 책임이다.

별첨5 임상시험대상자 설명서 및 동의서

[18] 임상시험대상자 보상 규약

피해자 보상에 대한 규약

1. 본 임상시험 기간 동안 임상시험대상자에게 발생하는 피해에 관하여 아래 항목 중 한 항목에 해당할 경우 보상을 한다. 단, 보상을 하는 경우는 임상시험대상자에게 발생하는 피해와 연구 방법 또는 임상시험에 투여된 임상시험용 의약품 사이에 인과관계가 없음을 입증하지 못하는 경우로 제한된다.

- 합의된 임상시험계획서에 따라 임상시험용 의약품 사용에 의한 피해
- 합의된 임상시험계획서에 따라 수행된 진단과정의 결과로 발생한 피해
- 예기치 않은 부작용의 결과 합법적으로 요구되는 치료 또는 진단방법의 결과 발생된 피해

2. 다음의 경우에는 보상하지 아니한다.
- 본 임상시험에서 제공하지 않은 약물로 의해 발생한 부작용에 의한 손상
- 본 임상시험용 의약품과 무관하게 임상시험대상자가 원래부터 가지고 있었던 질환이나 병발 질환에 기인한 손상
- 연구자가 서로 합의된 임상시험계획서에 따르지 않음으로써 야기된 손상
- 임상시험대상자의 부주의에서 초래된 손상

3. 임상시험책임자, 담당자는 임상시험대상자가 본 임상시험에 의해 어떠한 불이익이라도 받지 않도록 관계 법규와 규범, 상호 합의한 임상시험계획서의 내용을 충실히 준수하는 등 최선을 다해야 한다.

그러나 이러한 노력에도 불구하고 임상시험 도중 본 연구에서 제공한 임상시험용 의약품에 의해 임상시험대상자가 피해(상해 또는 부작용 발생)를 입게 된 경우 아래의 보상평가기준에 따라 합리적인 치료비 또는 보상금을 지급한다.

<보상평가 기준>

치료비 또는 보상금은 피해의 본질, 정도, 기간, 지속성여부 등에 따라 이를 치료 또는 보상할 수 있는 적절한 액수여야 하며 한국법정에서 유사피해에 대해 일반적으로 지급되도록 하는 것과 동일한 수준으로 한다. 보상수준에 대해 임상시험대상자와 시험조정자 사이에 이견이 있을 경우에는 우선적으로 양 당사자의 합의 하에 선정한 전문가로부터 자문을 구하도록 하고, 자문을 받을 경우에는 자문내용에 따르기로 한다.

본 임상시험에서 임상시험대상자가 입는 피해에 대하여 상기 내용에 의거하여 책임질 것을 서약합니다.

년 월 일

의뢰사: ㈜바이오리더스 (인)

[19] 임상시험 후 임상시험대상자의 진료 및 치료기준

자궁경부상피이형증은 추후 관찰이 중요하므로 ASCCP 2006의 권고안에 따라 정기적인 검진을 받도록 한다.
병변이 진행이 되었을 경우 자궁경부 이형증의 일반적인 치료방법인 원추절제술을 실시하며 정기적인 검진으로 자궁경부암 진행의 예방 및 조기발견을 할 수 있도록 권고 한다

일반적인 치료로 국소적인파괴요법으로 세포 내 수액을 결정화(crystallization)함으로써 세포괴사를 유발하여 자궁경부 상피 표면층을 파괴한다. 마취가 필요 없고 안전하고 효과적인 냉동요법(cryosurgery), 자궁경부 상피세포에 레이저 빔을 쪼여 기화시키는 방법으로 병적인 변화가 나타난 부위가 아주 넓거나 질 부위로 확산된 경우에도 사용이 가능한 레이저 요법, 100도에서 20초 정도 가열하는 방법으로 치료 효과는 냉동요법과 비슷한 냉응고법(cold coagulation)이 있다.

절제술로는 와이어 루프(Wire loop)를 이용하여 전체 변형대를 제거하는 방법이다. 제거된 조직을 이용하여 진단과 치료를 1회의 시술로 모두 할 수 있다는 장점이 있으며, 쉽고 신속하게 시행할 수 있어 외래에서 시술이 가능한 환상투열요법(loop electrosurgical excision procedure, LEEP), 냉도(cold knife)나 이산화탄소 레이저(CO2 laser) 혹은 전기절제기구(electrosurgical instrument)를 이용하여 절제하는 방법으로 진단과 치료를 목적으로 실시할 수 있는 원추절제술(conization), 자궁경부 상피내 종양 치료 방법 중에서 가장 재발률이 낮은 방법으로는 자궁절제술이 있다.
세포 단독검사 혹은 질확대경 검사를 이용한 추적검사를 시행하여 일정기간 이상 검사 결과가 음성으로 나올 경우 매년 정기적인 세포검사를 시행한다. 치료적 시술에 의한 합병증은 환상투열요법이나 원추절제술 이후 출혈, 감염, 경관유착이나 자궁경부무력증, 자연유산, 조기진통, 저체중아 분만 등이 발생할 수 있다는 보고가 있다.
탈락된 임상시험대상자가 다른 적절한 치료를 받을 수 있도록 지도하며 임상시험 종료된 임상시험대상자는 예측하지 않았던 이상반응 발생에 대비하여 임상시험책임자 및 담당자의 지시에 따라 진단 및 적절한 치료를 받을 수 있도록 한다.

[20] 임상시험대상자의 안전보호에 관한 대책

1) 임상시험 참여 전, 검사를 통하여 임상시험대상자가 본 연구에 적절한지를 엄격히 평가한다. 특히 가임기 여성을 대상으로 실시하므로 Urine hCG 검사가 누락되지 않도록 한다. 검사 결과 임신반응이 음성으로 나온 자를 선정하며, 임상시험기간 동안 피임을 유지하도록 한다.

2) 어떠한 이상반응이라도 Baseline 익일 이후, 즉 임상시험용 의약품을 복용한 이후 발생한 증상, 징후, 질병에 대해서는 임상시험대상자가 이를 연구자에게 보고하도록 교육한다.

3) 임상시험 계획서에 따라 임상시험을 실시하고 시험기간 중 방문 시 검사와 검진을 통하여 이상반응 및 유해약물사례의 출현 여부와 그 정도를 평가하고 적절한 조치를 취한다.

[21] 그 밖에 임상시험을 안전하게 과학적으로 실시하기 위하여 필요한 사항

21.1 자료의 질 보증(Data Quality Assurance)과 자료관리 계획

움트 및 통계를 담당하는 전문기관에서 본 시험의 자료 관리를 담당할 것이다. 작성된 증례기록서의 검토 및 데이터베이스에 입력한 자료를 전산 프로그램을 이용한 logical check으로 자료의 정확성, 완전성, 일관성, 논리적임을 확인하고 보증하는 작업을 수행할 것이며, 자료관리 과정에서 발견된 모든 문제점을 해결할 수 있도록 연구자와 긴밀하게 연락을 취할 것이다.

21.2 임상시험관리기준(KGCP)

본 시험을 실시함에 있어 KGCP 및 Helsinki 선언의 근본 정신을 준수하여 윤리적이고 과학적인 배려 하에 연구를 실시하도록 한다. 임상시험대상자에게 임상시험에 대한 권유를 할 때에는 독립된 공간에서 임상시험의 이득과 발생할 수 있는 위험성에 대하여 충분한 설명을 한 후 면담을 실시하여 동의를 취득하여야 한다.

21.3 근거 자료의 직접열람

본 시험 참가자의 안전을 보장하고 정확하고 완벽하며 신뢰 가능한 데이터를 얻기 위해, 연구자는 임상검사 결과, 임상기록, 임상시험대상자의 의학 기록을 근거문서로 보관하여야 한다. 연구자는 의뢰자, 식품의약품안전처 및 임상시험심사위원회의 요청이 있을 경우 시험 관련 자료 직접열람(direct access)을 허용해야 한다.

21.4 모니터링

㈜바이오리더스에서 지명한 모니터요원은 시험의 진행을 모니터링하기 위한 목적으로 정기적으로 적절한 간격을 두고 시험기관을 방문하여 증례기록서와 근원문서(source documents)를 비교하는 임무를 수행하게 된다. 연구자는 임상시험용 의약품의 보관 장소, 시험에 관련된 서류에 대해 시험 모니터요원 또는 본 업무의 위임자가 접근할 수 있도록 동의하고 협조하여야 한다. 모니터링은 움트와 ㈜바이오리더스에서 기본적으로 진행한다.

21.5 증례기록서(Case Report Form)

임상시험대상자의 근원문서(source documents)라 함은 시험 기관에 보관되는 담당의사의 환자기록을 의미한다. 대부분의 근원문서(source documents)는 병원이나 담당의사의 차트이며 이러한 경우 환자의 전자 증례기록서에 수집되어 기록된 모든 정보는 해당 차트와 일치해야 한다.
기록될 데이터가 얻어지면 가능한 신속히 증례기록서를 작성해야 한다. 증례기록서를 기록, 검토, 서명하는 것은 연구자의 임무이다. 증례기록서를 작성한 후 연구자는 개개의 증례기록서에 서명함으로써 증례기록서에 기록된 정보가 사실임을 보증한다. 연구자는 모든 경우에 있어서 증례기록서에 기록된 모든 임상치와 실험실치의 정확성과 신뢰성에 대한 최종적인 책임을 갖는다.

21.6 연구 자료의 기밀유지 및 임상시험 관련자료 보관 규정

임상시험대상자 식별정보는 코드화 하여 개인정보의 유출을 방지하며 연구기간 동안 연구 자료는 접근이 제한된 컴퓨터나 잠금 장치가 있는 곳에 보관한다. 시험기관의 장은 임상시험심사위원회 및 (주)바이오리더스에 제출된 보고서를 포함한 시험에 관한 모든 문서를 임상종료일로부터 3년간 보관 하도록 한다.

21.7 시험 계획서의 변경

시험계획서를 임상시험심사위원회로부터 승인 받은 후, 시험절차가 더 광범위해지거나 위험도가 높아지거나 환자 선정기준에 변화가 있거나 추가적인 안전성 정보로 인해 임상시험 계획서를 변경하는 경우에는 임상시험심사위원회의 승인을 받아야 한다. 시험 계획서를 수정할 때에는 개정 일자, 개정 이유, 개정 내용 등을 기록하여 보관하여야 한다.

오자를 교정하거나 보다 명백히 하기 위해 단어를 바꾸거나, 모니터가 바뀌거나 통계학적 분석에 관한 계획의 수정처럼 시험 진행에 영향을 미치지 않을 사소한 변화에 한해서는 승인이 반드시 필요한 것은 아니다.

21.8 계획서 위반

의학적으로 응급한 상황을 제외하고는 연구자와 ㈜바이오리더스 간의 사전 동의 없이 최종적으로 서명된 시험 계획서를 위반하는 것은 허용되지 않을 것이다.

만약 계획서의 위반사항이 임상시험대상자의 시험 지속 여부를 결정해야 하는 중대한 위반사항인 경우 연구자 또는 모니터는 그 내용을 계획서 위반 사항 작성 양식(protocol deviation form)에 기록하여 ㈜바이오리더스에 송부하고, 그 임상시험대상자의 시험 지속여부에 대한 ㈜바이오리더스의 승인을 받아야 한다.

[22] 참고문헌

[1] Laimins LA. Humanpapillomaviruses target differentiating epitheria for virion production and malignant conversion. Semin Virol 1996;7:305-13

[2] Depuydt CE, Vereecken AJ, Salembier GM et al.Thin-layer liquid-based cervical cytology and PCR for detecting and typing humanpapillomavirus DNA in Flemish woman. Br J Cancer 2003;88:560-6

[3] Van Hentenryck M, Noel JC, Simon P. Obstetric and neonatal outcome after surgical treatment of cervical dysplasia. Eur. J. Obstet. Gynecol. Reprod. Biol. (2012) 162: 16-20

[4] Lee TY, Kim YH, Yoon SW, Choi JC, Yang JM, Kim CJ, Schiller JT, Sung MH, Poo H. Oral administration of poly-gamma-glutamate induces TLR4- and dendritic cell-dependent antitumor effect. Cancer Immunol. Immunother. (2009) 58: 1781-1794

[5] Tomas C. Wright Jr, MD;L. Stewart Massad, MD;Charles J.Dunton, MD;Ma가 Spitzer, MD;EdwardJ.Wilkinson,MD; Diane Solomon,MD;for the 2006 American Society for Colposcopy and Cervical Pathology-sponsored Consensus Conference

[6] Saw HS, Lee JK, et al. Natural History of Low-Grade Squamous Intraepithelial Lesion (Journal of Lower Genital Tract Disease, Volume5, Number3,2001,153-158)

[7] Jung SH. Randomized phase Ⅱ trials with a prospective control. Statist. Med. (2008) 27: 568-583

[8] Barnett et al. A randomised, double-blind, placebo-controlled trial of photodynamic therapy using 5-aminolaevulinic acid for the treatment of cervical intraepithelial neoplasia. International Journal of Cancer 2003;103:829-832.

[9] Trimble et al. Spontaneous Regression of High-Grade Cervical Dysplasia: Effects of Human Papillomavirus Type and HLA Phenotype. Clin Cancer Res July 1, 2005 11; 4717.1

[10] Bansal N, Wright JD, Cohen CJ, Herzog TJ. Natural History of Established Low Grade Cervical Intr aepitherial (CIN1) Lesions. Anticancer Res. (2008) 28 : 1763-1766

[11] Hefler L, Grimm C, Tempfer C, Reinthaller A. Treatment with Vaginal Progesterone in Women with Low-grade Cervical Dysplasia. A Phase Ⅱ Trial. Anticancer Res. (2010) 30 : 1257-1262

[12] Bentley J. Colposcopic management of abnormal cervical cytology and histology. J Obstet Gynaecol Can. 2012;34(12):1188-202.

[13] Teresa M. Darragh, et al. The Lower Anogenital Squamous Terminology Standardization Project for HPV-associated Lesions: Background and Consensus Recommendations From the College of American Pathologists and the American Society for Colposcopy and Cervical Pathology. Int J Gynecol Pathol 2013;32(1).

[14] ANCUŢA BOICEA, et al. Correlations between colposcopy and histologic results from colposcopically directed biopsy in cervical precancerous lesions. Rom J Morphol Embryol 2012;53(3):735–741.

[15] Nu´ria-Laia Rodrı´guez-Mias, et al. Current Situation: Lower Genital Tract Pathology and Colposcopy Training in Spanish Gynecology and Obstetrics Residents. Journal of Lower Genital Tract Disease, 2013;17(1):12-16

[16] Reid R, Scalzi P. Genital warts and cervical cancer: VII. An improved colposcopic index for differentiating benign papillomaviral infections from high-grade cervical intraepithelial neoplasia. Am. J. Obstet. Gynecol. (1985) 153: 611-618

[17] 대한 산부인과학회와 국립암센터 2001, 조기검진 권고안_학회지

[18] Jae-Sun Park, M.D., Hee-sug Ryu., M.D., Suk-jun Chang,.M.D. The association of the cervical intraepitherial neoplasia and human papillomavirus viral load. Kor.Obstet. Gynecol. Vol. 48 No.13 Dec. 2005

[19] 진소영, 박상모, 김미선, 진윤미, 김동원, 이동화 Diagnostic Accuracy of Cervicovaginal Cytology in the Detection of Squamous Epithelial Lesions of the Uterine Cervix; Cytologic/Histologic Correlation of 481

Cases. (2008) 111-118

[20] Hilleman MR. Overview of vaccinology with special reference to papillomavirus vaccines. J Clin Virol 2000;19:79-90

[21] Stanley MA Immunobiology of papillomavirus infections. J Rreprod Immunol 2001;52:45-59

[22] Abe K, Ito Y, Ohmachi T, Asada Y. Purification and properties of two isozymes of γ-glutamyltranspeptidase from *Bacillus subtilis* TAM-4. Biosci. Biotechnol. Biochem. (1997) 61: 1621-1625

[23] Birrer GA, Cromwick AM, Gross RA. Poly-γ-glutamic acid formation by *Bacillus licheniformis* 9945A : physiological and biochemical studies. Int. J. Biol. Macromol. (1994) 16: 265-275

[24] Discacciati MG, de Souza CA, d'Otavianno MG, Angelo-Andrade LA, Westin MC, Rabelo-Santos SH, Zeferino LC. Outcome of expectant management of cervical intraepithelial neoplasia grade 2 in women followed for 12 months. Eur. J. Obstet. Genecol. Reprod. Biol. (2011) 155: 204-208

[25] Eom SY et al. Effect of Keumsa Sangwhang (*Phellinus linteus*) mushroom extracts on the natural killer cell activity in human. Korean J. Food Sci. Technol. (2006) 38(5): 717–719

[26] Perez-Camero G, Congregado F, Bou JJ, Munoz-Guerra S. Biosynthesis and ultrasonic degradation of bacterial poly-γ-glutamic acid. Biotechnol. Bioeng. (1999) 63: 110-115

[27] Goto A, Kunioka M. Biosynthesis and hydrolysis of poly-γ-glutamic acid from *Bacillus subtilis* IFO3335. Biosci. Biotechnol. Biochem. (1992) 56: 1031-1035

[28] Hezayen FF, Rehm BH, A Tindall BJ, Steinbuchel A. Transfer of Natrialba asiatica B1T to *Natrialba taiwanensis* sp. nov., a novel extremely halophilic, aerobic, non-pigmented member of the Archaea from Egypt that produces extracellular poly(glutamic acid). Int. J. Syst. Evol. Microbiol. (2001) 51: 1133-1142

[29] Hiroyuki T et al. A calcium supplement containing poly-γ-glutamic acid increase human calcium absorption. Nippon Nogeikagaku Kaishi. (2003) 77(5): 504–507

[30] Ing-Lung Shih, Yi-Tsong Van. The production of poly-γ-glutamic acid from microorganisms and its vari-ous applications. Bioresource Technol. (2001) 79: 207-225

[31] Ito Y, Tanaka T, Ohmachi T, Asada Y. Glutamic acid independent production of poly-γ-glutamic acid by Bacillus subtilis TAM-4. Biosci. Biotechnol. Biochem. (1996) 60: 1239-1242

[32] Kim TW, Lee TY, Bae HC, Hahm JH, Kim YH, Park C, Kang TH, Kim CJ, Sung MH, Poo H. Oral administration of high molecular mass poly-gamma-glutamate induces NK cell-mediated antitumor immunity. J. Immunol. (2007) 179: 775–780

[33] King EC, Blacker AJ, Bugg TDM. Enzymatic breakdown of poly-γ-D-glutamic acid in Bacillus li-cheniformis: identification of a polyglutamyl-γ-hydrolase enzyme. Biomacromolecules (2000) 1: 75-83

[34] Kubota H, Nambu Y, Endo, T. Convenient and quantitative esterification of poly-γ-glutamic acid pro-duced by microorganism. J. Polym. Sci. Part A: Polym. Chem. (1933) 31: 2877-2878.

[35] Kubota H, Matsunobu T, Uotani K, Takebe H, Satoh A, Tanaka T, Tanguchi M. Production of poly-γ-glutamic acid by Bacillus subtilis F-2-01. Biosci. Biotechnol. Biochem. (1993) 57: 1212-1213

[36] Kubota H, Nambu Y, Endo T. Alkaline hydrolysis of poly-γ-glutamic acid produced by microorganism. J. Poly. Sci. Chem. (1996) 34: 1347-1351

[37] Matsumoto K, Oki A, Furuta R, Maeda H, Yasugi T, Takatsuka N, Hirai Y, Mitsuhashi A, Fujii T, Iwasaka T, Yaegashi N, Watanabe Y, Nagai Y, Kitagawa T, Yoshikawa H. Tobacco smoking and regression of low-grade cervical abnormalities. Cancer Sci. (2010) 101: 2065-2073

[38] Monteiro DL, Trajano AJ, Russomano FB, Silva KS. Prognosis of intraepithelial cervical lesion during adolescence in up to two years of follow-up. J Pediatr. Adolesc. Gynecol. (2010) 23:230-236

[39] Moscicki AB, Shiboski S, Hills NK, Powell KJ, Jay N, Hanson EN, Miller S, Canjura-Clayton KL, Farhat S, Broering JM, Darragh TM. Regression of low-grade squamous intra-epithelial lesions in young women. Lancet (2004) 364: 1678-1683

[40] Sung MH, Park C, Kim CJ, Poo H, Soda K, Ashiuchi M. Natural and Edible Biopolymer Poly-γ-glutamic acid: Synthesis, Production, and Applications. Chem Rec. (2005) 5: 352-66.

[41] Tanaka T, Yaguchi T, Hiruta O, Futamura T, Uotani K, Satoh A, Taniguchi, M, Oi S. Screening for microorganism having poly-γ-glutamic acid endohydrolase activity and the enzyme production by Myrothecium sp. TM-4222. Biosci. Biotechnol. Biochem. (1993) 57: 1809-1810

[42] Tanaka T, Hiruta O, Futamura T, Uotani K, Satoh A, Taniguchi M, Oi S. Purification and characte-rization of poly-γ-glutamic acid hydrolase from a filamentous fungus, *Myrothecium* sp. TM-4222. Biosci. Biotechnol. Biochem. (1993) 57: 2148-2153

[43] Thorne CB, Gomez CG, Blind GR, Housewright, RD. Synthesis of glutamic acid and glutamyl po-lypeptide by *Bacillus anthracis*. III. Factors affecting peptide production in synthetic liquid media. J. Bacteriol. (1953) 65: 472-478

[44] Thorne CB, Gomez CG, Noyes HE, Housewright RD. Production of glutamyl polypeptide by *Bacillus subtilis*. J. Bacteriol. (1954) 68: 307-315

[45] Troy FA. Chemistry and biosynthesis of the poly(γ-D-glutamyl) capsule in *Bacillus licheniformis*. 1. Properties of the membrane-mediated biosynthetic reaction. J. Biol. Chem. (1973) 248: 305-316

[46] Torii, M. Studies on the chemical structure of bacterial glutamyl polypeptides by hydrazinolysis. J. Biochem. (1959) 46: 189-200

[47] Zanuy, D, Aleman, C, Muñoz-Guerra S. On the helical conformation of un-ionized poly (γ-D-glutamic acid). Int. J. Biol. Macromol. (1998) 23: 175-184

[23] 별첨 목록

별첨1. 공동연구자

별첨2. 임상시험용 의약품 관리자

별첨3. 모니터 요원

별첨4. 피해자 보상에 대한 규약
